# Associations of genetic factors with vascular diabetes complications: an umbrella review

**DOI:** 10.7189/jogh.15.04081

**Published:** 2025-03-21

**Authors:** Xuan Zhou, Nan Yang, Wei Xu, Xue Li, Athina Spiliopoulou, Evropi Theodoratou

**Affiliations:** 1 Usher Institute, College of Medicine and Veterinary Medicine, University of Edinburgh, Edinburgh, UK; 2 Department of Big Data in Health Science School of Public Health, and Centre of Clinical Big Data and Analytics of The Second Affiliated Hospital, Zhejiang University School of Medicine, Hangzhou, China

## Abstract

**Background:**

To comprehensively assess evidence from published systematic review and meta-analyses (SRMAs) on the genetics of vascular diabetes complications.

**Methods:**

A systematic literature search conducted in Medline and Embase identified 63 non-overlapping SRMAs. We re-conducted meta-analyses to compare diabetes with and without complications using multiple genetic models; evaluated associations using Venice criteria and Bayesian false-discovery probability (BFDP); and graded as highly credible, credible, and not credible. We also contrasted highly credible and credible associations to recent genome-wide association studies (GWASs).

**Results:**

Highly credible evidence was discovered for single nucleotide polymorphisms (SNPs) rs1024611 at *MCP-1* gene and SNP rs3025039 at *VEGF* gene with diabetic retinopathy (DR) in type 2 diabetes; SNP rs2268388 at *ACACB* gene, insertion/deletion (Ins/Del) variant at *ACE* gene, SNP rs1801133 at *MTHFR* gene, and SNP rs7903146 at *TCF7L2* gene with diabetic kidney disease (DKD) in type 2 diabetes; and SNP rs4880 at *SOD2* gene with diabetic peripheral neuropathy (DPN) in type 1 diabetes. Combining type 1 and 2 diabetes, highly credible evidence was discovered for insertion/deletion variant at *ACE* gene, SNP rs759853 at *AKR1B1* gene, SNP rs1044498 at *ENPP1* gene and DKD, and SNP rs1617640 at *EPO* gene for the combined endpoint of DR and DKD. None of these associations was directly replicated in the latest GWASs for DR and DKD, however, another SNP, rs55853916 at *TCF7L2* gene had been detected as a GWAS hit for DKD.

**Conclusions:**

This umbrella review rigorously assessed evidence on the genetics of vascular diabetes complications, complemented findings in recent GWASs and yielded insight into the optimal selection of genetic models for the design of GWASs on vascular diabetes complications. Mechanistic or bioinformatic studies are warranted to further assess the role of these genes in the pathology of vascular diabetes complications and their potential as drug targets.

**Registration:**

PROSPERO: CRD42022384423.

Diabetes mellitus is a chronic condition characterised by high levels of blood glucose (hyperglycaemia). It arises when the body either fails to produce sufficient insulin or cannot effectively utilise the insulin it produces. It is estimated that diabetes mellitus affects approximately 537 million people worldwide, with a global prevalence of 10.5% in 2021, and the numbers of people with diabetes are projected to increase to 643 million by 2030 and 783 million by 2045 [1]. Elevated blood glucose levels in individuals with diabetes put them at risk of microvascular complications (*i.e.* retinopathy, nephropathy, and neuropathy) and macrovascular complications (*i.e.* cardiovascular diseases). These complications are the leading causes of blindness, end-stage renal disease, and lower limb amputation, and represent the main cause of morbidity and mortality among people with diabetes [2–4].

Vascular diabetes complications are moderately heritable, with estimated heritability ranging from 34 to 59% for diabetic kidney disease, 18 to 52% for diabetic retinopathy (DR), and 6 to 15% for diabetic neuropathy based on single nucleotide polymorphisms (SNPs) in family studies [5]. Previous studies investigating the associations between genetic factors and the risk of vascular diabetes complications have mainly focused on the associations between SNPs located on candidate genes and the risk of vascular diabetes complications. However, these research endeavours are hindered by limited sample size, resulting in insufficient statistical power to demonstrate statistically significant effects, leading to inconclusive and inconsistent conclusions. Despite numerous efforts to address this issue through systematic review and meta-analyses (SRMAs), the predominant focus has remained on examining single SNPs or genes.

Umbrella review systematically collects and evaluates currently available evidence from multiple SRMAs, providing a bird eye’s view of published evidence [6]. It assesses and reports between-study heterogeneity, confounding, and other bias, offering more precise interpretation of effect estimates [6]. We conducted an umbrella review of SRMAs that investigated the associations of genetic factors with vascular diabetes complications, including both microvascular and macrovascular complications, and thus the primary objective was to gain a comprehensive understanding of the genetic predisposition to vascular diabetes complications.

## METHODS

The protocol was registered at the International prospective register of systematic reviews (PROSPERO) (CRD42022384423). We followed the guidelines of the Preferred Reporting Items for Systematic Reviews and Meta-Analyses (PRISMA) statement [7].

### Data sources and searches

In this umbrella review, we focused on long-term vascular complications of type 1 and type 2 diabetes, including macrovascular complications (*i.e.* cardiovascular disease, cerebrovascular disease, and peripheral vascular disease) and microvascular complications (*i.e.* nephropathy, neuropathy, and retinopathy). We carried out a systematic literature search in the Medline and Embase databases via the Ovid interface from inception to 6 October 2022, to capture SRMAs assessing the associations of genetic factors with vascular diabetes complications. Search strategy was predefined with the combination of Medical Subject Headings (MeSH) terms and free-text keywords. The details can be found in Table S1 in the **Online Supplementary Document**.

### Study selection

Two investigators (XZ and NY) independently reviewed the titles, abstracts, and full texts of all the identified publications, adhering to pre-established inclusion and exclusion criteria. Studies were included if they met the following criteria:

(i) investigation of the associations between genetic factors and the risk of vascular diabetes complications (as defined above) in individuals with diabetes;

(ii) systematic reviews with meta-analyses of observational studies;

(iii) with effect estimates for the associations with their corresponding 95% confidence interval (CIs) or with sufficient data to calculate them.

Studies were excluded if they:

(i) were not SRMAs;

(ii) were systematic review without meta-analyses;

(iii) did not report the effect estimates or without enough data to calculate them;

(iv) used intermediate outcomes to represent vascular diabetes complications;

(v) investigated interactions between different complications.

When there was more than one published SRMA on the same genetic factor and the same outcome, we only included the most recent and largest one. In some cases where the most recent one was not the largest, we explored the reason for this discrepancy. If the most recent one was restricted to prospective studies while the largest one included both prospective and retrospective studies, the most recent one was included; otherwise, the largest one was included.

### Data extraction and quality assessment

Data extraction was performed by one investigator (XZ) and independently verified by a second investigator (WX). The following information was extracted for each eligible SRMA: the first author, publication year, number of included studies, type of diabetes mellitus, ethnicity, number of events and total sample size, examined genetic factors and outcomes, reported meta-analysis estimates (*i.e.* odds ratio (OR), corresponding 95% CIs, *P*-values, and the measure of heterogeneity (*I*^2^)). The methodological quality and risk of bias of the eligible SRMAs were evaluated using the Assessment of Multiple Systematic Reviews 2.0 (AMSTAR 2.0) checklist [8]. Additionally, for each individual study included in the SRMAs, the following information was extracted: first author, publication year, type of diabetes mellitus, ethnicity, number of events and sample size in the exposed and unexposed groups, effect estimates, and corresponding 95% CIs.

### Data synthesis and analysis

To avoid overestimation of the true effect sizes of genetic factors on vascular diabetes complications, we implemented a quality control procedure. First, for those individual studies within the SRMA that included both diabetes patients without complications and healthy controls in the control groups, we restricted the analyses to compare diabetes patients with and without vascular complications. Second, we excluded component individual studies conducted in children and adolescents from the analyses as vascular complications are less common but more severe and aggressive if they occur in this age group. We also excluded component individual studies that were post-hoc analyses of randomised controlled trials from the meta-analysis to minimise inherent biases. When there were at least three component individual studies available, random-effects models in the ‘metafor’ R package were employed to perform the meta-analyses. Specifically, the DerSimonian and Laird (DL) method was used when the number of component individual studies was five or more, and the Hartung-Knapp-Sidik-Jonkman (HKSJ) method was used when the number of component individual studies was less than five, as the DL method is prone to inflated type 1 error when the number of component individual studies is small and there is moderate or substantial heterogeneity [9,10]. Different genetic models, including the allelic, dominant, recessive, heterozygous, homozygous, additive, and codominant model, were adopted in accordance with the original SRMAs. We conducted a χ^2^ test using the ‘HardyWeinberg’ R package to examine the Hardy-Weinberg Equilibrium (HWE) for biallelic factors in the control group. Sensitivity meta-analyses were conducted by excluding component individual studies that violated HWE in the control group. Heterogeneity between the component individual studies was quantified using the *I*^2^ statistic. Calculated based on the Cochran’s Q statistic and the degrees of freedom, *I*^2^ statistic describes the percentage of total variation across studies that is due to heterogeneity rather than chance [11]. In addition, small-study effects were assessed using Egger’s regression asymmetry test to investigate whether smaller studies tend to yield larger effect size estimates compared to larger studies [12]; excess significance was evaluated by a χ^2^ test to assess if the observed number of studies with significant results was greater than the expected number [13]. A significance level of 0.10 was used for both tests, indicating the presence of small-study effects or excess significance if the *P-*values were below this threshold. The statistical power of each meta-analysis was estimated by the ‘samplesizeCMH’ R package.

### Credibility of evidence

To evaluate the credibility of the observed associations, we employed the Venice criteria and Bayesian false-discovery probability (BFDP) analyses [14,15]. The Venice criteria assessed associations based on three aspects: the amount of evidence, the extent of replication, and protection from bias. The amount of evidence was evaluated using statistical power, with a grade of A, B, or C assigned for power larger than 80, 50–79, or less than 50%, respectively. The extent of replication was determined by the level of heterogeneity (*I*^2^), categorised as A (25% or less), B (25–49%), or C (50% or more). Assessing complete protection from bias comprehensively was challenging, so a starting grade of B was assigned. In contrast, a C grade was assigned to those meta-analyses with potential publications bias indicated by the small-study effects (Egger’s regression asymmetry test *P* < 0.10). The Bayesian False-Discovery Probability (BFDP) analyses were applied to evaluate the noteworthiness of an observed association [15]. Based on Bayesian theory, BFDP estimates the probability that a statistically significant finding is actually a false discovery, given a specific prior probability of true effects. This approach helps to protect against overinterpreting statistically significant findings. In this umbrella review, BFD*P* values were computed at two levels of prior probability with the ‘gap’ R package: a medium/low prior level (0.05 to 10^-3^), close to what would be expected for a candidate gene, and a very low prior level (10^-4^ to 10^-6^), close to what would be expected for a SNP identified from a hypothesis generating study design (*e.g.* genome-wide association studies, GWAS) [16]. A noteworthy threshold of 0.20 was set, indicating that the probability of a statistically significant finding being a false discovery is 0.2 [15]. Based on the *P-*values for meta-analyses, BFDP value, statistical power, and heterogeneity (*I*^2^), genetic associations were classified into three categories: ‘Highly credible’, ‘Credible’, and ‘Not credible’. Specifically, an association is graded as highly credible if the *P-*values of the meta-analysis is less than 0.05 in at least two genetic models, the BFDP value is less than 0.2 at any prior probability level, the statistical power is larger than 80%, and the heterogeneity (*I*^2^) is less than 50%. However, if the association receives a grade of C in the third aspect of Venice criteria grade, it should be interpreted with caution due to potential publication bias. The detailed criteria outlining the classifications are provided in **Table 1**.

**Table 1:** Criteria for the credibility of evidence

Category	Criteria
Highly credible	*P* < 0.05 in at least two genetic models
	BFDP value <0.20
	Statistical power >80%
	*I*^2^<50%
Credible	*P* < 0.05 in at least one genetic model
	BFDP value >0.20 or statistical power between 50 and 79% or *I*^2^>50%
Not credible	*P* > 0.05 in all genetic models

BFDP – Bayesian false-discovery probability

Finally, we generated a list of genetic variants with highly credible and credible evidence for each complication and compared the associations with those reported in the most up-to-date GWASs [17,18] (depending on data availability of the effect size and 95% CIs of each variant from the summary statistics). We performed all the statistical analysis in *R*, version 4.2.3 (R Core Team, Vienna, Austria).

### Ethical approval

Ethical approval was not required for this umbrella review because it was conducted based on data extracted from SRMAs and their component individual studies.

## RESULTS

### Literature search results

**Figure 1:**
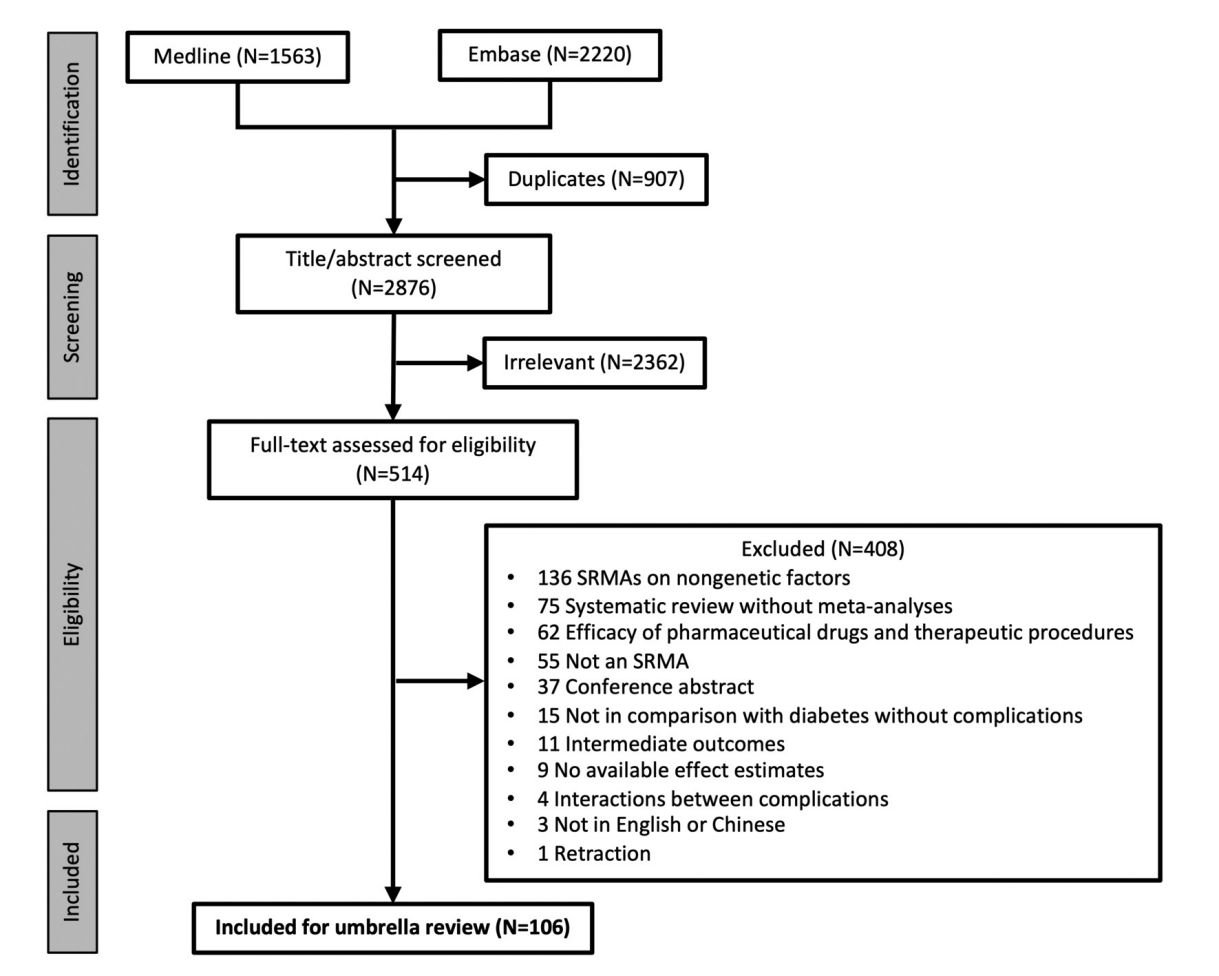
Study selection. We derived 3783 publications from the systematic literature search, following the removal of duplicates, title/abstract and full-text screening, 106 were eligible to be included in the umbrella review.

In the literature search conducted across two databases, a total of 3783 citations were initially identified. Out of these, 907 publications were identified as duplicates. Following the screening of titles and abstracts, 514 publications remained eligible for full-text review. 106 SRMAs on genetic factors associated with vascular diabetes complications were eligible for inclusion, among which 63 non-overlapping ones were finally included in the umbrella review. The process of study selection is visually presented in **Figure 1**.

### Study characteristics

The final included non-overlapping SRMAs were published between 2005 and 2022. Eleven SRMAs [19-29] included both case-control and cohort studies, two [30,31] included both case-control and post-hoc analyses of RCTs, while the other fifty [32–81] only included case-control studies. When checking the methodological quality and risk of bias using the AMSTAR 2.0 checklist, only one SRMA [38] was assessed as moderate quality with no critical flaws, 20 SRMAs [21,22,25,26,33,34,37,39,40,42,45,46,48,52,53,59,62,66,75,79] as low quality with one critical flaw (19/20 in item 2 for not registering or publishing the protocol before the conduction of SRMA), and the other 42 SRMAs [19,20,23,24,27-32,35,36,41,43,44,47,49–51,54–58,60,61,63–65,67–74,76–78,80,81] as critically low quality with more than one critical flaws (42/42 in item 2 for not registering or publishing the protocols before the conduction of SRMAs, 33/42 in item 9 for not assessing the risk of bias for individual studies, and 39/42 in item 13 for not accounting for the risk of bias of individual studies when interpreting and discussing the results of SRMAs) (Table S2 in the **Online Supplementary Document**). While these low quality SRMAs were not excluded, the credibility of evidence should be interpreted with caution using complementary evidence assessments (*i.e.* small-study effects, excess significance, Venice criteria grade and BFD*P* values).

After quality control procedures, 32 SRMAs [20,22,24–27,29,30,34,39,41,43,44,47,50,51,53–57,59,62,63,65,66,69,72,75,76,78,80] included complications only in participants with type 2 diabetes, 30 SRMAs [19,21,23,28,32,33,35–38,40,42,45,46,48,49,52,58,60,61,64,67,68,70,71,73,74,77,79,81] included participants with both type 1 and 2 diabetes, and one SRMA [31] included participants with an unspecified type of diabetes. Fifty-nine SRMAs [19–51,53,55,56,58–67,69–81] investigated biallelic genetic variants, while the other four SRMAs [52,54,57,68] examined multi-allelic variants, microsatellite variations, and co-mutations on two genes. Three SRMAs [31,40,59] did not provide sufficient citation information for their component individual studies, preventing data synthesis and evidence grading. For the remaining 60 SRMAs [19–30,32–39,41–58,60–81], both quantitative synthesis and evidence grading were conducted for the overall analyses as well as subgroup analyses stratified on type of diabetes, ethnicity, and subtypes of complications.

### Diabetic retinopathy

A total of 22 SRMAs [20,21,24,26,27,32,33,37–39,45,47,52,54,61,63–65,69,70,72,75] presented the associations between 35 genetic variants and DR. Stratification analyses on proliferative diabetic retinopathy (PDR) and non-proliferative diabetic retinopathy (NPDR) were also conducted for seven genetic variants: Ins/Del variant at *ACE* gene; SNP rs759853 at *AKR1B1* gene; SNPs rs1570360, rs2010963, rs3025039, rs699947, and rs833061 at *VEGF* gene. No highly credible associations were identified in type 1 diabetes or the mixed types of diabetes. Two associations [26,70] were graded as highly credible within the Asian population for type 2 diabetes (**Table 2**): rs1024611 at *MCP-1* gene was linked to a higher risk of DR under the dominant model (GG+GA *vs.* AA), and rs3025039 at *VEGF* gene was associated with an increased risk of PDR under the homozygous model (TT *vs*. CC). Both associations had a BFDP value less than 0.2 at the medium/low prior level (0.05 to 10^-3^), but not the very low prior level (10^-4^ to 10^-6^), indicating that these associations would not be strong enough to be detected at the genome-wide significance threshold (5 × 10^-8^) typically used in GWAS, where a hypothesis-free study design is adopted. Additionally, thirty associations [20,33,39,45,47,52,54,61,63,65,69,70,75] were categorised as credible, among which two [45,52] were in type 1 diabetes, 21 [20,33,39,45,47,52,54,61,63,65,69,70,75] were in type 2 diabetes, and seven [52,70] in the mixed types of diabetes (Table S3–5 in the **Online Supplementary Document**). Compared with the most recent GWAS on DR in type 2 diabetes in a European ancestry population [17], neither of the highly credible associations was replicated, but one credible factor (rs1800624 at *AGER* gene) demonstrated nominal significance (*P* < 0.05) (Table S6–7 in the **Online Supplementary Document**).

**Table 2:** Summary of ‘highly credible’ associations in type 1 and type 2 diabetes

Variant	A1/A2*	Outcome	Population	No. study	Sample size (cases/controls)	Genetic model†	OR (95% CIs)	*P*.effct‡	*I* ^2^	*P*.Egger‡	*P*.Sig‡	Power	Venice criteria	BFDP1§	BFDP2§	BFDP3§	BFDP4§
**Type 1 diabetes**																	
SOD2 rs4880	C/T	DPN	European	4	262/297	Allelic model	0.51 (0.4, 0.65)	5.35E-08	2.73%	0.661	0.331	1.000	AAB	5.09E-04	0.026	0.211	0.964
				4	262/297	Dominant model	0.2 (0.1, 0.39)	2.29E-06	2.72%	0.494	0.156	0.999	AAB	0.019	0.508	0.912	0.999
				4	262/297	Recessive model	0.37 (0.24, 0.56)	2.65E-06	7.32%	0.535	0.172	0.999	AAB	0.021	0.533	0.919	0.999
**Type 2 diabetes**																	
MCP-1 rs1024611	G/A	DR	Asian	4	2812/3306	Allelic model	1.21 (1.08, 1.37)	0.002	49.09%	0.051	0.375	0.985	ABC	0.508	0.982	0.998	1.000
				5	2878/3415	Dominant model	1.31 (1.13, 1.52)	3.31E-04	0.00%	0.497	0.544	0.945	AAB	0.170	0.915	0.991	1.000
				4	2812/3306	Recessive model	1.25 (1.05, 1.48)	0.013	50.65%	0.066	0.616	0.894	ACC	0.740	0.993	0.999	1.000
				4	2812/3306	Heterozygous model	1.23 (1.05, 1.45)	0.011	0.84%	0.621	1.000	0.716	BAB	0.787	0.995	0.999	1.000
				4	2812/3306	Homozygous model	1.46 (1.18, 1.81)	0.001	32.59%	0.106	0.057	0.984	ABB	0.224	0.938	0.993	1.000
VEGF rs3025039	T/C	PDR	Asian	4	256/540	Allelic model	1.89 (1.27, 2.8)	0.002	50.47%	0.129	0.795	0.998	ACB	0.396	0.972	0.997	1.000
				4	256/540	Dominant model	2 (1.17, 3.43)	0.012	59.29%	0.294	0.700	0.991	ACB	0.768	0.994	0.999	1.000
				3	212/458	Recessive model	2.93 (1.46, 5.88)	0.003	7.88%	0.566	0.382	0.876	AAB	0.497	0.981	0.998	1.000
				4	256/540	Heterozygous model	1.81 (1.04, 3.13)	0.034	57.05%	0.406	0.560	0.927	ACB	0.875	0.997	1.000	1.000
				3	212/458	Homozygous model	4.22 (2.12, 8.38)	3.93E-05	0.60%	0.732	0.651	0.977	AAB	0.028	0.604	0.939	0.999
ACACB rs2268388	T/C	Macroalbuminuria	European/Asian	8	1679/1779	Allelic model	1.76 (1.37, 2.28)	1.37E-05	71.63%	0.939	0.907	1.000	ACB	0.015	0.444	0.889	0.999
				8	1679/1779	Dominant model	1.79 (1.43, 2.25)	3.64E-07	46.93%	0.975	0.845	1.000	ABB	6.88E-04	0.035	0.266	0.973
ACE Ins/Del	Del/Ins	DKD	European/Asian/African	50	8783/6131	Allelic model	1.36 (1.24, 1.49)	1.98E-11	61.88%	0.001	0.239	1.000	ACC	8.67E-08	4.56E-06	4.56E-05	0.005
				50	8783/6131	Dominant model	1.4 (1.24, 1.58)	3.36E-08	43.62%	0.009	0.411	1.000	ABC	6.88E-05	0.004	0.035	0.784
				50	8783/6131	Recessive model	1.5 (1.31, 1.72)	6.22E-09	54.92%	0.014	0.152	1.000	ACC	1.02E-05	5.34E-04	0.005	0.348
				50	8783/6131	Codominant model	0.99 (0.9, 1.09)	0.841	39.36%	0.506	0.819	0.031	CBB	0.962	0.999	1.000	1.000
MTHFR rs1801133	T/C	DKD	Asian	13	1513/1530	Allelic model	1.52 (1.19, 1.93)	0.001	79.00%	0.296	0.058	1.000	ACB	0.232	0.941	0.994	1.000
				13	1513/1530	Dominant model	1.69 (1.17, 2.45)	0.005	80.17%	0.063	0.110	1.000	ACC	0.653	0.990	0.999	1.000
				12	1377/1430	Recessive model	1.69 (1.33, 2.14)	1.66E-05	32.55%	0.123	0.205	1.000	ABB	0.011	0.370	0.855	0.998
				13	1513/1530	Heterozygous model	1.52 (1.05, 2.2)	0.025	76.34%	0.028	0.249	0.936	ACC	0.856	0.997	1.000	1.000
				12	1377/1430	Homozygous model	2.27 (1.56, 3.29)	1.52E-05	62.24%	0.061	0.065	1.000	ACC	0.012	0.395	0.868	0.998
TCF7L2 rs7903146	T/C	DKD	European/Asian	7	1443/2300	Allelic model	1.39 (1.09, 1.76)	0.008	66.59%	0.310	0.328	1.000	ACB	0.671	0.991	0.999	1.000
				7	1443/2300	Dominant model	1.4 (1.06, 1.85)	0.017	61.14%	0.466	0.822	1.000	ACB	0.819	0.996	1.000	1.000
				7	1443/2300	Recessive model	2.18 (1.64, 2.88)	5.60E-08	2.96%	0.416	0.538	1.000	AAB	5.79E-05	0.003	0.030	0.753
				7	1443/2300	Heterozygous model	1.33 (1.06, 1.65)	0.012	35.22%	0.248	0.636	0.983	ABB	0.738	0.993	0.999	1.000
				7	1443/2300	Homozygous model	2.59 (1.78, 3.78)	6.80E-07	18.52%	0.254	0.257	1.000	AAB	8.94E-04	0.045	0.320	0.979

BFDP – Bayesian false-discovery probability, CI – confidence interval, DKD – diabetic kidney disease, DPN – diabetic peripheral neuropathy, DR – diabetic retinopathy, OR – odds ratio, PDR – proliferative diabetic retinopathy.

*A1/A2, alternative allele and reference allele for the genetic variants.

†Allelic model, A1 *vs*. A2, dominant model, A1A1 + A1A2 *vs*. A2A2, recessive model, A1A1 *vs*. A1A2+A2A2, heterozygous model, A1A2 *vs*. A2A2, homozygous model, A1A1 *vs*. A2A2, codominant model, A1A2 *vs*. A1A1 + A2A2.

‡*P.*effect, the *P*-value for meta-analyses effects; *P*.Egger, the *P*-value for small study effects; *P*.Sig, the *P*-value for excess significance.

§BFDP1, BFDP2, BFDP3, and BFDP4 were respectively calculated at the prior level of 0.05, 10^−3^, 10^−4^, and 10^−6^.

### Diabetic kidney disease

A total of 35 SRMAs [19,22,23,25,30,33–37,41,43,44,46,48,49,51,53,54,56–58,60,62,64,67,68,71,73–75,77,79–81] presented associations between 51 genetic variants and DKD. Stratification analyses for microalbuminuria, macroalbuminuria and end-stage renal disease (ESRD) were also conducted for five variants: Ins/Del variant at *ACE* gene; SNP rs2268388 at *ACACB* gene; SNP rs1799987 at *CCR5* gene; SNPs rs1800795 and rs1800796 at *IL-6* gene. Among these associations, eight [19,22,25,41,60,73] were deemed highly credible (**Table 2**), four [22,25,41,73] in type 2 diabetes, and four [19,60,73] in mixed types of diabetes. In individuals with type 2 diabetes, rs2268388 at *ACACB* gene conferred a higher risk of macroalbuminuria under the dominant model (TT + TC *vs*. CC) in the mixed population, Ins/Del variant at *ACE* gene was associated with an elevated risk of DKD under the dominant model (DD + ID *vs*. II) in the mixed population, rs1801133 at *MTHFR* gene was linked to greater risk of DKD under the recessive model (TT *vs*. TC + CC) in the Asian population, and rs7903146 at *TCF7L2* gene was connected to a higher risk of DKD under both the recessive model (TT *vs*. TC + CC) and the homozygous model (TT *vs*. CC) in the mixed population. Within the mixed types of diabetes, the association of Ins/Del variant at *ACE* gene with a higher risk of DKD under the dominant model (DD + ID *vs*. II) in the mixed population and both the dominant model (DD + ID *vs*. II) and the recessive model (DD *vs*. ID+II) in the Asian population was highly credible. Rs759853 at *AKR1B1* gene and rs1044498 at *ENPP1* gene had highly credible associations with DKD under the codominant model (TC *vs*. TT + CC) and allelic model (C *vs*. A), respectively, in the mixed population (**Table 3**). It is important to highlight that Ins/Del variant at *ACE* gene and rs7903146 at *TCF7L2* gene displayed BFD*P* values less than 0.2 at both the medium/low prior level (0.05 to 10^-3^) and the very low prior level (10^-4^ to 10^-6^), indicating their promising status as potential candidate genes for DKD. Nevertheless, it should also be noted that the effect of ACE Ins/Del might be biased by small study effects (*P*.Egger <0.1). Among the highly credible associations, four included component individual studies that violated HWE in the control groups. When these studies were excluded in sensitivity analyses, the associations of rs759853 at *AKR1B1* gene, rs1044498 at *ENPP1* gene, and rs1801133 at *MTHFR* gene were downgraded to credible while the association of rs2268388 at *ACACB* gene remained highly credible. Apart from these highly credible associations, an additional 49 associations [19,20,22,23,25,33,34,36,37,41,44,48,51,54,56,57,60,62,64,67,68,71,73,77,79,80] were considered credible, with two [19,68] in type 1 diabetes, 31 [19,22,25,30,33,34,36,37,41,44,48,51,54,56,57,62,67,77,80] in type 2 diabetes, and 16 [19,23,33,37,60,64,71,73,77,79] in the mixed types of diabetes (Table S3–5 in the **Online Supplementary Document**). In the comparison with the latest GWAS on DKD [18], none of the variants with highly credible evidence was replicated while eight variants (rs1801282 at *PPARγ* gene; rs5186 at *AGTR1* gene; rs17300539 at *ADIPOQ* gene; rs1800629 at *TNF-α* gene; rs2070744 and rs1799983 at *eNOS* gene; rs4673 at *NADPH* gene; and rs7412 defining ε2 at *APOE* gene) with credible evidence were nominally significant (*P* < 0.05) (Table S6 and Table S8 in the **Online Supplementary Document**). Although the association of rs7903146 at *TCF7L2* gene was not replicated in the latest DKD GWAS, another SNP located on *TCF7L2* gene, rs55853916, has been reported as a genome-wide significant (*P* < 5 × 10^-8^) hit for DKD [82]. Rs7903146 and rs55853916 are in linkage disequilibrium in the European population with an R^2^ of 0.7.

**Table 3:** Summary of ‘highly credible’ associations in mixed types of diabetes

Variant	A1/A2*	Outcome	Population	No. study	Sample size (cases/controls)	Genetic model†	OR (95% CIs)	*P*.effct‡	*I* ^2^	*P*.Egger‡	*P*.Sig‡	Power	Venice criteria	BFDP1§	BFDP2§	BFDP3§	BFDP4§
ACE Ins/Del	Del/Ins	DKD	European/Asian/African	62	10045/7145	Allelic model	1.32 (1.22, 1.43)	1.79E-11	59.64%	0.001	0.179	1.000	ACC	2.41E-08	1.27E-06	1.27E-05	0.001
				62	10045/7145	Dominant model	1.38 (1.24, 1.55)	8.83E-09	41.85%	0.009	0.278	1.000	ABC	7.57E-05	0.004	0.038	0.799
				62	10045/7145	Recessive model	1.41 (1.25, 1.6)	2.50E-08	52.78%	0.003	0.185	1.000	ACC	1.31E-04	0.007	0.065	0.873
				62	10045/7145	Codominant model	1.01 (0.92, 1.1)	0.902	33.93%	0.748	0.867	0.047	CBB	0.966	0.999	1.000	1.000
			Asian	41	3854/3702	Allelic model	1.51 (1.36, 1.68)	4.51E-15	52.05%	0.008	0.084	1.000	ACC	1.08E-10	5.66E-09	5.66E-08	5.66E-06
				41	3854/3702	Dominant model	1.53 (1.32, 1.77)	6.92E-09	41.76%	0.008	0.414	1.000	ABC	1.64E-05	8.61E-04	0.009	0.463
				41	3854/3702	Recessive model	1.82 (1.56, 2.12)	2.07E-14	34.37%	0.237	0.095	1.000	ABB	4.36E-11	2.29E-09	2.29E-08	2.29E-06
				41	3854/3702	Codominant model	0.95 (0.83, 1.08)	0.441	44.44%	0.657	0.798	0.188	CBB	0.953	0.999	1.000	1.000
AKR1B1 rs759853	T/C	DKD	European/Asian/Pima Indian	12	2116/2724	Allelic model	1.37 (1.17, 1.6)	1.09E-04	59.25%	0.009	0.682	1.000	ACC	0.046	0.719	0.962	1.000
				12	2116/2724	Dominant model	1.51 (1.24, 1.83)	3.46E-05	52.98%	0.003	0.662	1.000	ACC	0.020	0.520	0.916	0.999
				12	2116/2724	Recessive model	1.37 (1, 1.86)	0.049	45.01%	0.182	0.764	0.642	BBB	0.893	0.998	1.000	1.000
				12	2116/2724	Homozygous model	1.74 (1.2, 2.53)	0.004	56.30%	0.261	0.352	0.977	ACB	0.577	0.986	0.999	1.000
				12	2116/2724	Codominant model	1.31 (1.15, 1.49)	4.10E-05	3.27%	0.054	0.809	0.985	AAC	0.029	0.607	0.939	0.999
ENPP1 rs1044498	C/A	DKD	European/Asian/African	7	1605/1963	Allelic model	1.35 (1.15, 1.58)	2.79E-04	40.99%	0.408	0.382	0.999	ABB	0.101	0.856	0.983	1.000
				7	1605/1963	Dominant model	1.4 (1.15, 1.71)	0.001	43.68%	0.595	0.614	0.996	ABB	0.315	0.960	0.996	1.000
				7	1605/1963	Recessive model	1.53 (1.12, 2.09)	0.007	5.45%	0.435	0.770	0.797	BAB	0.702	0.992	0.999	1.000
				7	1605/1963	Homozygous model	1.72 (1.23, 2.4)	0.002	10.50%	0.081	0.568	0.927	AAC	0.385	0.971	0.997	1.000
EPO rs1617640	T/G	PDR and ESRD	European	3	1618/954	Allelic model	1.48 (1.32, 1.67)	5.56E-11	3.14%	0.482	0.727	1.000	AAB	3.90E-07	2.05E-05	2.05E-04	0.020
				3	1618/954	Dominant model	1.59 (1.26, 2.02)	1.24E-04	13.78%	0.268	0.966	0.989	AAB	0.084	0.829	0.980	1.000
				3	1618/954	Recessive model	1.79 (1.5, 2.14)	9.88E-11	0.02%	0.852	0.674	1.000	AAB	3.29E-07	1.73E-05	1.73E-04	0.017
				3	1618/954	Heterozygous model	1.3 (1.02, 1.67)	0.036	13.19%	0.271	0.635	0.649	BAB	0.888	0.998	1.000	1.000
				3	1618/954	Homozygous model	2.2 (1.7, 2.84)	2.12E-09	6.97%	0.367	0.653	1.000	AAB	2.50E-06	1.32E-04	0.001	0.116

BFDP – Bayesian false-discovery probability, CI – confidence interval, DKD – diabetic kidney disease, ESRD – end-stage renal disease, OR – odds ratio, PDR – proliferative diabetic retinopathy

*A1/A2, alternative allele and reference allele for the genetic variants.

†Allelic model, A1 *vs*. A2, dominant model, A1A1 + A1A2 *vs*. A2A2, recessive model, A1A1 *vs*. A1A2 + A2A2, heterozygous model, A1A2 *vs*. A2A2, homozygous model, A1A1 *vs*. A2A2, codominant model, A1A2 *vs*. A1A1 + A2A2.

‡*P.*effect, the *P*-value for meta-analyses effects; *P*.Egger, the *P*-value for small study effects; *P*.Sig, the *P*-value for excess significance.

§BFDP1, BFDP2, BFDP3, and BFDP4 were respectively calculated at the prior level of 0.05, 10^−3^, 10^−4^, and 10^−6^.

### Diabetic neuropathy and foot disease

Four SRMAs [28,64,66,75] reported the association between four variants and diabetic peripheral neuropathy (DPN). The association between rs4880 at *SOD2* gene and DPN in type 1 diabetes in the European population was regarded as highly credible under the allelic model (C *vs*. T), dominant model (CC+CT *vs*. TT), and recessive model (CC vs CT+TT) (**Table 2**) [64]. Nevertheless, the control groups of all the individual studies included in this association violated the HWE. No association was regarded as credible. Only one SRMA [78] assessed the effects of two variants on diabetic foot disease, and neither of them was statistically significant.

### Macrovascular complications and combined endpoints

Five SRMAs [33,37,44,54,55] involving eight variants focused on macrovascular complications. Seven SRMAs [21,29,61,64,75–77] involving nine variants investigated the combined endpoint with all types of microvascular complications, while three SRMAs [42,50,54] involving five variants examined the combined endpoint with all vascular complications. When combining PDR with ESRD within the mixed types of diabetes in the European population, the effect of rs1617640 at *EPO* gene was regarded as highly credible under the allelic model (T *vs*. G), dominant model (TT + TG *vs*. GG), recessive model (TT *vs*. TG + GG), and homozygous model (TT *vs*. GG) (**Table 2**) [21]. The BFD*P* values were below 0.2 at both the medium/low (0.05 to 10^-3^) and the very low prior level (10^-4^ to 10^-6^), indicating that *EPO* is a strong candidate gene for microvascular complications.

## DISCUSSION

Our umbrella review summarised published evidence on the genetic basis of vascular diabetes complications and provides a robust and significant synthesis of the evidence with re-analyses to generate more precise interpretations. Overall, we identified 12 highly credible associations involving ten distinct candidate genes, which could provide insights into the biological mechanisms underlying vascular diabetes complications.

Genes *MCP-1* and *VEGF* were identified as candidate genes for DR. Gene *MCP-1* encodes monocyte chemoattractant protein-1 (MCP-1). The expression of MCP-1 is regulated by the transcription factor nuclear factor-kappa B (NF-κB). In people with diabetes, retinal pigmented epithelial cells and Muller cells are involved in the production of MCP-1 in the vitreous fluid, which in turn contributes to retinal inflammation, vascular permeability and neovascularisation. MCP-1 also recruits and activates monocytes and macrophages into the retina, and further produces reactive oxygen species (ROS), causes cell injury and angiogenesis and contributes to the pathogenesis of DR [83]. Gene *VEGF* is the protein-coding gene for vascular endothelial growth factor (VEGF). The expression of VEGF is induced by hypoxia, retinal pigmented epithelium, endothelial cells, pericytes, glial cells and ganglion cells, which are involved in the production of VEGF in the eye. The concentration of intraocular VEGF has been found to be temporally associated with the development and progression of iris neovascularisation. VEGF also acts as a stimulation of retinal leakage after binding with its receptors, which consequently caused macula oedema and visual impairment in people with diabetes [84]. Intravitreal injection of VEGF inhibitors ranibizumab and aflibercept has been approved for the treatments for all subtypes of DR, offering a safer option than conventional laser therapy [84].

This umbrella review discovered several candidate genes for DKD, highlighting the effects of glucose metabolism, fatty acid metabolism, and one-carbon metabolism. *AKR1B1* gene encodes the rate-limiting enzyme aldose reductase (AR) that is involved in the reduction of glucose to sorbitol [85]. *ENPP1* gene is a member of the ecto-nucleotide pyrophosphatase/phosphodiesterase (ENPP) family, encoding a type II transmembrane glycoprotein [86]. The overexpression of *ENPP1* gene has a deleterious effect on the function of the insulin receptor, inhibits subsequent signalling by decreasing the autophosphorylation of its β-subunit, and thus works as a gatekeeper of insulin action [86]. *ACACB* gene encodes acetyl-CoA carboxylase (ACC)-beta, which is thought to regulate the rate-limiting step in fatty acid uptake and oxidation by mitochondria [87]. ACC has gained great attention for its potential as a drug target for multiple metabolic diseases including metabolic syndrome, obesity, and diabetes [88]. *MTHFR* gene participates in one-carbon metabolism, it encodes an enzyme that catalyses the conversion of 5,10-methylenetetrahydrofolate to 5-methyltetrahydrofolate, acting as a co-substrate for the remethylation of homocysteine to methionine [89]. Increased level of circulating homocysteine has been shown to be a risk factor for DKD by both observational studies and Mendelian randomisation studies [90].

Additionally, this umbrella review demonstrated highly credible associations of DKD with the Ins/Del variant at *ACE* gene and rs7903146 at *TCF7L2* gene. *ACE* gene is a pivotal factor of the renin-angiotensin-aldosterone system (RAAS) containing 26 exons and 25 introns [73]. The 287 base pair Alu Ins/Del in the 16th intron is strongly associated with the circulating level of angiotensin-converting enzyme (ACE) [73]. ACE is involved in the conversion of low activity angiotensin I to high activity angiotensin II, which regulates renal arterial blood pressure [73]. ACE inhibitors and angiotensin receptor blockers (ARBs) are recommended as the first-line medications for the treatment of hypertension [91,92]. Treatment with ACE inhibitors is also recommended for the management of DKD [93]. The *TCF7L2* locus is one of the strongest signals for type 2 diabetes and is associated with latent autoimmune diabetes in adults (LADA) [94]. Although it is not associated with type 1 diabetes overall, the *TCF7L2* locus independently regulates the expression of a single islet autoantibody and influences the progression of islet autoimmunity from single to multiple autoantibody positivity in individuals with type 1 diabetes [95,96]. Rs7903146, situated within the fourth intron of the *TCF7L2* gene, is the only variant that captures the association with type 2 diabetes across different ethnic groups [94]. *TCF7L2* gene encodes the essential transcription factor TCF7L2 in the Wnt/β-catenin signalling pathway. The activation of Wnt/β-catenin plays a key role in driving podocyte and tubular injury in the development of DKD [97]. Moreover, TCF7L2 exhibits multiple anti-atherosclerotic effects through the regulation of metabolic homeostasis, macrophage polarisation, and neointimal hyperplasia [98]. Taken together, TCF7L2 reveals great potential as a drug target for the management of microvascular and macrovascular complications of diabetes.

A highly credible association between *SOD2* gene and DPN was also detected. *SOD2* gene encodes superoxide dismutase 2 (SOD2), also known as manganese superoxide dismutase (MnSOD), which is a key enzyme combating oxidative stress within cells. Oxidative stress is considered to be a crucial pathophysiological pathway to DPN [99]. Overexpression of *SOD2* gene has shown to be protective against the formation of ROS and subsequent cleavage of caspase-3 in dorsal root ganglion (DRG) neurons and thus blocked glucose-mediated injury, while the decreased expression of *SOD2* gene would increase the oxidative stress and cause the morphological and physiological manifestations of DPN. A few antioxidant supplements, such as taurine, N-Acetyl cysteine and Zinc, have been considered to manage DPN via increasing serum levels of SOD2 [99].

Finally, a highly credible association between rs1617640 at *EPO* gene and the combined PDR and ESRD endpoint was detected. *EPO* gene encodes a hormone erythropoietin to regulate haematopoiesis. The synthetic form of erythropoietin is used to treat anaemia that results from chronic kidney disease [100]. In addition, erythropoietin also has nonhematopoietic features including angiogenic, anti-inflammatory, and antioxidant effects [101]. Although VEGF is a primary mediator of retinal angiogenesis, erythropoietin exhibits its independent angiogenic effect in the development of PDR [102], indicating its potential as a therapeutic target.

Our umbrella review highlighted ten candidate genes with highly credible evidence. None of these highly credible associations reached genome-wide significance (*P* < 5 × 10^-8^) in the latest GWASs for DR and DKD [17,18], but nine credible associations were nominally significant (*P* < 0.05) in the GWASs, and rs55853916 at *TCF7L2* gene is in high linkage disequilibrium with rs7903146, which has been reported as a genome-wide significant hit (*P* < 5 × 10^-8^) for DKD [82]. The low concordance between findings from SRMAs and GWASs can be attributed to the different scope of each approach and their limitations. First, the hypotheses underlying SRMAs and GWASs differ significantly. The individual studies included in the SRMAs were candidate gene studies: only specific genetic variants (*e.g.* variant X on gene *Y*) were genotyped and tested for association with disease under predefined hypotheses in a small number of participants. While GWASs adopt a hypothesis-free setting, every genetic variant across the entire genome is genotyped and tested for the association and a more stringent *P*-value (*i.e.* 5 × 10^-8^) is employed to account for multiple testing. Second, different genetic models were tested in the SRMAs and GWASs. Most of the highly credible evidence in this umbrella review came from the dominant and recessive models, whereas GWASs typically rely on the allelic model. These genetic models make different assumptions on how the alleles for a given variant contribute to the phenotype. Take the alternative allele A1 and reference allele A2 and their contribution to phenotype risk k as an example, the allelic model assumes that if the phenotype risk is k for the heterozygote A1A2, then the phenotype risk is 2k for the homozygote A1A1. However, the dominant model compares A1A1+A1A2 *vs*. A2A2, assuming that having one or more copies of A1 (heterozygote A1A2 or homozygote A1A1) confers the same risk k, and the recessive model compares A1A1 *vs*. A1A2+A2A2, assuming that only having two copies of A1 (homozygote A1A1) carries an increased risk. Therefore, the conclusions derived from different models can be significantly distinct [103]. While the allelic model provides the most sufficient statistical power in general, it may fail to detect associations if the true genetic effect follows a dominant or recessive model. Given the highly credible evidence from dominant and recessive models in the SRMAs of candidate gene studies, it would be beneficial to include these models in GWAS analyses of vascular diabetes complications to enhance the reliability and biological relevance of GWAS findings. Nevertheless, it is important to acknowledge that both SRMAs and GWASs have their own limitations. The highly credible findings came from 10 SRMAs that were rated as low (4/10) and critically low (6/10) quality because they either did not assess the risk of bias for individual studies, did not account for the risk of bias when interpreting and discussing results from their meta-analyses, or did not quantitively assess publication bias. These limitations could lead to an inflated type 1 error and the presence of false positive findings. The sample size of the GWASs of vascular diabetes complications was small, thus limiting the statistical power to accurately detect susceptibility loci, especially given the complex genetic architecture of the studied phenotypes. This was evident by the very few GWAS hits identified by these studies. Other differences between SRMAs and GWASs that might contribute to the low concordance of findings include differences in phenotype definitions, ethnicities, and the combination of two genetically different types of diabetes. The individual studies included in the SRMAs roughly defined DR from ophthalmological assessment instead of grading with the Early Treatment Diabetic Retinopathy Study (ETDRS) score. The SRMAs defined DKD solely based on urine albumin excretion (AER), estimated Glomerular Filtration Rate (eGFR) or urinary albumin/creatinine ratio (ACR), whereas the GWASs defined DKD using combinations of different thresholds on these quantitative measures of kidney function. The SRMAs usually included populations of multiple ethnicities without adjusting for possible population stratification, while the GWASs were restricted to European ancestry individuals. We incorporated ethnic-specific meta-analyses and assessed their evidence respectively when such analyses were performed in the original SRMAs. However, we were unable to conduct ethnic-specific analyses across all genetic factors and complications when the original SRMAs did not include such analyses or lacked sufficient raw data. Similarly, while the GWAS of DR focused solely on type 2 diabetes, the GWAS of DKD and half of the SRMAs involved mixed samples of type 1 diabetes and type 2 diabetes. Given different factors affecting the genetic susceptibility to type 1 diabetes and type 2 diabetes, including both types of diabetes in the meta-analyses for vascular complications could introduce heterogeneity, thereby affecting conclusions. Subgroup analyses for type 1 and type 2 diabetes were conducted when relevant data was available, but this was not possible if the original SRMAs did not perform such analyses or provide adequate raw data.

Despite these limitations, this umbrella review thoroughly examined existing evidence on genetic factors for a wide range of vascular diabetes complications. We meticulously assessed the methodological quality and risk of bias for each included SRMA, implemented rigorous quality control steps, re-analysed the original data from individual studies, and compared it with the evidence from GWASs. By consolidating and rigorously evaluating earlier genetic evidence on vascular diabetes complications, this umbrella review provided valuable supplementary information to complement the findings of large-scale GWASs.

## CONCLUSIONS

This umbrella review summarised published evidence on the genetics of vascular diabetes complications and highlighted ten candidate genes involved in nutrient metabolism, inflammation, oxidative stress, angiogenesis, and nuclear transduction. Two of these candidate genes are targets for drugs currently used to treat diabetic complications (*VEGF* for DR and *ACE* for DKD); three of these candidate genes have additional evidence supporting them as possible drug targets (*ACACB*, *TCF7L2*, and *EPO*). This umbrella review complemented the findings from the GWASs of vascular diabetes complications and shed light on the optimal selection of genetic models for the design of GWASs. Further investigation via mechanistic studies, or bioinformatic studies that incorporate genetics of gene expression or protein levels, is warranted to establish the precise role of these candidate genes in the pathology of vascular diabetes complications and their utility as potential drug targets.

## Additional material


Online Supplementary Document
