# Supplementary material for: Associations of genetic factors with vascular diabetes complications: an umbrella review
Source: J Glob Health. 2025 Mar 21;15:04081. doi: 10.7189/jogh.15.04081 (PMC11927039; doi:10.7189/jogh.15.04081)
Supplement: Online Supplementary Document [file jogh-15-04081-s001.pdf]

Supplement to: Zhou X, Yang N, Xu W, Li X, Spiliopoulou A, Theodoratou E. Associations of genetic factors with vascular diabetes complications: an umbrella review. J Glob Health. 2025;15:04081.

Table S1. Search strategy.
Table S2. Characteristics and methodological assessment of included SRMAs.
Table S3. Summary of "credible" and "not credible" associations in type 1 diabetes.
Table S4. Summary of "credible" and "not credible" associations in type 2 diabetes.
Table S5. Summary of "credible" and "not credible" associations in mixed types of diabetes.
Table S6. Characteristics of the latest GWASs for DR and DKD.
Table S7. Summary of GWAS estimates for "highly credible" and "credible" associations in DR.
Table S8. Summary of GWAS estimates for "highly credible" and "credible" associations in DKD.

Supplementary Table 1. Search strategy.

**Search strategy for systematic review and meta-analyses of observational studies and randomized clinical trials**	
1	exp diabetes complications/
2	(diabet* adj3 (coma or ketoacidosis or (cardiomyopath* or cardiovascular disease* or CVD) or cerebrovascular disease* or peripheral vascular disease* or (nephropath* or kidney disease* or renal disease*) or (neuropath* or polyneuropath*) or retinopath* or angiopath* or (foot ulcer* or pain or amputation* or periodontal disease* or infection* or depress* or distress or anxiety or eating disorder* or ((pregnanc* or obstetric*) adj2 (outcome* or complicat* or disorder*))))).mp.
3	1 or 2
4	exp Systematic Review/
5	exp Meta-Analysis/
6	(systematic$ adj2 (review$ or overview)).ti,ab.
7	(meta?anal$ or meta anal$ or meta-anal$ or metaanal$ or metanal$).ti,ab.
8	4 or 5 or 6 or 7
9	biomarker*.mp.
10	risk factor*.mp.
11	molecular pathway*.mp.
12	9 or 10 or 11
13	3 and 8 and 12
**Search strategy for Mendelian randomization analyses**	
1	exp diabetes complications/
2	(diabet* adj3 (coma or ketoacidosis or (cardiomyopath* or cardiovascular disease* or CVD) or cerebrovascular disease* or peripheral vascular disease* or (nephropath* or kidney disease* or renal disease*) or (neuropath* or polyneuropath*) or retinopath* or angiopath* or (foot ulcer* or pain or amputation* or periodontal disease* or infection* or depress* or distress or anxiety or eating disorder* or ((pregnanc* or obstetric*) adj2 (outcome* or complicat* or disorder*))))).mp.
3	1 or 2
4	mendelian randomi?ation.ti,ab.
5	3 and 4

Supplementary Table 2. Characteristics and methodological assessment of included SRMAs.

Author-Year	Title	Type of DM	Item 1	Item 2	Item 3	Item 4	Item 5	Item 6	Item 7	Item 8	Item 9	Item 10	Item 11	Item 12	Item 13	Item 14	Item 15	Item 16	Quality
Abhary-2009	A systematic meta-analysis of genetic association studies for diabetic retinopathy	1/2	Yes	No	No	Yes	No	Yes	No	Partial Yes	No	No	Yes	No	No	No	Yes	Yes	Critically low
Chen-2021	The susceptibility of SERPINE1 rs1799889 SNP in diabetic vascular complications: a meta-analysis of fifty-one case-control studies	1/2	Yes	No	No	Yes	Yes	Yes	Partial Yes	Yes	Yes	No	Yes	Yes	Yes	No	Yes	Yes	Low
Cui-2015	Is rs759853 polymorphism in promoter of aldose reductase gene a risk factor for diabetic nephropathy? A meta-analysis	1/2	Yes	No	No	Yes	No	Yes	Partial Yes	Yes	No	No	Yes	No	No	No	Yes	Yes	Critically low
Cui-2019	Association of Interleukin-6 -174G/C Polymorphism with the Risk of Diabetic Nephropathy in Type 2 Diabetes: A Meta-analysis	2	Yes	No	No	Yes	No	Yes	Partial Yes	Yes	Yes	No	Yes	Yes	Yes	No	Yes	Yes	Low
Dieter-2020	866G/A and INS/DEL polymorphisms in the UCP2 gene and diabetic kidney disease: Case-control study and meta-analysis	1/2	Yes	No	No	Yes	Yes	Yes	Partial Yes	No	Yes	No	Yes	Yes	Yes	Yes	No	Yes	Critically low
Ding-2012	Association between two genetic polymorphisms of the renin-angiotensin-aldosterone system and diabetic nephropathy: a meta-analysis	1/2	Yes	No	No	No	No	Yes	Partial Yes	Partial Yes	No	No	Yes	No	No	Yes	Yes	No	Critically low
Ding-2015-1	Peroxisome proliferator-activated receptor gamma pro12Ala polymorphism decrease the risk of diabetic nephropathy in type 2 diabetes: A meta analysis	2	Yes	No	No	Yes	No	Yes	No	Partial Yes	No	No	Yes	No	No	No	Yes	Yes	Critically low
Ding-2015-2	Association between transcription factor 7-like 2 rs7903146 polymorphism and diabetic retinopathy in type 2 diabetes mellitus: A meta-analysis	2	Yes	No	No	Yes	Yes	Yes	Partial Yes	Yes	No	No	Yes	No	No	Yes	Yes	Yes	Critically low
Dong-2018	The roles of endothelial nitric oxide synthase gene polymorphisms in diabetes mellitus and its associated vascular complications: a systematic review and meta-analysis	1/2	Yes	No	No	Yes	No	Yes	Partial Yes	Yes	Yes	No	Yes	Yes	Yes	No	Yes	Yes	Low
Gao-2021	Association of Tumor Necrosis Factor-Alpha-308 G/A and -238 G/A Polymorphism with Diabetic Retinopathy: A Systematic Review and Updated Meta-Analysis	1/2	Yes	Yes	No	Yes	Yes	Yes	Partial Yes	Yes	Yes	No	Yes	Yes	Yes	Yes	Yes	Yes	Moderate
Garcia-Hernandez-2019	The A1298C methylenetetrahydrofolate reductase polymorphism augments the risk of developing of diabetic retinopathy: A meta-analysis	2	Yes	No	No	Yes	Yes	Yes	Partial Yes	Yes	Yes	No	Yes	Yes	Yes	No	Yes	Yes	Low
Gong-2015	Association of platelet glycoprotein receptor alpha2beta1 integrin and glycoprotein IIIa gene polymorphisms with diabetic retinopathy: evidence from 3007 subjects	1/2	Yes	No	No	Yes	No	Yes	Partial Yes	Partial Yes	Yes	No	Yes	Yes	Yes	No	Yes	Yes	Low
Guan-2020	Methylenetetrahydrofolate reductase genetic polymorphism and the risk of diabetic nephropathy in type 2 diabetic patients	2	Yes	No	No	Yes	No	Yes	Partial Yes	Yes	No	No	Yes	No	No	Yes	Yes	Yes	Critically low
Harun-Or-Roshid-2022	Association of hypoxia inducible factor 1-Alpha gene polymorphisms with multiple disease risks: A comprehensive meta-analysis	1/2	Yes	No	No	Yes	No	No	Partial Yes	Yes	Yes	No	Yes	Yes	Yes	Yes	Yes	Yes	Low
Kang-2012	Association of RAGE gene polymorphisms with type 2 diabetes mellitus, diabetic retinopathy and diabetic nephropathy	2	Yes	No	No	Yes	Yes	Yes	No	Yes	No	No	Yes	No	No	Yes	Yes	Yes	Critically low
Li-2014	The association between lipid metabolism gene polymorphisms and nephropathy in type 2 diabetes: a meta-analysis	2	Yes	No	No	Yes	No	Yes	Partial Yes	Yes	Yes	No	Yes	Yes	Yes	Yes	Yes	Yes	Low
Li-2015	Association of the NAD(P)H oxidase p22 phox gene C242T polymorphism with type 2 diabetes mellitus, diabetic nephropathy, and carotid atherosclerosis with type 2 diabetes mellitus: A meta-analysis	2	Yes	No	No	Yes	No	Yes	Partial Yes	Yes	No	No	Yes	No	No	Yes	Yes	Yes	Critically low
Li-2017	Associations between erythropoietin polymorphisms and risk of diabetic microvascular complications	1/2	Yes	No	No	Yes	Yes	Yes	Partial Yes	Yes	Yes	No	Yes	Yes	Yes	No	Yes	Yes	Low
Lin-2014	Adiponectin gene polymorphisms and susceptibility to diabetic nephropathy: a meta-analysis	1/2	Yes	No	No	Yes	Yes	Yes	Partial Yes	Yes	No	No	Yes	No	No	No	Yes	Yes	Critically low

Lin-2020	Association between Aldose Reductase Gene C(-106)T Polymorphism and Diabetic Retinopathy: A Systematic Review and Meta-Analysis	1/2	Yes	No	No	Yes	Yes	Yes	Partial Yes	Yes	Yes	No	Yes	Yes	Yes	Yes	Yes	Yes	Low
Liu-2014	TGF-beta1 gene polymorphism in association with diabetic retinopathy susceptibility: A systematic review and meta-analysis	2	Yes	No	No	Yes	Yes	Yes	Partial Yes	Yes	No	No	Yes	No	No	No	Yes	Yes	Critically low
Liu-2018	Association between E469K polymorphism in the ICAM1 gene and the risk of diabetic nephropathy: A meta-analysis	1/2	Yes	No	No	Yes	Yes	Yes	Partial Yes	Yes	Yes	No	Yes	Yes	Yes	Yes	Yes	Yes	Low
Liu-2019	Association between angiotensinogen T174M polymorphism and the risk of diabetic nephropathy: A meta-analysis	1/2	Yes	No	No	Yes	No	Yes	Partial Yes	Yes	No	No	Yes	No	No	No	Yes	Yes	Critically low
Liu-2020	Effects of TNF-alpha-308G/A Polymorphism on the Risk of Diabetic Nephropathy and Diabetic Retinopathy: An Updated Meta-Analysis	1/2	Yes	No	No	Yes	No	Yes	Partial Yes	Yes	Yes	No	Yes	Yes	Yes	No	Yes	Yes	Low
Lu-2010	The -374A allele of the RAGE gene as a potential protective factor for vascular complications in type 2 diabetes: a meta-analysis	2	Yes	No	No	Yes	No	Yes	Partial Yes	Yes	No	No	Yes	No	No	No	Yes	Yes	Critically low
Ma-2012	Meta-analysis of association between the Pro12Ala polymorphism of the peroxisome proliferator-activated receptor-gamma2 gene and diabetic retinopathy in Caucasians and Asians	2	Yes	No	No	Yes	Yes	Yes	Partial Yes	Yes	No	No	Yes	No	No	Yes	Yes	Yes	Critically low
Mao-2014	Monocyte chemoattractant protein-1-2518G/A gene polymorphism and the risk of nephropathy in type 2 diabetes mellitus among Asians: A meta-analysis	2	Yes	No	No	Yes	No	Yes	Partial Yes	Yes	No	No	Yes	No	No	Yes	Yes	Yes	Critically low
Mi-2019	Meta-analysis of the association between aldose reductase gene (CA)n microsatellite variants and risk of diabetic retinopathy	1/2	Yes	No	No	Yes	No	Yes	Partial Yes	Yes	Yes	No	Yes	Yes	Yes	Yes	Yes	Yes	Low
Naing-2018	An association between IL-10 promoter polymorphisms and diabetic nephropathy: a meta-analysis of case-control studies	2	Yes	No	No	Yes	Yes	Yes	Partial Yes	Yes	Yes	No	Yes	Yes	Yes	No	Yes	Yes	Low
Nath-2019	The GSTM1 and GSTT1 Null Genotypes Increase the Risk for Type 2 Diabetes Mellitus and the Subsequent Development of Diabetic Complications: A Meta-analysis	2	Yes	No	No	Yes	Yes	Yes	Partial Yes	Yes	No	No	Yes	No	No	Yes	Yes	Yes	Critically low
Ning-2022	Association of the Transcription Factor 7-Like 2 (TCF7L2) rs7903146 Polymorphism with the Risk of Diabetic Nephropathy: A Meta-Analysis	2	Yes	No	No	Yes	No	Yes	Partial Yes	Yes	Yes	No	Yes	Yes	Yes	Yes	Yes	Yes	Low
Peng-2013	Association of four genetic polymorphisms of AGER and its circulating forms with coronary artery disease: a meta-analysis	2	Yes	No	No	Yes	Yes	Yes	Partial Yes	Partial Yes	No	No	Yes	No	No	Yes	Yes	Yes	Critically low
Shen-2019	I/D polymorphism of ACE and risk of diabetes-related end-stage renal disease: a systematic review and meta-analysis	2	Yes	No	No	Yes	Yes	Yes	Partial Yes	Yes	No	No	Yes	Yes	Yes	No	Yes	Yes	Critically low
Shi-2015	Association between the RAGE gene -374T/A, -429T/C polymorphisms and diabetic nephropathy: A meta-analysis	1/2	Yes	No	No	Yes	No	Yes	Partial Yes	Yes	No	No	Yes	No	No	No	Yes	Yes	Critically low
Shi-2020	ε2 allele and ε2-involved genotypes (ε2/ε2, ε2/ε3, and ε2/ε4) may confer the association of APOE genetic polymorphism with risks of nephropathy in type 2 diabetes: a meta-analysis	2	Yes	No	No	Yes	No	Yes	Partial Yes	Yes	Yes	No	Yes	No	No	Yes	Yes	Yes	Critically low
Shu-2018	The Roles of IL-10 Gene Polymorphisms in Diabetes Mellitus and Their Associated Complications: A Meta-Analysis	2	Yes	No	No	Yes	No	Yes	Partial Yes	No	Yes	No	Yes	Yes	Yes	No	Yes	Yes	Low
Sortica-2015	Association between the ENPP1 K121Q polymorphism and risk of diabetic kidney disease: a systematic review and meta-analysis	1/2	Yes	No	No	Yes	Yes	Yes	Partial Yes	No	Yes	No	Yes	Yes	Yes	No	No	Yes	Critically low
Su-2013	Intracellular Adhesion Molecule-1 K469E Gene Polymorphism and Risk of Diabetic Microvascular Complications: A Meta-Analysis	1/2	Yes	No	No	Yes	Yes	Yes	Partial Yes	Yes	No	No	Yes	No	No	Yes	Yes	Yes	Critically low
Sun-2017	Association between interleukin-6 -174G>C and -634C>G polymorphism and diabetic nephropathy: A meta-analysis	2	Yes	No	No	Yes	No	Yes	Partial Yes	Yes	Yes	No	Yes	Yes	Yes	No	Yes	Yes	Low
Tao-2017	Association between the RAGE (receptor for advanced glycation end-products)-374T/A gene polymorphism and diabetic retinopathy in T2DM	2	Yes	No	No	Yes	No	Yes	Partial Yes	Partial Yes	No	No	Yes	Yes	No	Yes	Yes	Yes	Critically low

Tian-2011	Association of the C47T polymorphism in SOD2 with diabetes mellitus and diabetic microvascular complications: a meta-analysis	1/2	Yes	No	No	Yes	Yes	Yes	Partial Yes	Yes	No	No	Yes	No	No	Yes	Yes	Yes	Critically low
Ulhaq-2020	The role of IL-6-174 G/C polymorphism and intraocular IL-6 levels in the pathogenesis of ocular diseases: a systematic review and meta-analysis	2	Yes	No	No	Yes	Yes	Yes	Partial Yes	Yes	Yes	No	Yes	No	No	Yes	Yes	Yes	Critically low
Vardi-2012	Haptoglobin genotype and cardiovascular outcomes in diabetes mellitus - natural history of the disease and the effect of vitamin E treatment. Meta-analysis of the medical literature	Unspecified	Yes	No	Yes	Yes	Yes	Yes	Partial Yes	Partial Yes	Yes	No	Yes	Yes	No	No	Yes	Yes	Critically low
Wang-2016	Association of monocyte chemoattractant protein-1 gene 2518A/G polymorphism with diabetic retinopathy in type 2 diabetes mellitus: A meta-analysis	2	Yes	Yes	No	Yes	Yes	Yes	Partial Yes	Yes	Yes	No	Yes	Yes	Yes	No	No	Yes	Low
Wu-2017-1	The Association of Haptoglobin Gene Variants and Retinopathy in Type 2 Diabetic Patients: A Meta-Analysis	2	Yes	No	No	Yes	Yes	Yes	Partial Yes	Yes	Yes	No	Yes	Yes	No	No	Yes	Yes	Critically low
Wu-2017-2	Effects of Common Polymorphisms in the MTHFR and ACE Genes on Diabetic Peripheral Neuropathy Progression: a Meta-Analysis	2	Yes	No	No	Yes	No	Yes	Partial Yes	Partial Yes	Yes	No	Yes	Yes	Yes	No	Yes	Yes	Low
Xu-2008	Association between (AC)n dinucleotide repeat polymorphism at the 5'-end of the aldose reductase gene and diabetic nephropathy: A meta-analysis	1/2	Yes	No	No	Yes	No	Yes	Partial Yes	Yes	No	No	Yes	No	No	No	Yes	Yes	Critically low
Xu-2015	Angiotensin-converting enzyme I/D polymorphism is a genetic biomarker of diabetic peripheral neuropathy: Evidence from a meta-analysis	1/2	Yes	No	No	Yes	No	Yes	Partial Yes	Yes	No	No	Yes	No	No	No	Yes	Yes	Critically low
Xu-2016	Association of aldosterone synthase (CYP11B2) -344 T/C polymorphism with diabetic nephropathy: A meta-analysis	1/2	Yes	No	No	Yes	No	Yes	Partial Yes	Yes	No	No	Yes	No	No	Yes	Yes	Yes	Critically low
Xu-2020	Methylenetetrahydrofolate reductase C677T polymorphism and diabetic retinopathy risk: a meta-analysis of the Chinese population	2	Yes	No	No	Yes	Yes	Yes	Partial Yes	Yes	No	No	Yes	No	No	No	No	Yes	Critically low
Yang-2020	Association of VEGF Gene Polymorphisms with Susceptibility to Diabetic Retinopathy: A Systematic Review and Meta-Analysis	1/2	Yes	No	No	Yes	No	Yes	Partial Yes	Partial Yes	No	No	Yes	No	No	No	Yes	Yes	Critically low
Yin-2018	Association between the vitamin D receptor gene polymorphisms and diabetic nephropathy risk: A meta-analysis	1/2	Yes	No	No	Yes	Yes	Yes	Partial Yes	Yes	Yes	No	Yes	No	No	No	Yes	Yes	Critically low
Yu-2016	Association of genetic variants in the receptor for advanced glycation end products gene with diabetic retinopathy: A meta-analysis	2	Yes	No	No	Yes	Yes	Yes	Partial Yes	Partial Yes	No	No	Yes	No	No	No	Yes	Yes	Critically low
Zeng-2021	The impact of angiotensin converting enzyme insertion/deletion gene polymorphism on diabetic kidney disease: A debatable issue	1/2	Yes	No	No	Yes	No	No	Partial Yes	Yes	Yes	No	Yes	No	No	No	Yes	Yes	Critically low
Zhang-2014	A meta-analysis of the association of G915C, G800A, C509T gene polymorphism of transforming growth factor-beta1 with diabetic nephropathy risk	1/2	Yes	No	No	Yes	No	Yes	Partial Yes	Yes	No	No	Yes	No	No	No	No	Yes	Critically low
Zhang-2015	Matrix metalloproteinase 9 gene promoter (Rs 3918242) mutation reduces the risk of diabetic microvascular complications	2	Yes	No	No	Yes	Yes	Yes	Partial Yes	Yes	No	No	Yes	No	No	Yes	Yes	Yes	Critically low
Zhang-2016	Association of chemokine ligand 5/chemokine receptor 5 gene promoter polymorphisms with diabetic microvascular complications: A meta-analysis	1/2	Yes	No	No	Yes	Yes	Yes	Partial Yes	No	No	No	Yes	No	No	Yes	Yes	Yes	Critically low
Zhang-2017	Endothelial nitric oxide synthase 27VNTR (4b/4a) gene polymorphism and the risk of diabetic microvascular complications in Chinese populations	2	Yes	No	No	Yes	No	Yes	Partial Yes	Yes	No	No	Yes	No	No	Yes	Yes	Yes	Critically low
Zhang-2022	Toll like receptor 4 gene Asp299Gly polymorphism increases the risk of diabetic microvascular complications: a meta analysis	2	Yes	No	No	Yes	No	Yes	Partial Yes	Yes	Yes	No	Yes	Yes	Yes	Yes	Yes	Yes	Low
Zhao-2020	Genetic Polymorphisms and the Risk of Diabetic Foot: A Systematic Review and Meta-Analyses	2	Yes	No	No	Yes	No	No	Partial Yes	Yes	Yes	No	Yes	No	No	No	Yes	Yes	Critically low

Zhou-2018-1	Associations between angiotensinogen M235T polymorphisms and the risk of diabetic nephropathy: A meta-analysis	1/2	Yes	No	No	Yes	No	No	Partial Yes	Yes	Yes	No	Yes	Yes	Yes	Yes	Yes	Yes	Low
Zhou-2018-2	Relationship between transforming growth factor-beta1 and type 2 diabetic nephropathy risk in Chinese population	2	Yes	No	No	Yes	No	No	Partial Yes	Yes	No	No	Yes	No	No	No	Yes	Yes	Critically low
Zintzaras-2005	Association between the GLUT1 gene polymorphism and the risk of diabetic nephropathy: A meta-analysis	1/2	Yes	No	No	Yes	No	No	Partial Yes	Yes	No	No	Yes	No	No	Yes	Yes	Yes	Critically low

Item 1: Did the research questions and inclusion criteria for the review include the components of PICO?
Item 2: Did the report of the review contain an explicit statement that the review methods were established prior to the conduct of the review and did the report justify any significant deviations from the protocol?
Item 3: Did the review authors explain their selection of the study designs for inclusion in the review?
Item 4: Did the review authors use a comprehensive literature search strategy?
Item 5: Did the review authors perform study selection in duplicate?
Item 6: Did the review authors perform data extraction in duplicate?
Item 7: Did the review authors provide a list of excluded studies and justify the exclusions?
Item 8: Did the review authors describe the included studies in adequate detail?
Item 9: Did the review authors use a satisfactory technique for assessing the risk of bias (RoB) in individual studies that were included in the review?
Item 10: Did the review authors report on the sources of funding for the studies included in the review?
Item 11: If meta-analysis was performed did the review authors use appropriate methods for statistical combination of results?
Item 12: If meta-analysis was performed, did the review authors assess the potential impact of RoB in individual studies on the results of the meta-analysis or other evidence synthesis?
Item 13: Did the review authors account for RoB in individual studies when interpreting/ discussing the results of the review?
Item 14: Did the review authors provide a satisfactory explanation for, and discussion of, any heterogeneity observed in the results of the review?
Item 15: If they performed quantitative synthesis did the review authors carry out an adequate investigation of publication bias (small study bias) and discuss its likely impact on the results of the review?
Item 16: Did the review authors report any potential sources of conflict of interest, including any funding they received for conducting the review?
Among the above 16 items, items 2, 4, 7, 9, 11, 13, and 15 are critical domains. The detials of this checklist can be found at https://amstar.ca/Amstar_Checklist.php.

Supplementary Table 3. Summary of "credible" and "not credible" associations in type 1 diabetes.

Factor	A1/A2	Outcome	Population	No. study	Sample size (cases/controls)	Genetic model	OR (95% CIs)	P.effct	I2	P.Egger	P.Sig	Power	Venice criteria	BFDP1	BFDP2	BFDP3	BFDP4	Credibility
ACE Ins/Del	Del/Ins	DR	European	6	519/452	allelic model	1.09 (0.86 , 1.38)	0.482	33.79%	0.495	0.565	0.159	CBB	0.965	0.999	1.000	1.000	Not credible
		PDR	European	3	300/185	allelic model	1.11 (0.66 , 1.89)	0.687	69.61%	0.236	0.662	0.266	CCB	0.966	0.999	1.000	1.000	Not credible
ALR (AC)n	NA	DR	European/African	3	306/178	allele (Z), present vs null	1.03 (0.73 , 1.43)	0.885	22.48%	0.504	1.000	0.028	CAB	0.965	0.999	1.000	1.000	Credible
				3	306/178	allele (Z+2), present vs null	0.43 (0.26 , 0.72)	0.001	38.95%	0.181	0.958	0.997	ABB	0.836	0.996	1.000	1.000	
				3	306/178	allele (Z-2), present vs null	1.6 (0.83 , 3.07)	0.161	74.38%	0.216	0.846	0.923	ACB	0.947	0.999	1.000	1.000	
				3	306/178	allele (Z+4), present vs null	0.77 (0.33 , 1.79)	0.537	22.02%	0.691	1.000	0.094	CAB	0.956	0.999	1.000	1.000	
				3	306/178	allele (Z-4), present vs null	1.78 (0.84 , 3.78)	0.136	3.45%	0.506	1.000	0.292	CAB	0.943	0.999	1.000	1.000	
				3	306/178	allele (Z+6), present vs null	0.57 (0.13 , 2.55)	0.464	16.15%	0.335	1.000	0.081	CAB	0.954	0.999	1.000	1.000	
				3	306/178	allele (Z-6), present vs null	1.5 (0.31 , 7.39)	0.616	5.24%	0.566	1.000	0.051	CAB	0.966	0.999	1.000	1.000	
AKR1B1 rs759853	T/C	DR	European/Asian	3	298/227	allelic model	0.58 (0.42 , 0.8)	0.001	12.60%	0.333	0.524	0.940	AAB	0.794	0.995	1.000	1.000	Credible
				3	298/227	dominant model	0.52 (0.34 , 0.79)	0.002	11.49%	0.305	0.406	0.915	AAB	0.875	0.997	1.000	1.000	
				3	298/227	heterozygous model	0.56 (0.37 , 0.83)	0.004	2.56%	0.521	0.806	0.828	AAB	0.907	0.998	1.000	1.000	
ACE Ins/Del	Del/Ins	DKD	European/Asian	12	1262/1014	allelic model	1.14 (0.95 , 1.36)	0.151	43.11%	0.108	0.345	0.418	CBB	0.945	0.999	1.000	1.000	Not credible
				12	1262/1014	dominant model	1.29 (0.95 , 1.76)	0.105	38.66%	0.249	0.324	0.628	BBB	0.936	0.999	1.000	1.000	
				12	1262/1014	recessive model	1.07 (0.86 , 1.34)	0.549	25.72%	0.074	0.715	0.098	CBC	0.965	0.999	1.000	1.000	
				12	1262/1014	codominant model	1.1 (0.93 , 1.31)	0.270	0.00%	0.400	1.000	0.180	CAB	0.959	0.999	1.000	1.000	
AGT rs699 M235T	C/T	DKD	European/Asian	10	1494/1500	allelic model	1.11 (0.95 , 1.28)	0.187	42.00%	0.167	0.847	0.333	CBB	0.946	0.999	1.000	1.000	Not credible
				10	1494/1500	dominant model	1.1 (0.89 , 1.35)	0.372	30.13%	0.596	0.712	0.159	CBB	0.962	0.999	1.000	1.000	
				10	1494/1500	recessive model	1.21 (0.91 , 1.61)	0.186	40.73%	0.028	0.841	0.361	CBC	0.952	0.999	1.000	1.000	
				10	1494/1500	heterozygous model	1.04 (0.85 , 1.26)	0.720	17.30%	0.683	1.000	0.053	CAB	0.966	0.999	1.000	1.000	
				10	1494/1500	homozygous model	1.28 (0.91 , 1.81)	0.156	42.82%	0.190	0.562	0.438	CBB	0.948	0.999	1.000	1.000	
AGTR1 rs5186	C/A	DKD	European	3	262/464	allelic model	0.97 (0.67 , 1.41)	0.879	56.61%	0.047	1.000	0.061	CCC	0.962	0.999	1.000	1.000	Not credible
				3	262/464	dominant model	0.89 (0.52 , 1.53)	0.672	64.95%	0.096	1.000	0.172	CCC	0.959	0.999	1.000	1.000	
				3	262/464	recessive model	1.25 (0.72 , 2.16)	0.421	0.19%	0.755	1.000	0.103	CAB	0.964	0.999	1.000	1.000	
				3	262/464	heterozygous model	0.84 (0.48 , 1.48)	0.549	64.36%	0.073	0.617	0.265	CCC	0.956	0.999	1.000	1.000	
				3	262/464	homozygous model	1.15 (0.64 , 2.08)	0.644	7.83%	0.388	1.000	0.052	CAB	0.966	0.999	1.000	1.000	
ALR (AC)n	NA	DKD	European	10	1219/1164	allele (Z-2), present vs null	1.4 (1.07 , 1.83)	0.014	72.53%	0.007	0.598	0.906	ACC	0.788	0.995	0.999	1.000	Credible
				10	1219/1164	allele (Z+2), present vs null	0.81 (0.62 , 1.06)	0.121	47.52%	0.183	0.651	0.891	ABB	0.949	0.999	1.000	1.000	
AKR1B1 rs759853	T/C	DKD	European	4	740/537	allelic model	1.6 (1.06 , 2.42)	0.026	82.45%	0.009	0.527	0.986	ACC	0.854	0.997	1.000	1.000	Credible
				4	740/537	dominant model	1.88 (1.16 , 3.04)	0.011	74.31%	0.028	0.495	0.992	ACC	0.746	0.994	0.999	1.000	
				4	740/537	recessive model	1.58 (0.87 , 2.85)	0.130	68.79%	0.010	0.875	0.494	CCC	0.942	0.999	1.000	1.000	
				4	740/537	homozygous model	2.17 (1.04 , 4.55)	0.040	76.33%	0.003	0.376	0.911	ACC	0.888	0.998	1.000	1.000	
				4	740/537	codominant model	1.4 (1.08 , 1.81)	0.010	17.87%	0.217	0.788	0.784	BAB	0.749	0.994	0.999	1.000	
eNOS rs1799983	T/G	DKD	European	3	1556/1065	allelic model	0.98 (0.83 , 1.15)	0.788	35.35%	0.834	1.000	0.056	CBB	0.961	0.999	1.000	1.000	Not credible
eNOS rs869109213	4a/4b	DKD	European	4	1609/1192	allelic model	1.08 (0.86 , 1.36)	0.512	45.34%	0.357	1.000	0.116	CBB	0.965	0.999	1.000	1.000	Not credible
				4	1609/1192	dominant model	1.07 (0.81 , 1.43)	0.619	51.97%	0.356	1.000	0.075	CCB	0.966	0.999	1.000	1.000	
				3	1567/1070	recessive model	1.24 (0.77 , 1.98)	0.374	3.33%	0.668	1.000	0.121	CAB	0.962	0.999	1.000	1.000	

				4	1609/1192	codominant model	1.05 (0.77 , 1.42)	0.769	56.21%	0.355	1.000	0.037	CCB	0.966	0.999	1.000	1.000	
GLUT1 XbαI	1.1/0.9	DKD	European	3	494/443	allelic model	1.41 (0.94 , 2.13)	0.096	74.33%	0.077	0.889	0.772	BCC	0.934	0.999	1.000	1.000	Not credible
				3	494/443	dominant model	1.48 (0.94 , 2.34)	0.089	58.46%	0.109	0.811	0.646	BCB	0.931	0.999	1.000	1.000	
				3	494/443	recessive model	1.61 (0.64 , 4.03)	0.310	78.75%	0.352	0.413	0.475	CCB	0.960	0.999	1.000	1.000	
				3	494/443	homozygous model	1.98 (0.69 , 5.66)	0.202	80.38%	0.173	0.495	0.625	BCB	0.953	0.999	1.000	1.000	
SERPINE1 rs1799889	4G/5G	DKD	European	3	594/688	allelic model	1.06 (0.9 , 1.25)	0.460	2.51%	0.862	1.000	0.105	CAB	0.965	0.999	1.000	1.000	Not credible
				3	594/688	dominant model	1.16 (0.86 , 1.56)	0.323	13.60%	0.561	1.000	0.170	CAB	0.961	0.999	1.000	1.000	
				3	594/688	recessive model	1.04 (0.71 , 1.51)	0.842	55.71%	0.516	1.000	0.033	CCB	0.966	0.999	1.000	1.000	
				3	594/688	heterozygous model	1.16 (0.77 , 1.75)	0.469	48.44%	0.541	1.000	0.170	CBB	0.965	0.999	1.000	1.000	
				3	594/688	homozygous model	1.16 (0.84 , 1.6)	0.369	0.72%	0.860	1.000	0.127	CAB	0.962	0.999	1.000	1.000	
CCR5 rs1799987	A/G	Microvascular complications	European	3	927/815	dominant model	0.78 (0.47 , 1.3)	0.347	70.76%	0.071	0.750	0.208	CCC	0.951	0.999	1.000	1.000	Not credible

A1/A2, alternative allele and reference allele for the genetic variants; allelic model, A1 vs. A2, dominant model, A1A1+A1A2 vs. A2A2, recessive model, A1A1 vs. A1A2+A2A2, heterozygous model, A1A2 vs A2A2, homozygous model, A1A1 vs A2A2, codominant model, A1A2 vs. A1A1+A2A2; P.effect, the P-value for meta-analyses effects; P.Egger, the P-value for small study effects; P.Sig, the P-value for excess significance; BFDP1, BFDP2, BFDP3, and BFDP4 were respectively calculated at the prior level of 0.05, 10-3, 10-4, and 10-6. DR, diabetic retinopathy; PDR, proliferative diabetic retinopathy; DKD, diabetic kidney disease.

Supplementary Table 4. Summary of "credible" and "not credible" associations in type 2 diabetes.

Factor	A1/A2	Outcome	Population	No. study	Sample size (cases/controls)	Genetic model	OR (95% CIs)	P.effct	I2	P.Egger	P.Sig	Power	Venice criteria	BFDP1	BFDP2	BFDP3	BFDP4	Credibility
ACE Ins/Del	Del/Ins	DR	European/Asian	7	1146/1104	allelic model	1.02 (0.9 , 1.15)	0.779	0.00%	0.238	1.000	0.043	CAB	0.966	0.999	1.000	1.000	Not credible
			European	4	478/468	allelic model	1.01 (0.83 , 1.23)	0.933	12.96%	0.245	1.000	0.024	CAB	0.965	0.999	1.000	1.000	Not credible
		PDR	European/Asian	4	372/716	allelic model	1.13 (0.86 , 1.5)	0.383	43.14%	0.922	0.552	0.217	CBB	0.963	0.999	1.000	1.000	Not credible
		NPDR	European/Asian	3	360/626	allelic model	1.07 (0.8 , 1.41)	0.657	34.04%	0.092	1.000	0.026	CBC	0.966	0.999	1.000	1.000	Not credible
AGER rs1800624	A/T	DR	European/Asian	8	1786/2213	allelic model	1.1 (0.96 , 1.26)	0.186	17.71%	0.097	0.619	0.261	CAC	0.949	0.999	1.000	1.000	Credible
				8	1580/2158	dominant model	1.19 (1.02 , 1.38)	0.027	0.00%	0.473	0.656	0.590	BAB	0.836	0.996	1.000	1.000	
				6	1129/1690	recessive model	0.92 (0.55 , 1.55)	0.764	42.59%	0.010	1.000	0.194	CBC	0.961	0.999	1.000	1.000	
				7	1474/2049	heterozygous model	1.24 (1.05 , 1.46)	0.010	0.00%	0.710	1.000	0.723	BAB	0.743	0.993	0.999	1.000	
				6	1129/1690	homozygous model	1.01 (0.6 , 1.71)	0.968	41.83%	0.012	1.000	0.072	CBC	0.964	0.999	1.000	1.000	
			European	4	1004/991	allelic model	1.01 (0.87 , 1.16)	0.928	8.72%	0.489	1.000	0.030	CAB	0.965	0.999	1.000	1.000	Credible
				4	798/1361	dominant model	1.12 (0.93 , 1.34)	0.236	3.59%	0.573	1.000	0.219	CAB	0.954	0.999	1.000	1.000	
				3	692/1252	recessive model	0.65 (0.45 , 0.94)	0.023	0.58%	0.721	1.000	0.599	BAB	0.947	0.999	1.000	1.000	
				3	692/1252	heterozygous model	1.22 (1 , 1.5)	0.056	4.15%	0.649	1.000	0.487	CAB	0.911	0.998	1.000	1.000	
				3	692/1252	homozygous model	0.71 (0.48 , 1.04)	0.077	1.04%	0.651	1.000	0.385	CAB	0.949	0.999	1.000	1.000	
			Asian	4	782/797	allelic model	1.42 (1.06 , 1.9)	0.019	21.31%	0.782	0.761	0.740	BAB	0.820	0.996	1.000	1.000	Credible
				4	782/797	dominant model	1.4 (1 , 1.95)	0.053	25.34%	0.627	0.678	0.573	BBB	0.897	0.998	1.000	1.000	
				3	437/438	recessive model	2.89 (1.12 , 7.49)	0.029	1.02%	0.617	1.000	0.566	BAB	0.864	0.997	1.000	1.000	
				4	782/797	heterozygous model	1.31 (0.93 , 1.84)	0.122	23.22%	0.473	1.000	0.371	CAB	0.939	0.999	1.000	1.000	
				3	437/438	homozygous model	3.14 (1.21 , 8.17)	0.019	1.13%	0.652	1.000	0.632	BAB	0.825	0.996	1.000	1.000	
AGER rs184003	T/G	DR	European/Asian	3	613/1219	dominant model	0.97 (0.74 , 1.28)	0.844	19.73%	0.352	1.000	0.026	CAB	0.962	0.999	1.000	1.000	Not credible
AGER rs1800625	C/T	DR	European/Asian	6	1300/1563	dominant model	1 (0.78 , 1.28)	0.992	49.80%	0.357	0.672	0.042	CBB	0.964	0.999	1.000	1.000	Not credible
AGER rs2070600	A/G	DR	Asian	6	1547/1893	dominant model	0.86 (0.5 , 1.48)	0.584	87.71%	0.113	0.067	0.205	CCB	0.957	0.999	1.000	1.000	Not credible
ALR (AC)n	NA	DR	European/Asian	12	930/1173	allele (Z), present vs null	0.9 (0.78 , 1.04)	0.167	0.00%	0.646	0.574	0.268	CAB	0.948	0.999	1.000	1.000	Credible
				12	930/1173	allele (Z+2), present vs null	0.72 (0.52 , 0.99)	0.043	68.03%	0.000	0.796	0.232	CCC	0.950	0.999	1.000	1.000	
				12	930/1173	allele (Z-2), present vs null	1.59 (1.19 , 2.12)	0.002	54.73%	0.052	0.353	0.991	ACC	0.406	0.973	0.997	1.000	
				10	826/1115	allele (Z+4), present vs null	0.75 (0.58 , 0.97)	0.031	0.00%	0.409	1.000	0.528	BAB	0.949	0.999	1.000	1.000	
				11	883/1145	allele (Z-4), present vs null	1.24 (0.87 , 1.77)	0.227	39.54%	0.990	0.851	0.413	CBB	0.956	0.999	1.000	1.000	
				9	737/1077	allele (Z+6), present vs null	0.87 (0.51 , 1.48)	0.603	31.66%	0.350	1.000	0.161	CBB	0.958	0.999	1.000	1.000	
				9	674/572	allele (Z-6), present vs null	0.7 (0.4 , 1.24)	0.223	0.00%	0.412	1.000	0.181	CAB	0.949	0.999	1.000	1.000	
AKR1B1 rs759853	T/C	DR	European/Asian/ Middle Eastern/African	19	3946/4673	allelic model	0.96 (0.81 , 1.13)	0.614	73.57%	0.898	0.394	0.417	CCB	0.958	0.999	1.000	1.000	Not credible
				19	3946/4673	dominant model	0.93 (0.75 , 1.16)	0.526	74.39%	0.579	0.038	0.576	BCB	0.955	0.999	1.000	1.000	
				19	3946/4673	heterozygous model	0.93 (0.74 , 1.16)	0.494	72.19%	0.576	0.077	0.595	BCB	0.955	0.999	1.000	1.000	
			Middle Eastern	3	214/157	allelic model	0.48 (0.3 , 0.78)	0.003	41.74%	0.313	0.950	0.972	ABB	0.896	0.998	1.000	1.000	Credible
				3	214/157	dominant model	0.41 (0.24 , 0.69)	0.001	21.82%	0.202	0.138	0.971	AAB	0.777	0.995	0.999	1.000	
				3	214/157	heterozygous model	0.44 (0.26 , 0.73)	0.002	9.78%	0.329	0.413	0.905	AAB	0.846	0.997	1.000	1.000	
eNOS rs1799983	T/G	DR	European/Asian/ African	11	2576/2751	allelic model	1.06 (0.96 , 1.16)	0.237	0.00%	0.933	0.522	0.211	CAB	0.953	0.999	1.000	1.000	Not credible
				11	2576/2751	dominant model	1.08 (0.93 , 1.25)	0.306	22.40%	0.583	0.318	0.259	CAB	0.960	0.999	1.000	1.000	
				11	2444/2774	recessive model	1.02 (0.83 , 1.26)	0.820	0.00%	0.419	0.435	0.036	CAB	0.965	0.999	1.000	1.000	
				11	2576/2751	codominant model	1.07 (0.95 , 1.21)	0.243	0.00%	0.801	0.520	0.202	CAB	0.959	0.999	1.000	1.000	

eNOS rs1799983	T/G	DR	European	7	2001/2066	allelic model	1.07 (0.94 , 1.21)	0.299	28.55%	0.420	0.634	0.244	CBB	0.959	0.999	1.000	1.000	Not credible
				7	2001/2066	dominant model	1.11 (0.9 , 1.36)	0.344	49.74%	0.249	0.409	0.297	CBB	0.960	0.999	1.000	1.000	
				8	2044/2233	recessive model	1.03 (0.84 , 1.26)	0.791	0.00%	0.547	0.342	0.030	CAB	0.966	0.999	1.000	1.000	
				7	2001/2066	codominant model	1.07 (0.9 , 1.28)	0.430	34.42%	0.512	0.645	0.211	CBB	0.964	0.999	1.000	1.000	
			Asian	3	507/380	allelic model	0.96 (0.7 , 1.33)	0.816	10.06%	0.363	1.000	0.035	CAB	0.961	0.999	1.000	1.000	Not credible
				3	507/380	dominant model	0.96 (0.69 , 1.34)	0.830	1.84%	0.575	1.000	0.034	CAB	0.961	0.999	1.000	1.000	
				3	507/380	codominant model	0.97 (0.69 , 1.35)	0.851	0.03%	0.870	1.000	0.032	CAB	0.962	0.999	1.000	1.000	
eNOS rs2070744	C/T	DR	European/Asian	5	1831/1512	allelic model	1.05 (0.9 , 1.22)	0.534	35.65%	0.208	0.534	0.195	CBB	0.965	0.999	1.000	1.000	Not credible
				5	1831/1512	dominant model	1.09 (0.94 , 1.25)	0.241	0.00%	0.383	0.350	0.205	CAB	0.954	0.999	1.000	1.000	
				5	1831/1512	recessive model	1.04 (0.75 , 1.45)	0.793	35.17%	0.252	1.000	0.062	CBB	0.966	0.999	1.000	1.000	
				5	1831/1512	codominant model	1.07 (0.92 , 1.23)	0.368	0.00%	0.943	1.000	0.137	CAB	0.961	0.999	1.000	1.000	
eNOS rs869109213	4a/4b	DR	European/Asian/ African	13	3076/3103	allelic model	1 (0.84 , 1.2)	0.985	68.05%	0.731	0.400	0.105	CCB	0.964	0.999	1.000	1.000	Not credible
				13	3076/3103	dominant model	0.97 (0.79 , 1.18)	0.734	64.60%	0.822	0.614	0.208	CCB	0.961	0.999	1.000	1.000	
				13	3076/3103	recessive model	1.14 (0.8 , 1.62)	0.466	38.13%	0.735	0.858	0.074	CBB	0.964	0.999	1.000	1.000	
				13	3076/3103	codominant model	0.94 (0.8 , 1.11)	0.474	42.68%	0.894	0.288	0.312	CBB	0.954	0.999	1.000	1.000	
			European	7	2413/2195	allelic model	1.08 (0.85 , 1.37)	0.530	78.18%	0.355	0.472	0.041	CCB	0.965	0.999	1.000	1.000	Not credible
				7	2413/2195	dominant model	1.06 (0.81 , 1.37)	0.683	73.41%	0.183	0.690	0.075	CCB	0.966	0.999	1.000	1.000	
				7	2413/2195	recessive model	1.19 (0.77 , 1.83)	0.430	53.22%	0.978	0.813	0.068	CCB	0.964	0.999	1.000	1.000	
				7	2413/2195	codominant model	1 (0.82 , 1.22)	0.993	52.33%	0.058	0.360	0.125	CCC	0.964	0.999	1.000	1.000	
			Asian	5	595/607	allelic model	1.01 (0.79 , 1.29)	0.926	8.81%	0.118	1.000	0.029	CAB	0.965	0.999	1.000	1.000	Not credible
				5	595/607	dominant model	0.99 (0.77 , 1.29)	0.963	0.00%	0.336	1.000	0.024	CAB	0.963	0.999	1.000	1.000	
				5	595/607	recessive model	1.05 (0.32 , 3.45)	0.933	36.95%	0.386	1.000	0.054	CBB	0.965	0.999	1.000	1.000	
				5	595/607	codominant model	0.96 (0.74 , 1.26)	0.788	0.00%	0.844	1.000	0.039	CAB	0.961	0.999	1.000	1.000	
EPO rs1617640	T/G	DR	Asian	4	1128/1105	allelic model	0.92 (0.65 , 1.31)	0.660	81.74%	0.095	0.882	0.034	CCC	0.958	0.999	1.000	1.000	Not credible
				4	1128/1105	dominant model	0.77 (0.18 , 3.22)	0.718	92.65%	0.072	0.974	0.025	CCC	0.960	0.999	1.000	1.000	
				4	1128/1105	recessive model	0.96 (0.7 , 1.32)	0.806	56.23%	0.544	0.601	0.034	CCB	0.961	0.999	1.000	1.000	
				4	1128/1105	heterozygous model	0.79 (0.21 , 3)	0.732	90.61%	0.096	0.962	0.028	CCC	0.960	0.999	1.000	1.000	
				4	1128/1105	homozygous model	0.77 (0.17 , 3.44)	0.732	91.21%	0.032	0.958	0.020	CCC	0.960	0.999	1.000	1.000	
GSTM1	NA	DR	European	4	556/608	Gene (GSTM1), null vs present	0.42 (0.25 , 0.69)	0.001	60.10%	0.410	0.539	1.000	ACB	0.743	0.993	0.999	1.000	Credible
GSTT1	NA	DR	European	4	556/608	Gene (GSTT1), null vs present	1.56 (0.97 , 2.52)	0.068	57.31%	0.926	0.878	0.975	ACB	0.918	0.998	1.000	1.000	Not credible
Hp rs137853233	T/C	DR	Asian/African	4	520/461	allelic model	0.6 (0.2 , 1.77)	0.358	95.84%	0.000	0.992	0.140	CCC	0.951	0.999	1.000	1.000	Not credible
				4	520/461	dominant model	0.72 (0.31 , 1.68)	0.444	39.40%	0.595	1.000	0.111	CBB	0.954	0.999	1.000	1.000	
				4	520/461	recessive model	0.55 (0.15 , 2.08)	0.381	94.93%	0.004	0.987	0.109	CCC	0.952	0.999	1.000	1.000	
				4	520/461	heterozygous model	1.44 (0.5 , 4.14)	0.497	59.26%	0.425	0.697	0.017	CCB	0.965	0.999	1.000	1.000	
				4	520/461	homozygous model	0.65 (0.25 , 1.74)	0.395	48.87%	0.505	0.621	0.140	CBB	0.952	0.999	1.000	1.000	
				4	520/461	additive model	0.6 (0.13 , 2.75)	0.508	96.04%	0.019	0.989	0.053	CCC	0.955	0.999	1.000	1.000	
ICAM1 rs5498	G/A	DR	European/Asian	5	952/749	dominant model	0.67 (0.42 , 1.07)	0.092	73.77%	0.079	0.360	0.661	BCC	0.949	0.999	1.000	1.000	Credible
				5	952/749	recessive model	0.88 (0.55 , 1.42)	0.608	69.19%	0.207	0.441	0.037	CCB	0.957	0.999	1.000	1.000	
				5	952/749	additive model	1.22 (1 , 1.48)	0.045	0.00%	0.144	1.000	0.503	BAB	0.893	0.998	1.000	1.000	
IL-6 rs1800795	C/G	DR	European/Asian	3	504/617	allelic model	0.89 (0.54 , 1.45)	0.637	83.27%	0.013	0.828	0.024	CCC	0.958	0.999	1.000	1.000	Credible
				3	504/617	dominant model	1.21 (0.89 , 1.65)	0.230	26.39%	0.431	1.000	0.318	CBB	0.955	0.999	1.000	1.000	
				3	504/617	recessive model	0.45 (0.06 , 3.56)	0.451	91.51%	0.093	0.940	0.674	BCC	0.954	0.999	1.000	1.000	
				3	504/617	heterozygous model	1.36 (1.01 , 1.84)	0.046	18.48%	0.651	0.640	0.583	BAB	0.897	0.998	1.000	1.000	
				3	504/617	homozygous model	0.56 (0.08 , 4.13)	0.570	90.19%	0.070	0.918	0.104	CCC	0.957	0.999	1.000	1.000	
				3	504/617	codominant model	1.64 (0.96 , 2.81)	0.069	74.68%	0.099	0.557	0.855	ACC	0.920	0.998	1.000	1.000	
MTHFR rs1801131	C/A	DR	Middle Eastern	3	92/312	allelic model	1.99 (0.79 , 5)	0.141	80.93%	0.783	0.906	0.937	ACB	0.945	0.999	1.000	1.000	Credible
				4	159/447	dominant model	2.95 (1.33 , 6.59)	0.008	63.26%	0.333	0.769	1.000	ACB	0.718	0.993	0.999	1.000	

				4	159/447	recessive model	1.5 (0.53 , 4.29)	0.447	59.11%	0.438	0.695	0.937	ACB	0.964	0.999	1.000	1.000	
MTHFR rs1801133	T/C	DR	Asian	6	506/646	allelic model	2.14 (1.55 , 2.97)	4.56E-06	69.47%	0.703	0.578	1.000	ACB	0.005	0.208	0.724	0.996	Credible
				6	506/646	dominant model	2.86 (1.86 , 4.4)	1.74E-06	53.45%	0.389	0.421	1.000	ACB	0.002	0.087	0.487	0.990	
				6	506/646	recessive model	2.48 (1.52 , 4.05)	2.95E-04	60.26%	0.239	0.495	1.000	ACB	0.140	0.895	0.988	1.000	
				6	506/646	homozygous model	4.19 (2.09 , 8.41)	5.63E-05	69.61%	0.502	0.386	1.000	ACB	0.038	0.677	0.954	1.000	
PPARγ rs1801282	G/C	DR	European/Asian	8	2898/2352	dominant model	0.81 (0.62 , 1.06)	0.123	52.73%	0.203	0.890	0.681	BCB	0.949	0.999	1.000	1.000	Not credible
			European	6	740/1549	dominant model	0.91 (0.67 , 1.23)	0.530	46.93%	0.155	1.000	0.187	CBB	0.956	0.999	1.000	1.000	Not credible
SERPINE1 rs1799889	4G/5G	DR	European/Asian/ African	10	1388/1511	allelic model	1.09 (0.97 , 1.22)	0.158	10.57%	0.395	0.236	0.374	CAB	0.943	0.999	1.000	1.000	Not credible
				10	1388/1511	dominant model	1.05 (0.83 , 1.34)	0.669	36.09%	0.840	0.741	0.081	CBB	0.966	0.999	1.000	1.000	
				10	1388/1511	recessive model	1.17 (0.96 , 1.44)	0.125	23.12%	0.213	0.740	0.541	BAB	0.944	0.999	1.000	1.000	
				10	1388/1511	heterozygous model	1 (0.76 , 1.32)	0.975	44.17%	0.559	0.798	0.045	CBB	0.964	0.999	1.000	1.000	
				10	1388/1511	homozygous model	1.22 (0.94 , 1.57)	0.137	20.09%	0.248	0.715	0.513	BAB	0.940	0.999	1.000	1.000	
			European	5	880/1071	allelic model	1.12 (0.98 , 1.27)	0.092	0.00%	0.227	0.468	0.379	CAB	0.923	0.998	1.000	1.000	Credible
				5	880/1071	dominant model	1 (0.82 , 1.22)	0.977	0.00%	0.658	1.000	0.024	CAB	0.964	0.999	1.000	1.000	
				5	880/1071	recessive model	1.35 (1.03 , 1.75)	0.028	25.68%	0.228	0.773	0.807	ABB	0.845	0.997	1.000	1.000	
				5	880/1071	heterozygous model	0.88 (0.71 , 1.09)	0.241	0.00%	0.924	1.000	0.200	CAB	0.949	0.999	1.000	1.000	
				5	880/1071	homozygous model	1.3 (0.98 , 1.73)	0.066	9.20%	0.128	0.577	0.529	BAB	0.920	0.998	1.000	1.000	
			Asian	4	438/338	allelic model	0.95 (0.74 , 1.23)	0.702	31.74%	0.709	1.000	0.065	CBB	0.959	0.999	1.000	1.000	Not credible
				4	438/338	dominant model	0.93 (0.58 , 1.51)	0.783	32.80%	0.670	1.000	0.043	CBB	0.961	0.999	1.000	1.000	
				4	438/338	recessive model	0.94 (0.66 , 1.33)	0.710	21.92%	0.406	1.000	0.059	CAB	0.960	0.999	1.000	1.000	
				4	438/338	heterozygous model	0.95 (0.57 , 1.57)	0.840	31.21%	0.480	1.000	0.034	CBB	0.962	0.999	1.000	1.000	
				4	438/338	homozygous model	0.93 (0.55 , 1.59)	0.799	31.82%	0.875	1.000	0.038	CBB	0.961	0.999	1.000	1.000	
TCF7L2 rs7903146	T/C	DR	European/Asian	6	1976/3569	allelic model	1.16 (0.99 , 1.36)	0.074	63.38%	0.715	0.340	0.920	ACB	0.917	0.998	1.000	1.000	Credible
				8	2400/4022	dominant model	1.14 (0.91 , 1.44)	0.245	64.35%	0.077	0.929	0.618	BCC	0.958	0.999	1.000	1.000	
				6	1976/3569	heterozygous model	1.08 (0.86 , 1.35)	0.497	61.12%	0.337	0.810	0.205	CCB	0.965	0.999	1.000	1.000	
				6	1976/3569	homozygous model	1.47 (1.19 , 1.81)	3.73E-04	10.35%	0.626	0.334	0.966	AAB	0.140	0.895	0.988	1.000	
			European	5	1497/2919	allelic model	1.21 (1.05 , 1.41)	0.011	54.81%	0.283	0.369	0.970	ACB	0.795	0.995	1.000	1.000	Credible
				6	1509/3034	dominant model	1.27 (0.98 , 1.63)	0.066	63.56%	0.036	0.939	0.870	ACC	0.912	0.998	1.000	1.000	
				5	1497/2919	heterozygous model	1.15 (0.91 , 1.45)	0.246	57.52%	0.303	0.789	0.422	CCB	0.956	0.999	1.000	1.000	
				5	1497/2919	homozygous model	1.47 (1.16 , 1.87)	0.001	28.20%	0.349	0.327	0.965	ABB	0.421	0.975	0.997	1.000	
TGF-β1 rs1800469	T/C	DR	European/Asian	3	521/580	allelic model	0.81 (0.66 , 0.99)	0.039	8.10%	0.407	1.000	0.561	BAB	0.950	0.999	1.000	1.000	Credible
				3	521/580	dominant model	0.75 (0.56 , 1.01)	0.057	23.90%	0.256	0.534	0.550	BAB	0.950	0.999	1.000	1.000	
				3	521/580	recessive model	0.8 (0.53 , 1.21)	0.288	0.19%	0.930	1.000	0.159	CAB	0.950	0.999	1.000	1.000	
				3	521/580	homozygous model	0.72 (0.46 , 1.12)	0.141	0.00%	0.908	1.000	0.276	CAB	0.948	0.999	1.000	1.000	
TLR4 rs4986790	G/A	DR	European/Asian	5	944/1840	allelic model	1.77 (1.05 , 2.98)	0.031	73.80%	0.303	0.466	0.990	ACB	0.871	0.997	1.000	1.000	Credible
				5	944/1840	dominant model	1.81 (1.04 , 3.14)	0.035	74.12%	0.277	0.459	0.989	ACB	0.878	0.997	1.000	1.000	
TNF-α rs1800629	A/G	DR	Asian	4	599/788	allelic model	1.05 (0.79 , 1.39)	0.742	5.16%	0.575	1.000	0.049	CAB	0.966	0.999	1.000	1.000	Not credible
				4	599/788	dominant model	1.05 (0.77 , 1.44)	0.745	5.38%	0.654	1.000	0.046	CAB	0.966	0.999	1.000	1.000	
				3	524/612	recessive model	1.02 (0.3 , 3.46)	0.981	20.88%	0.662	1.000	0.025	CAB	0.964	0.999	1.000	1.000	
				4	599/788	heterozygous model	1.05 (0.75 , 1.47)	0.767	7.80%	0.748	1.000	0.043	CAB	0.966	0.999	1.000	1.000	
				3	524/612	homozygous model	1.04 (0.31 , 3.5)	0.955	20.20%	0.656	1.000	0.025	CAB	0.965	0.999	1.000	1.000	
			European/Asian	7	1364/1729	allelic model	1.09 (0.91 , 1.31)	0.345	17.01%	0.716	0.523	0.174	CAB	0.962	0.999	1.000	1.000	Not credible
				7	1364/1729	dominant model	1.1 (0.92 , 1.31)	0.291	0.00%	0.919	0.393	0.173	CAB	0.959	0.999	1.000	1.000	
				6	1289/1553	recessive model	1.09 (0.6 , 1.98)	0.782	15.51%	0.497	1.000	0.044	CAB	0.966	0.999	1.000	1.000	
				7	1364/1729	heterozygous model	1.09 (0.91 , 1.31)	0.329	0.00%	0.853	1.000	0.152	CAB	0.962	0.999	1.000	1.000	
				6	1289/1553	homozygous model	1.11 (0.59 , 2.1)	0.750	20.97%	0.511	1.000	0.044	CAB	0.966	0.999	1.000	1.000	
				5	798/618	allelic model	1.36 (0.78 , 2.37)	0.274	86.15%	0.008	0.973	0.491	CCC	0.959	0.999	1.000	1.000	
				5	798/618	dominant model	1.25 (0.77 , 2.04)	0.369	72.98%	0.001	0.881	0.135	CCC	0.962	0.999	1.000	1.000	

VEGF rs1570360	A/G	DR	European/Asian	5	798/618	recessive model	1.83 (0.55 , 6.09)	0.325	73.55%	0.550	0.892	0.858	ACB	0.961	0.999	1.000	1.000	Not credible
				5	798/618	heterozygous model	1.08 (0.76 , 1.53)	0.669	43.35%	0.045	1.000	0.047	CBC	0.966	0.999	1.000	1.000	
				5	798/618	homozygous model	2.08 (0.5 , 8.57)	0.313	77.48%	0.737	0.921	0.800	ACB	0.960	0.999	1.000	1.000	
			Asian	4	753/557	allelic model	1.02 (0.75 , 1.38)	0.920	50.04%	0.515	1.000	0.025	CCB	0.965	0.999	1.000	1.000	Not credible
				4	753/557	dominant model	1.01 (0.72 , 1.41)	0.969	46.58%	0.459	1.000	0.022	CBB	0.965	0.999	1.000	1.000	
				4	753/557	recessive model	1.1 (0.4 , 2.98)	0.854	39.10%	0.864	1.000	0.027	CBB	0.965	0.999	1.000	1.000	
				4	753/557	heterozygous model	0.99 (0.72 , 1.38)	0.971	38.30%	0.564	1.000	0.026	CBB	0.964	0.999	1.000	1.000	
				4	753/557	homozygous model	1.11 (0.39 , 3.14)	0.838	42.44%	0.886	1.000	0.030	CBB	0.965	0.999	1.000	1.000	
		PDR	European/Asian	4	365/415	allelic model	1.73 (0.85 , 3.52)	0.134	87.20%	0.155	0.809	0.933	ACB	0.942	0.999	1.000	1.000	Not credible
				4	365/415	dominant model	1.73 (0.76 , 3.95)	0.189	83.83%	0.021	0.704	0.617	BCC	0.952	0.999	1.000	1.000	
				4	365/415	recessive model	2.59 (0.76 , 8.89)	0.130	69.64%	0.953	0.882	0.754	BCB	0.942	0.999	1.000	1.000	
				4	365/415	heterozygous model	1.38 (0.8 , 2.36)	0.245	58.59%	0.183	0.747	0.305	CCB	0.956	0.999	1.000	1.000	
				4	365/415	homozygous model	3.26 (0.73 , 14.57)	0.122	75.06%	0.749	0.915	0.950	ACB	0.940	0.999	1.000	1.000	
			Asian	3	320/354	allelic model	1.23 (0.79 , 1.92)	0.358	59.65%	0.501	0.627	0.219	CCB	0.962	0.999	1.000	1.000	Not credible
				3	320/354	dominant model	1.24 (0.77 , 2)	0.374	52.82%	0.630	0.557	0.196	CCB	0.963	0.999	1.000	1.000	
				3	320/354	recessive model	1.53 (0.39 , 5.92)	0.540	55.23%	0.244	1.000	0.078	CCB	0.965	0.999	1.000	1.000	
				3	320/354	heterozygous model	1.2 (0.77 , 1.88)	0.424	42.19%	0.811	0.450	0.150	CBB	0.964	0.999	1.000	1.000	
				3	320/354	homozygous model	1.66 (0.4 , 6.8)	0.483	57.98%	0.294	1.000	0.109	CCB	0.965	0.999	1.000	1.000	
		NPDR	Asian	3	258/354	allelic model	0.89 (0.62 , 1.29)	0.550	27.07%	0.753	1.000	0.102	CBB	0.956	0.999	1.000	1.000	Not credible
				3	258/354	dominant model	0.85 (0.56 , 1.3)	0.453	29.29%	0.639	1.000	0.137	CBB	0.954	0.999	1.000	1.000	
				3	258/354	recessive model	1.09 (0.44 , 2.71)	0.850	1.06%	0.679	1.000	0.023	CAB	0.965	0.999	1.000	1.000	
				3	258/354	heterozygous model	0.83 (0.54 , 1.28)	0.392	26.15%	0.614	1.000	0.158	CBB	0.952	0.999	1.000	1.000	
				3	258/354	homozygous model	1.03 (0.41 , 2.61)	0.946	2.40%	0.643	1.000	0.017	CAB	0.965	0.999	1.000	1.000	
VEGF rs2010963	C/G	DR	Asian	10	1692/1402	allelic model	1.18 (1 , 1.39)	0.044	55.34%	0.313	0.284	0.795	BCB	0.899	0.998	1.000	1.000	Credible
				10	1692/1402	dominant model	1.29 (1.04 , 1.6)	0.020	43.19%	0.078	0.459	0.816	ABC	0.832	0.996	1.000	1.000	
				10	1692/1402	recessive model	1.22 (0.88 , 1.69)	0.228	57.77%	0.049	0.179	0.349	CCC	0.956	0.999	1.000	1.000	
				10	1692/1402	heterozygous model	1.22 (1 , 1.5)	0.055	30.77%	0.025	1.000	0.569	BBC	0.911	0.998	1.000	1.000	
				10	1692/1402	homozygous model	1.54 (1.08 , 2.21)	0.018	51.63%	0.060	0.302	0.872	ACC	0.825	0.996	1.000	1.000	
		PDR	European/Asian	10	1329/1343	allelic model	1.15 (0.93 , 1.43)	0.193	69.40%	0.020	0.869	0.206	CCC	0.954	0.999	1.000	1.000	Not credible
				10	1329/1343	dominant model	1.12 (0.88 , 1.43)	0.338	48.25%	0.291	0.555	0.121	CBB	0.962	0.999	1.000	1.000	
				10	1329/1343	recessive model	1.35 (0.89 , 2.06)	0.163	68.85%	0.007	0.357	0.193	CCC	0.948	0.999	1.000	1.000	
				10	1329/1343	heterozygous model	1.05 (0.87 , 1.26)	0.619	7.46%	0.357	1.000	0.063	CAB	0.966	0.999	1.000	1.000	
				10	1329/1343	homozygous model	1.41 (0.86 , 2.31)	0.171	71.66%	0.020	0.410	0.182	CCC	0.949	0.999	1.000	1.000	
			European	3	618/368	allelic model	0.93 (0.67 , 1.28)	0.641	63.13%	0.384	0.595	0.236	CCB	0.959	0.999	1.000	1.000	Not credible
				3	618/368	dominant model	0.91 (0.64 , 1.31)	0.620	29.19%	0.964	1.000	0.093	CBB	0.958	0.999	1.000	1.000	
				3	618/368	recessive model	0.89 (0.42 , 1.86)	0.753	69.47%	0.055	0.661	0.327	CCC	0.961	0.999	1.000	1.000	
				3	618/368	heterozygous model	0.99 (0.71 , 1.36)	0.939	11.21%	0.786	1.000	0.025	CAB	0.964	0.999	1.000	1.000	
				3	618/368	homozygous model	0.81 (0.38 , 1.72)	0.586	60.92%	0.307	0.603	0.381	CCB	0.957	0.999	1.000	1.000	
			Asian	7	711/975	allelic model	1.27 (0.99 , 1.63)	0.060	64.41%	0.124	0.953	0.670	BCB	0.912	0.998	1.000	1.000	Credible
				7	711/975	dominant model	1.24 (0.91 , 1.67)	0.173	51.68%	0.130	0.502	0.330	CCB	0.947	0.999	1.000	1.000	
				7	711/975	recessive model	1.62 (1.02 , 2.57)	0.042	59.09%	0.043	0.412	0.684	BCC	0.888	0.998	1.000	1.000	
				7	711/975	heterozygous model	1.1 (0.85 , 1.43)	0.470	28.75%	0.098	1.000	0.088	CBC	0.965	0.999	1.000	1.000	
				7	711/975	homozygous model	1.77 (1.01 , 3.12)	0.048	66.35%	0.063	0.506	0.718	BCC	0.899	0.998	1.000	1.000	
		NPDR	European/Asian	7	641/744	allelic model	1.27 (1.03 , 1.57)	0.025	42.20%	0.218	0.607	0.782	BBB	0.858	0.997	1.000	1.000	Credible
				7	641/744	dominant model	1.28 (0.94 , 1.75)	0.119	41.80%	0.292	0.799	0.475	CBB	0.940	0.999	1.000	1.000	
				7	641/744	recessive model	1.64 (1.06 , 2.54)	0.026	41.05%	0.181	0.596	0.779	BBB	0.857	0.997	1.000	1.000	
				7	641/744	heterozygous model	1.2 (0.89 , 1.61)	0.239	30.92%	0.524	0.651	0.266	CBB	0.955	0.999	1.000	1.000	
				7	641/744	homozygous model	1.8 (1.04 , 3.12)	0.035	51.24%	0.073	0.343	0.703	BCC	0.881	0.997	1.000	1.000	

			Asian	6	571/686	allelic model	1.23 (0.98 , 1.53)	0.075	43.22%	0.445	0.811	0.594	BBB	0.914	0.998	1.000	1.000	Not credible
				6	571/686	dominant model	1.27 (0.91 , 1.79)	0.161	51.06%	0.007	0.806	0.440	CCC	0.949	0.999	1.000	1.000	
				6	571/686	recessive model	1.36 (0.95 , 1.94)	0.095	7.93%	0.190	0.549	0.406	CAB	0.929	0.999	1.000	1.000	
				6	571/686	heterozygous model	1.21 (0.87 , 1.67)	0.259	42.35%	0.012	0.693	0.265	CBC	0.957	0.999	1.000	1.000	
				6	571/686	homozygous model	1.63 (0.93 , 2.85)	0.086	50.88%	0.253	0.516	0.540	BCB	0.928	0.999	1.000	1.000	
VEGF rs3025039	T/C	DR	Asian	4	576/540	allelic model	1.5 (0.96 , 2.36)	0.078	75.15%	0.008	0.286	0.995	ACC	0.924	0.998	1.000	1.000	Credible
				4	576/540	dominant model	1.66 (0.91 , 3)	0.097	79.79%	0.002	0.717	0.997	ACC	0.931	0.999	1.000	1.000	
				3	446/458	recessive model	1.7 (0.65 , 4.48)	0.280	49.12%	0.855	1.000	0.391	CBB	0.959	0.999	1.000	1.000	
				4	576/540	heterozygous model	1.61 (0.87 , 2.98)	0.132	79.93%	0.004	0.686	0.990	ACC	0.942	0.999	1.000	1.000	
				3	446/458	homozygous model	2.34 (1 , 5.44)	0.049	33.16%	0.965	0.724	0.779	BBB	0.899	0.998	1.000	1.000	
		NPDR	Asian	4	320/540	allelic model	1.28 (0.78 , 2.11)	0.333	71.60%	0.023	0.825	0.705	BCC	0.961	0.999	1.000	1.000	Not credible
				4	320/540	dominant model	1.45 (0.76 , 2.8)	0.261	76.87%	0.003	0.886	0.880	ACC	0.958	0.999	1.000	1.000	
				3	234/458	recessive model	0.92 (0.19 , 4.45)	0.917	57.47%	0.496	1.000	0.037	CCB	0.963	0.999	1.000	1.000	
				4	320/540	heterozygous model	1.5 (0.75 , 3.02)	0.256	78.76%	0.004	0.587	0.913	ACC	0.957	0.999	1.000	1.000	
				3	234/458	homozygous model	1.22 (0.28 , 5.21)	0.790	49.42%	0.502	1.000	0.027	CBB	0.966	0.999	1.000	1.000	
VEGF rs699947	A/C	DR	Asian	4	734/767	allelic model	1.29 (0.88 , 1.9)	0.190	80.83%	0.390	0.286	0.776	BCB	0.952	0.999	1.000	1.000	Not credible
				4	734/767	dominant model	1.33 (0.83 , 2.13)	0.243	79.87%	0.619	0.912	0.693	BCB	0.956	0.999	1.000	1.000	
				4	734/767	recessive model	1.46 (0.69 , 3.09)	0.321	68.73%	0.574	0.803	0.401	CCB	0.961	0.999	1.000	1.000	
				4	734/767	heterozygous model	1.26 (0.79 , 2.01)	0.327	77.16%	0.726	0.874	0.502	BCB	0.961	0.999	1.000	1.000	
				4	734/767	homozygous model	1.59 (0.7 , 3.66)	0.271	73.27%	0.530	0.489	0.519	BCB	0.958	0.999	1.000	1.000	
		PDR	European/Asian	3	366/500	allelic model	1.44 (1.11 , 1.85)	0.006	22.23%	0.844	0.416	0.896	AAB	0.606	0.988	0.999	1.000	Credible
				3	366/500	dominant model	1.72 (1.2 , 2.45)	0.003	30.56%	0.628	0.917	0.949	ABB	0.510	0.982	0.998	1.000	
				3	366/500	recessive model	1.04 (0.33 , 3.29)	0.947	59.98%	0.093	1.000	0.144	CCC	0.965	0.999	1.000	1.000	
				3	366/500	heterozygous model	1.75 (1.13 , 2.7)	0.011	47.41%	0.536	0.903	0.926	ABB	0.764	0.994	0.999	1.000	
				3	366/500	homozygous model	1.39 (0.52 , 3.72)	0.516	46.69%	0.185	0.549	0.325	CBB	0.965	0.999	1.000	1.000	
VEGF rs833061	T/C	DR	Asian	4	732/612	allelic model	0.68 (0.38 , 1.24)	0.208	90.01%	0.165	0.132	0.917	ACB	0.949	0.999	1.000	1.000	Not credible
				4	732/612	dominant model	0.5 (0.17 , 1.47)	0.209	72.25%	0.423	0.500	0.565	BCB	0.949	0.999	1.000	1.000	
				4	732/612	recessive model	0.63 (0.28 , 1.41)	0.262	91.79%	0.116	0.850	0.856	ACB	0.949	0.999	1.000	1.000	
				4	732/612	heterozygous model	0.56 (0.23 , 1.34)	0.193	55.11%	0.651	0.322	0.454	CCB	0.948	0.999	1.000	1.000	
				4	732/612	homozygous model	0.44 (0.12 , 1.6)	0.211	80.16%	0.247	0.616	0.623	BCB	0.949	0.999	1.000	1.000	
VEGF rs13207351	A/G	DR	European/Asian	3	294/286	allelic model	1.52 (1.16 , 2)	0.003	9.93%	0.539	0.420	0.880	AAB	0.520	0.983	0.998	1.000	Credible
				3	294/286	dominant model	1.25 (0.87 , 1.8)	0.226	7.97%	0.442	1.000	0.229	CAB	0.955	0.999	1.000	1.000	
				3	294/286	recessive model	2.63 (1.39 , 4.97)	0.003	39.93%	0.348	0.617	0.972	ABB	0.528	0.983	0.998	1.000	
				3	294/286	heterozygous model	0.81 (0.37 , 1.8)	0.611	71.23%	0.072	0.698	0.033	CCC	0.957	0.999	1.000	1.000	
				3	294/286	homozygous model	2.13 (1.09 , 4.14)	0.027	32.64%	0.488	0.762	0.775	BBB	0.854	0.997	1.000	1.000	
ACACB rs2268388	T/C	DKD	European/Asian	12	2921/2909	allelic model	1.46 (1.15 , 1.84)	0.002	82.37%	0.525	0.883	1.000	ACB	0.375	0.969	0.997	1.000	Credible
				12	2921/2909	dominant model	1.44 (1.14 , 1.83)	0.003	75.08%	0.510	0.728	1.000	ACB	0.525	0.983	0.998	1.000	
			Asian	10	2401/2386	allelic model	1.44 (1.09 , 1.91)	0.010	85.36%	0.479	0.909	1.000	ACB	0.763	0.994	0.999	1.000	Credible
				10	2401/2386	dominant model	1.43 (1.07 , 1.89)	0.015	79.32%	0.482	0.788	1.000	ACB	0.770	0.994	0.999	1.000	
		ESRD	European/Asian	3	948/832	allelic model	1.02 (0.65 , 1.6)	0.931	84.68%	0.277	0.829	0.098	CCB	0.965	0.999	1.000	1.000	Not credible
				3	948/832	dominant model	1.03 (0.66 , 1.6)	0.910	78.23%	0.052	0.743	0.125	CCC	0.965	0.999	1.000	1.000	
ADIPOQ rs266729	G/C	DKD	Asian	3	522/506	allelic model	0.89 (0.68 , 1.17)	0.407	40.98%	0.629	0.443	0.193	CBB	0.953	0.999	1.000	1.000	Not credible
ADIPOQ rs1501299	T/G	DKD	Asian	4	473/804	allelic model	0.98 (0.81 , 1.2)	0.866	10.98%	0.411	1.000	0.039	CAB	0.962	0.999	1.000	1.000	Not credible
			European/Asian	9	569/1834	allelic model	1.4 (1.06 , 1.84)	0.017	69.89%	0.909	0.289	0.997	ACB	0.804	0.995	1.000	1.000	Credible
				9	569/1834	dominant model	1.29 (0.93 , 1.78)	0.129	45.51%	0.158	0.567	0.697	BBB	0.940	0.999	1.000	1.000	
				9	569/1834	recessive model	1.84 (1.21 , 2.8)	0.004	62.64%	0.566	0.277	0.999	ACB	0.609	0.988	0.999	1.000	
				9	569/1834	homozygous model	1.97 (1.11 , 3.49)	0.021	68.54%	0.518	0.243	0.997	ACB	0.830	0.996	1.000	1.000	
				9	569/1834	heterozygous model	1.1 (0.86 , 1.4)	0.460	0.00%	0.578	0.410	0.139	CAB	0.964	0.999	1.000	1.000	

ACE Ins/Del	Del/Ins	ESRD	European	3	136/920	allelic model	1.69 (0.88 , 3.25)	0.118	79.94%	0.610	0.567	0.952	ACB	0.938	0.999	1.000	1.000	Not credible
				3	136/920	dominant model	1.93 (0.61 , 6.12)	0.262	71.60%	0.086	0.791	0.501	BCC	0.958	0.999	1.000	1.000	
				3	136/920	recessive model	1.94 (0.99 , 3.78)	0.052	62.73%	0.950	0.471	0.926	ACB	0.903	0.998	1.000	1.000	
				3	136/920	homozygous model	2.61 (0.71 , 9.6)	0.150	74.14%	0.108	0.453	0.882	ACB	0.946	0.999	1.000	1.000	
				3	136/920	heterozygous model	1.41 (0.53 , 3.81)	0.492	56.89%	0.280	0.577	0.106	CCB	0.965	0.999	1.000	1.000	
			Asian	6	433/914	allelic model	1.3 (0.96 , 1.75)	0.090	64.65%	0.397	0.344	0.899	ACB	0.927	0.998	1.000	1.000	Not credible
				6	433/914	dominant model	1.23 (0.94 , 1.61)	0.134	13.85%	0.418	0.550	0.388	CAB	0.942	0.999	1.000	1.000	
				6	433/914	recessive model	1.77 (0.96 , 3.26)	0.066	69.75%	0.560	0.450	0.958	ACB	0.917	0.998	1.000	1.000	
				6	433/914	homozygous model	1.78 (0.91 , 3.45)	0.090	68.76%	0.637	0.402	0.924	ACB	0.928	0.999	1.000	1.000	
				6	433/914	heterozygous model	1.08 (0.83 , 1.41)	0.561	0.00%	0.324	1.000	0.076	CAB	0.966	0.999	1.000	1.000	
AGER rs184003	T/G	DKD	European/Asian	4	542/554	allelic model	1.09 (0.56 , 2.11)	0.804	76.75%	0.959	0.433	0.087	CCB	0.966	0.999	1.000	1.000	Not credible
				4	542/554	dominant model	1.07 (0.53 , 2.15)	0.858	74.59%	0.686	0.391	0.048	CCB	0.965	0.999	1.000	1.000	
				4	542/554	recessive model	1.41 (0.47 , 4.26)	0.545	21.51%	0.691	1.000	0.101	CAB	0.965	0.999	1.000	1.000	
AGER rs2070600	A/G	DKD	European/Asian	4	464/544	allelic model	1 (0.61 , 1.65)	0.999	58.77%	0.100	1.000	0.054	CCC	0.964	0.999	1.000	1.000	Not credible
				4	464/544	dominant model	1.05 (0.6 , 1.85)	0.857	60.50%	0.113	1.000	0.090	CCB	0.965	0.999	1.000	1.000	
				3	266/365	recessive model	0.68 (0.22 , 2.12)	0.504	0.44%	0.773	1.000	0.059	CAB	0.955	0.999	1.000	1.000	
AGER rs1800624	A/T	DKD	European/Asian/ African	5	1091/1002	allelic model	0.92 (0.79 , 1.07)	0.265	10.41%	0.834	1.000	0.216	CAB	0.950	0.999	1.000	1.000	Not credible
				4	833/807	dominant model	0.86 (0.69 , 1.06)	0.146	3.35%	0.989	1.000	0.302	CAB	0.948	0.999	1.000	1.000	
				4	833/807	recessive model	0.7 (0.42 , 1.15)	0.160	26.47%	0.692	1.000	0.403	CBB	0.948	0.999	1.000	1.000	
AGER rs1800625	C/T	DKD	European/Asian/ African	5	1019/792	allelic model	1.07 (0.78 , 1.48)	0.662	61.92%	0.854	0.336	0.151	CCB	0.966	0.999	1.000	1.000	Not credible
				4	761/597	dominant model	0.95 (0.6 , 1.5)	0.823	67.80%	0.713	0.718	0.053	CCB	0.962	0.999	1.000	1.000	
				4	761/597	recessive model	2.34 (0.78 , 6.98)	0.127	41.81%	0.235	0.261	0.775	BBB	0.941	0.999	1.000	1.000	
AGT rs699 M235T	C/T	DKD	European/Asian	11	2228/2039	allelic model	1.19 (0.93 , 1.51)	0.172	83.51%	0.583	0.919	0.715	BCB	0.946	0.999	1.000	1.000	Not credible
				11	2228/2039	dominant model	1.15 (0.85 , 1.54)	0.361	71.72%	0.326	0.660	0.089	CCB	0.962	0.999	1.000	1.000	
				11	2228/2039	recessive model	1.39 (0.96 , 2.02)	0.080	77.77%	0.890	0.883	0.957	ACB	0.927	0.998	1.000	1.000	
				11	2228/2039	heterozygous model	1.01 (0.79 , 1.29)	0.917	53.18%	0.462	0.608	0.082	CCB	0.965	0.999	1.000	1.000	
				11	2228/2039	homozygous model	1.5 (0.96 , 2.35)	0.076	75.61%	0.740	0.960	0.964	ACB	0.923	0.998	1.000	1.000	
AGTR1 rs5186	C/A	DKD	Asian	3	1176/1035	allelic model	1.25 (0.98 , 1.59)	0.071	34.60%	0.395	0.678	0.686	BBB	0.918	0.998	1.000	1.000	Not credible
				4	1921/1442	dominant model	1.12 (0.9 , 1.39)	0.313	31.44%	0.868	1.000	0.222	CBB	0.960	0.999	1.000	1.000	
				3	1176/1035	recessive model	1.49 (0.26 , 8.64)	0.659	68.79%	0.028	0.693	0.796	BCC	0.966	0.999	1.000	1.000	
				3	1176/1035	heterozygous model	1.18 (0.94 , 1.47)	0.150	4.29%	0.501	1.000	0.301	CAB	0.944	0.999	1.000	1.000	
				3	1176/1035	homozygous model	1.54 (0.26 , 9.22)	0.638	69.62%	0.023	0.702	0.818	ACC	0.966	0.999	1.000	1.000	
			European/Asian	4	1294/1152	allelic model	1.24 (1.01 , 1.51)	0.037	22.39%	0.334	0.704	0.711	BAB	0.872	0.997	1.000	1.000	Credible
				5	2039/1559	dominant model	1.17 (0.96 , 1.41)	0.112	20.81%	0.585	1.000	0.401	CAB	0.933	0.999	1.000	1.000	
				4	1294/1152	recessive model	1.01 (0.25 , 4.12)	0.984	73.63%	0.399	0.787	0.267	CCB	0.964	0.999	1.000	1.000	
				4	1294/1152	heterozygous model	1.29 (0.95 , 1.74)	0.100	48.98%	0.695	0.770	0.619	BBB	0.932	0.999	1.000	1.000	
				4	1294/1152	homozygous model	1.14 (0.29 , 4.51)	0.853	71.99%	0.318	0.772	0.399	CCB	0.965	0.999	1.000	1.000	
ALR (AC)n	NA	DKD	European/Asian	9	1470/1359	allele (Z-2), present vs null	1.08 (0.96 , 1.22)	0.190	0.00%	0.193	0.491	0.255	CAB	0.954	0.999	1.000	1.000	Not credible
				7	1231/1204	allele (Z+2), present vs null	0.88 (0.67 , 1.15)	0.351	61.54%	0.830	0.875	0.283	CCB	0.951	0.999	1.000	1.000	
			European/Asian	33	3268/3254	allelic model (ε2 vs ε3)	1.72 (1.24 , 2.39)	0.001	81.39%	0.257	0.301	1.000	ACB	0.358	0.967	0.997	1.000	Credible
				33	3268/3254	allelic model (ε4 vs ε3)	0.97 (0.77 , 1.21)	0.767	64.87%	0.959	0.579	0.038	CCB	0.961	0.999	1.000	1.000	
				33	3268/3254	ε2/ε3 vs ε3/ε3	1.71 (1.2 , 2.43)	0.003	72.04%	0.149	0.268	0.972	ACB	0.518	0.983	0.998	1.000	
				31	3200/3212	ε3/ε4 vs ε3/ε3	0.9 (0.66 , 1.22)	0.490	69.63%	0.719	0.541	0.020	CCB	0.955	0.999	1.000	1.000	
				24	2635/2799	ε2/ε2 vs ε3/ε3	1.8 (1.12 , 2.9)	0.015	3.61%	0.844	0.638	0.984	AAB	0.804	0.995	1.000	1.000	
				27	2942/2891	ε2/ε4 vs ε3/ε3	1.27 (0.77 , 2.09)	0.353	23.89%	0.881	0.509	0.706	BAB	0.962	0.999	1.000	1.000	
				22	2278/2459	ε4/ε4 vs ε3/ε3	0.96 (0.62 , 1.48)	0.846	0.00%	0.781	0.391	0.192	CAB	0.962	0.999	1.000	1.000	
				19	2296/1857	allelic model (ε2 vs ε3)	1.87 (1.19 , 2.94)	0.006	85.88%	0.019	0.589	0.985	ACC	0.683	0.991	0.999	1.000	

APOE ε2/ε3/ε4	NA	DKD	Asian (Chinese)	19	2296/1857	allelic model (ε4 vs ε3)	1.24 (0.92 , 1.67)	0.159	66.82%	0.776	0.911	0.640	BCB	0.947	0.999	1.000	1.000	Credible
				19	2296/1857	ε2/ε3 vs ε3/ε3	1.73 (1.08 , 2.76)	0.022	75.30%	0.200	0.651	0.626	BCB	0.837	0.996	1.000	1.000	
				17	2228/1815	ε3/ε4 vs ε3/ε3	1.25 (0.81 , 1.93)	0.319	73.46%	0.703	0.830	0.968	ACB	0.960	0.999	1.000	1.000	
				16	1975/1756	ε2/ε2 vs ε3/ε3	2.05 (1.04 , 4.06)	0.039	26.80%	0.893	0.745	0.988	ABB	0.887	0.998	1.000	1.000	
				19	2296/1857	ε2/ε4 vs ε3/ε3	1.35 (0.7 , 2.61)	0.371	41.87%	0.867	0.778	0.618	BBB	0.962	0.999	1.000	1.000	
				12	1551/1363	ε4/ε4 vs ε3/ε3	0.78 (0.45 , 1.37)	0.387	0.00%	0.427	0.278	0.133	CAB	0.952	0.999	1.000	1.000	
			European/Asian (non-Chinese)	14	972/1397	allelic model (ε2 vs ε3)	1.54 (0.96 , 2.48)	0.074	69.75%	0.486	0.265	0.946	ACB	0.922	0.998	1.000	1.000	Credible
				14	972/1397	allelic model (ε4 vs ε3)	0.7 (0.52 , 0.93)	0.014	46.21%	0.541	0.420	0.922	ABB	0.942	0.999	1.000	1.000	
				14	972/1397	ε2/ε3 vs ε3/ε3	1.69 (0.95 , 2.99)	0.073	68.71%	0.494	0.241	0.928	ACB	0.920	0.998	1.000	1.000	
				14	972/1397	ε3/ε4 vs ε3/ε3	0.61 (0.45 , 0.83)	0.002	32.87%	0.679	0.405	0.998	ABB	0.855	0.997	1.000	1.000	
				8	660/1043	ε2/ε2 vs ε3/ε3	1.48 (0.62 , 3.54)	0.378	0.00%	0.724	1.000	0.076	CAB	0.963	0.999	1.000	1.000	
				8	646/1034	ε2/ε4 vs ε3/ε3	1.1 (0.46 , 2.59)	0.834	0.00%	0.874	0.515	0.103	CAB	0.966	0.999	1.000	1.000	
				10	727/1096	ε4/ε4 vs ε3/ε3	1.31 (0.66 , 2.63)	0.444	0.00%	0.491	1.000	0.065	CAB	0.964	0.999	1.000	1.000	
AKR1B1 rs759853	T/C	DKD	Asian	3	708/1552	allelic model	1.24 (1.06 , 1.46)	0.009	3.16%	0.593	1.000	0.740	BAB	0.743	0.993	0.999	1.000	Credible
				3	708/1552	dominant model	1.28 (1.06 , 1.55)	0.012	0.20%	0.782	1.000	0.692	BAB	0.764	0.994	0.999	1.000	
				3	708/1552	recessive model	1.55 (0.63 , 3.83)	0.343	73.81%	0.047	0.778	0.262	CCC	0.961	0.999	1.000	1.000	
				3	708/1552	homozygous model	1.65 (0.7 , 3.91)	0.252	70.22%	0.061	0.777	0.394	CCC	0.957	0.999	1.000	1.000	
				3	708/1552	codominant model	1.21 (0.97 , 1.51)	0.092	20.27%	0.252	1.000	0.474	CAB	0.930	0.999	1.000	1.000	
			European/Asian/ Pima Indian	8	1376/2187	allelic model	1.26 (1.1 , 1.44)	0.001	15.86%	0.095	0.708	0.950	AAC	0.257	0.948	0.995	1.000	Credible
				8	1376/2187	dominant model	1.35 (1.13 , 1.62)	0.001	26.63%	0.063	0.765	0.966	ABC	0.361	0.967	0.997	1.000	
				8	1376/2187	recessive model	1.26 (0.85 , 1.85)	0.249	33.57%	0.394	0.724	0.256	CBB	0.956	0.999	1.000	1.000	
				8	1376/2187	homozygous model	1.51 (0.98 , 2.32)	0.060	38.50%	0.445	0.539	0.632	BBB	0.911	0.998	1.000	1.000	
				8	1376/2187	codominant model	1.29 (1.08 , 1.54)	0.006	23.81%	0.156	0.898	0.876	AAB	0.626	0.989	0.999	1.000	
CCL5 rs2280788	G/C	DKD	Asian	3	508/659	dominant model	1.02 (0.79 , 1.33)	0.866	1.36%	0.724	1.000	0.032	CAB	0.965	0.999	1.000	1.000	Not credible
CCL5 rs2107538	A/G	Microvascular complications	Asian	3	530/624	dominant model	0.96 (0.69 , 1.33)	0.796	38.39%	0.319	1.000	0.069	CBB	0.961	0.999	1.000	1.000	Not credible
CCR5 rs1799987	A/G	Microalbuminuria	Asian	3	281/471	dominant model	1.68 (1.15 , 2.44)	0.007	0.21%	0.968	0.741	0.758	BAB	0.676	0.991	0.999	1.000	Credible
		Macroalbuminuria	European/Asian	4	890/928	dominant model	4.18 (1.44 , 12.13)	0.009	90.19%	0.073	0.694	1.000	ACC	0.721	0.993	0.999	1.000	Credible
CYP11B2 rs1799998	T/C	DKD	Asian	4	403/431	allelic model	1.41 (1.02 , 1.94)	0.038	49.03%	0.519	0.472	0.924	ABB	0.878	0.997	1.000	1.000	Credible
				4	403/431	dominant model	1.75 (0.91 , 3.36)	0.094	34.85%	0.802	0.699	0.633	BBB	0.931	0.999	1.000	1.000	
				4	403/431	recessive model	1.49 (1.05 , 2.12)	0.026	29.46%	0.283	0.789	0.852	ABB	0.857	0.997	1.000	1.000	
				4	403/431	heterozygous model	1.45 (0.78 , 2.69)	0.241	23.03%	0.908	1.000	0.253	CAB	0.956	0.999	1.000	1.000	
				4	403/431	homozygous model	2.02 (1 , 4.08)	0.051	39.02%	0.588	0.354	0.828	ABB	0.901	0.998	1.000	1.000	
eNOS rs1799983	T/G	DKD	Asian	5	872/916	allelic model	1.33 (0.66 , 2.67)	0.430	89.43%	0.152	0.539	0.025	CCB	0.964	0.999	1.000	1.000	Not credible
				5	872/916	dominant model	1.41 (0.66 , 3.03)	0.372	89.03%	0.095	0.545	0.029	CCC	0.963	0.999	1.000	1.000	
				4	769/857	recessive model	1 (0.3 , 3.33)	0.999	47.30%	0.295	0.518	0.076	CBB	0.964	0.999	1.000	1.000	
				5	872/916	codominant model	1.39 (0.7 , 2.76)	0.350	85.90%	0.073	0.457	0.048	CCC	0.962	0.999	1.000	1.000	
			European/Asian	16	3030/3136	allelic model	1.27 (1.02 , 1.58)	0.033	82.53%	0.230	0.073	0.998	ACB	0.871	0.997	1.000	1.000	Credible
				16	3030/3136	dominant model	1.33 (1.03 , 1.72)	0.032	79.30%	0.182	0.469	0.975	ACB	0.866	0.997	1.000	1.000	
				16	2994/3240	recessive model	1.26 (0.91 , 1.75)	0.164	58.28%	0.353	0.031	0.936	ACB	0.949	0.999	1.000	1.000	
				16	3030/3136	codominant model	1.13 (0.93 , 1.37)	0.212	61.34%	0.296	0.764	0.336	CCB	0.954	0.999	1.000	1.000	
eNOS rs2070744	C/T	DKD	European/Asian	7	2186/2163	allelic model	1.27 (1.09 , 1.48)	0.002	57.45%	0.585	0.908	0.995	ACB	0.472	0.979	0.998	1.000	Credible
				7	2186/2163	dominant model	1.29 (1.1 , 1.52)	0.002	39.28%	0.767	0.794	0.980	ABB	0.485	0.980	0.998	1.000	
				7	2186/2163	recessive model	1.72 (1.06 , 2.77)	0.028	71.75%	0.008	0.630	0.877	ACC	0.853	0.997	1.000	1.000	
				7	2186/2163	codominant model	1.16 (1.02 , 1.31)	0.022	0.00%	0.192	0.254	0.619	BAB	0.811	0.996	1.000	1.000	
			Asian	10	1214/1565	allelic model	1.11 (0.86 , 1.43)	0.434	45.62%	0.969	0.501	0.087	CBB	0.964	0.999	1.000	1.000	Not credible
				10	1214/1565	dominant model	1.08 (0.83 , 1.41)	0.565	40.38%	0.784	0.416	0.061	CBB	0.966	0.999	1.000	1.000	
				8	1033/1436	recessive model	1.29 (0.67 , 2.48)	0.453	0.00%	0.612	1.000	0.091	CAB	0.964	0.999	1.000	1.000	
eNOS rs869109213	4a/4b	DKD		10	1214/1565	codominant model	1.03 (0.81 , 1.32)	0.790	29.03%	0.632	0.670	0.036	CBB	0.966	0.999	1.000	1.000	

			European/Asian	16	2959/3373	allelic model	1.19 (0.99 , 1.43)	0.063	65.30%	0.691	0.873	0.934	ACB	0.914	0.998	1.000	1.000	Credible
				16	2959/3373	dominant model	1.11 (0.95 , 1.29)	0.201	36.89%	0.831	0.392	0.340	CBB	0.950	0.999	1.000	1.000	
				14	2778/3244	recessive model	1.85 (1.05 , 3.27)	0.035	67.38%	0.739	0.965	1.000	ACB	0.877	0.997	1.000	1.000	
				16	2959/3373	codominant model	0.96 (0.82 , 1.13)	0.620	37.43%	0.422	0.613	0.152	CBB	0.958	0.999	1.000	1.000	
GLUT1 XbαI	1.1/0.9	DKD	European/Asian	3	216/307	allelic model	1.18 (0.65 , 2.13)	0.581	73.55%	0.151	0.273	0.027	CCB	0.966	0.999	1.000	1.000	Not credible
				3	216/307	dominant model	1.51 (0.47 , 4.8)	0.487	85.17%	0.236	0.482	0.021	CCB	0.965	0.999	1.000	1.000	
				3	216/307	recessive model	0.94 (0.5 , 1.76)	0.856	1.03%	0.722	1.000	0.029	CAB	0.962	0.999	1.000	1.000	
				3	216/307	homozygous model	1.11 (0.43 , 2.82)	0.831	37.88%	0.081	1.000	0.033	CBC	0.966	0.999	1.000	1.000	
GSTM1	NA	DKD	European/Asian	6	448/480	Gene (GSTM1), null vs present	1.22 (0.83 , 1.8)	0.305	46.96%	0.860	0.723	0.299	CBB	0.960	0.999	1.000	1.000	Not credible
GSTT1	NA	DKD	European/Asian	4	259/288	Gene (GSTT1), null vs present	1.92 (1.09 , 3.38)	0.024	57.81%	0.524	0.610	0.926	ACB	0.846	0.997	1.000	1.000	Credible
ICAM1 rs5498	G/A	DKD	Asian	4	909/857	allelic model	0.85 (0.55 , 1.31)	0.456	88.52%	0.610	0.068	0.849	ACB	0.954	0.999	1.000	1.000	Not credible
				4	909/857	dominant model	1.11 (0.53 , 2.32)	0.783	84.29%	0.242	0.587	0.200	CCB	0.966	0.999	1.000	1.000	
				4	909/857	recessive model	0.61 (0.3 , 1.2)	0.153	83.45%	0.506	0.353	0.991	ACB	0.948	0.999	1.000	1.000	
				4	909/857	homozygous model	0.78 (0.3 , 2)	0.606	83.50%	0.547	0.220	0.499	CCB	0.957	0.999	1.000	1.000	
IL-6 rs1800795	C/G	DKD	European/Asian	8	1203/1079	allelic model	0.82 (0.7 , 0.97)	0.019	20.33%	0.145	0.496	0.769	BAB	0.947	0.999	1.000	1.000	Credible
				8	1203/1079	dominant model	0.8 (0.66 , 0.99)	0.037	16.65%	0.321	0.775	0.638	BAB	0.950	0.999	1.000	1.000	
				7	1133/1019	recessive model	0.81 (0.62 , 1.06)	0.120	0.00%	0.415	1.000	0.411	CAB	0.949	0.999	1.000	1.000	
				8	1203/1079	heterozygous model	0.84 (0.65 , 1.08)	0.175	32.60%	0.492	1.000	0.387	CBB	0.948	0.999	1.000	1.000	
				7	1133/1019	homozygous model	0.79 (0.6 , 1.04)	0.097	0.00%	0.394	1.000	0.450	CAB	0.949	0.999	1.000	1.000	
		Microalbuminuria	European	4	238/427	allelic model	0.78 (0.48 , 1.25)	0.300	61.42%	0.404	0.762	0.275	CCB	0.950	0.999	1.000	1.000	Not credible
				4	238/427	dominant model	0.86 (0.49 , 1.48)	0.577	52.67%	0.833	1.000	0.074	CCB	0.957	0.999	1.000	1.000	
				3	168/367	recessive model	0.57 (0.12 , 2.63)	0.474	79.54%	0.228	1.000	0.364	CCB	0.954	0.999	1.000	1.000	
				4	238/427	heterozygous model	1.01 (0.57 , 1.78)	0.974	49.34%	0.764	1.000	0.040	CBB	0.964	0.999	1.000	1.000	
				3	168/367	homozygous model	0.63 (0.11 , 3.49)	0.595	80.00%	0.254	1.000	0.187	CCB	0.957	0.999	1.000	1.000	
		Macroalbuminuria	European	3	235/492	allelic model	0.89 (0.69 , 1.14)	0.365	8.52%	0.460	1.000	0.147	CAB	0.951	0.999	1.000	1.000	Not credible
				3	235/492	dominant model	0.92 (0.65 , 1.3)	0.634	7.96%	0.397	1.000	0.065	CAB	0.958	0.999	1.000	1.000	
				3	235/492	recessive model	0.79 (0.5 , 1.23)	0.296	2.05%	0.584	1.000	0.153	CAB	0.950	0.999	1.000	1.000	
				3	235/492	heterozygous model	1 (0.69 , 1.46)	0.996	8.06%	0.372	1.000	0.022	CAB	0.964	0.999	1.000	1.000	
				3	235/492	homozygous model	0.8 (0.49 , 1.3)	0.358	4.92%	0.479	1.000	0.129	CAB	0.952	0.999	1.000	1.000	
IL-6 rs1800796	G/C	DKD	European/Asian	4	762/509	allelic model	1.53 (1.15 , 2.03)	0.003	38.59%	0.511	0.271	0.980	ABB	0.547	0.985	0.998	1.000	Credible
				4	762/509	dominant model	1.5 (1.09 , 2.06)	0.012	16.25%	0.729	0.353	0.812	AAB	0.773	0.994	0.999	1.000	
				4	762/509	recessive model	2.12 (1.11 , 4.05)	0.023	51.19%	0.164	0.551	0.899	ACB	0.843	0.996	1.000	1.000	
		Microalbuminuria	Asian	3	316/339	allelic model	1.34 (1.01 , 1.76)	0.042	3.76%	0.579	1.000	0.522	BAB	0.879	0.997	1.000	1.000	Credible
				3	316/339	dominant model	1.26 (0.85 , 1.88)	0.250	30.88%	0.773	1.000	0.257	CBB	0.957	0.999	1.000	1.000	
				3	316/339	recessive model	2.06 (1.02 , 4.15)	0.044	5.83%	0.462	0.553	0.528	BAB	0.892	0.998	1.000	1.000	
		Macroalbuminuria	European/Asian	4	446/509	allelic model	1.8 (1.23 , 2.64)	0.003	59.54%	0.628	0.476	0.999	ACB	0.508	0.982	0.998	1.000	Credible
				4	446/509	dominant model	1.83 (1.29 , 2.62)	0.001	12.26%	0.628	0.587	0.954	AAB	0.313	0.960	0.996	1.000	
				4	446/509	recessive model	2.78 (1.24 , 6.26)	0.013	65.88%	0.079	0.361	0.962	ACC	0.786	0.995	0.999	1.000	
IL-10 rs1800896	G/A	DKD	European/Asian	4	626/532	allelic model	1.03 (0.78 , 1.36)	0.839	30.00%	0.396	1.000	0.211	CBB	0.966	0.999	1.000	1.000	Not credible
				4	626/532	dominant model	1 (0.45 , 2.23)	0.998	64.99%	0.576	1.000	0.162	CCB	0.964	0.999	1.000	1.000	
IL-10 rs1800872	A/C	DKD	Asian/African	4	739/625	allelic model	1.09 (0.86 , 1.39)	0.471	33.39%	0.181	1.000	0.041	CBB	0.965	0.999	1.000	1.000	Not credible
				4	739/625	dominant model	0.97 (0.36 , 2.61)	0.959	87.47%	0.242	1.000	0.044	CCB	0.964	0.999	1.000	1.000	
				4	739/625	recessive model	1.59 (0.51 , 4.96)	0.425	86.40%	0.068	0.932	0.141	CCC	0.964	0.999	1.000	1.000	
MCP-1 rs1024611	G/A	DKD	Asian	8	908/860	allelic model	0.89 (0.65 , 1.22)	0.467	79.37%	0.917	0.629	0.280	CCB	0.954	0.999	1.000	1.000	Credible
				8	908/860	dominant model	1.04 (0.74 , 1.48)	0.810	55.07%	0.706	0.468	0.136	CCB	0.966	0.999	1.000	1.000	
				7	865/817	recessive model	0.66 (0.39 , 1.13)	0.133	80.53%	0.623	0.687	0.901	ACB	0.948	0.999	1.000	1.000	
				8	908/860	codominant model	1.39 (1.04 , 1.87)	0.026	51.64%	0.464	0.744	0.945	ACB	0.865	0.997	1.000	1.000	

MCP-1 rs1024611	G/A	DKD	Asian (Chinese)	4	349/281	allelic model	0.85 (0.39 , 1.87)	0.694	91.41%	0.462	0.787	0.183	CCB	0.959	0.999	1.000	1.000	Not credible
				4	349/281	dominant model	1.07 (0.5 , 2.27)	0.864	76.76%	0.568	0.430	0.029	CCB	0.965	0.999	1.000	1.000	
				4	349/281	recessive model	0.7 (0.24 , 2.06)	0.515	88.97%	0.452	0.954	0.469	CCB	0.955	0.999	1.000	1.000	
				4	349/281	codominant model	1.49 (0.79 , 2.8)	0.213	70.64%	0.006	0.850	0.480	CCC	0.954	0.999	1.000	1.000	
MTHFR rs1801133	T/C	DKD	European/Asian	24	3205/3185	allelic model	1.6 (1.3 , 1.97)	1.18E-05	85.50%	0.266	0.175	1.000	ACB	0.008	0.304	0.814	0.998	Credible
				24	3205/3185	dominant model	1.82 (1.36 , 2.45)	6.67E-05	85.24%	0.023	0.364	1.000	ACC	0.051	0.739	0.966	1.000	
				22	3047/3055	recessive model	1.81 (1.43 , 2.28)	5.99E-07	54.51%	0.487	0.253	1.000	ACB	5.47E-04	0.028	0.224	0.966	
				24	3205/3185	heterozygous model	1.66 (1.25 , 2.21)	0.001	81.99%	0.009	0.584	1.000	ACC	0.213	0.934	0.993	1.000	
				22	3047/3055	homozygous model	2.43 (1.71 , 3.45)	7.26E-07	75.00%	0.581	0.159	1.000	ACB	7.70E-04	0.039	0.289	0.976	
			European	11	1692/1655	allelic model	1.7 (1.18 , 2.47)	0.005	89.86%	0.523	0.802	1.000	ACB	0.645	0.990	0.999	1.000	Credible
				11	1692/1655	dominant model	2 (1.23 , 3.24)	0.005	89.41%	0.142	0.920	1.000	ACB	0.627	0.989	0.999	1.000	
				10	1670/1625	recessive model	1.93 (1.21 , 3.07)	0.005	68.22%	0.756	0.636	1.000	ACB	0.649	0.990	0.999	1.000	
				11	1692/1655	heterozygous model	1.83 (1.16 , 2.91)	0.010	86.80%	0.085	0.950	1.000	ACC	0.754	0.994	0.999	1.000	
				10	1670/1625	homozygous model	2.54 (1.28 , 5.02)	0.007	83.14%	0.549	0.676	1.000	ACB	0.698	0.992	0.999	1.000	
NADPH rs4673	T/C	DKD	European/Asian	7	1068/1027	allelic model	1.2 (0.87 , 1.65)	0.271	65.92%	0.035	0.305	0.744	BCC	0.958	0.999	1.000	1.000	Credible
				7	1068/1027	dominant model	1.17 (0.78 , 1.75)	0.449	68.70%	0.033	0.306	0.586	BCC	0.964	0.999	1.000	1.000	
				6	993/965	recessive model	1.44 (0.98 , 2.11)	0.064	0.00%	0.724	1.000	0.466	CAB	0.912	0.998	1.000	1.000	
				6	993/965	homozygous model	1.59 (1.06 , 2.36)	0.023	0.00%	0.832	1.000	0.630	BAB	0.836	0.996	1.000	1.000	
			Asian	6	802/831	allelic model	1.14 (0.74 , 1.77)	0.546	71.58%	0.026	0.246	0.556	BCC	0.965	0.999	1.000	1.000	Credible
				6	802/831	dominant model	1.08 (0.64 , 1.83)	0.762	73.47%	0.044	0.257	0.303	CCC	0.966	0.999	1.000	1.000	
				5	727/769	recessive model	1.76 (1.03 , 3)	0.038	0.00%	0.860	1.000	0.554	BAB	0.884	0.997	1.000	1.000	
				5	727/769	homozygous model	1.79 (1.04 , 3.08)	0.036	0.00%	0.929	1.000	0.565	BAB	0.879	0.997	1.000	1.000	
PPARγ rs1801282	G/C	DKD	European/Asian	19	4408/4409	dominant model	0.69 (0.55 , 0.87)	0.002	52.58%	0.081	0.634	0.995	ACB	0.857	0.997	1.000	1.000	Credible
			European	8	1223/2650	dominant model	0.59 (0.40 , 0.87)	0.008	64.96%	0.001	0.711	0.959	ACC	0.931	0.999	1.000	1.000	Credible
			Asian	11	3185/1759	dominant model	0.76 (0.56 , 1.03)	0.072	44.28%	0.990	0.693	0.756	BBB	0.950	0.999	1.000	1.000	Not credible
SERPINE1 rs1799889	4G/5G	DKD	Asian	11	940/675	allelic model	1.7 (1.17 , 2.47)	0.005	84.12%	0.966	0.856	1.000	ACB	0.645	0.990	0.999	1.000	Credible
				11	940/675	dominant model	1.59 (0.94 , 2.69)	0.084	74.84%	0.649	0.850	0.993	ACB	0.927	0.998	1.000	1.000	
				11	940/675	recessive model	2.24 (1.4 , 3.59)	0.001	74.97%	0.065	0.339	1.000	ACC	0.281	0.954	0.995	1.000	
				11	940/675	heterozygous model	1.15 (0.71 , 1.86)	0.560	64.96%	0.677	0.539	0.281	CCB	0.966	0.999	1.000	1.000	
				11	940/675	homozygous model	2.46 (1.3 , 4.65)	0.006	76.29%	0.832	0.858	1.000	ACB	0.652	0.990	0.999	1.000	
TCF7L2 rs7903146	T/C	DKD	Asian	5	966/1712	allelic model	1.22 (0.87 , 1.71)	0.252	70.59%	0.416	0.564	0.971	ACB	0.957	0.999	1.000	1.000	Credible
				5	966/1712	dominant model	1.23 (0.86 , 1.75)	0.266	63.71%	0.477	0.840	0.888	ACB	0.957	0.999	1.000	1.000	
				5	966/1712	recessive model	1.9 (1.04 , 3.48)	0.038	22.33%	0.187	0.343	0.859	AAB	0.883	0.997	1.000	1.000	
				5	966/1712	heterozygous model	1.19 (0.88 , 1.61)	0.253	44.98%	0.332	0.703	0.673	BBB	0.958	0.999	1.000	1.000	
				5	966/1712	homozygous model	2.02 (1 , 4.1)	0.051	32.28%	0.122	0.388	0.938	ABB	0.903	0.998	1.000	1.000	
TGF-β1 rs1800470	C/T	DKD	Asian	7	786/815	allelic model	1.36 (1.05 , 1.77)	0.020	65.46%	0.406	0.088	0.995	ACB	0.840	0.996	1.000	1.000	Credible
				7	786/815	dominant model	1.91 (0.94 , 3.87)	0.073	80.57%	0.919	0.160	0.992	ACB	0.920	0.998	1.000	1.000	
				7	786/815	recessive model	1.39 (1.13 , 1.71)	0.002	0.00%	0.450	0.829	0.864	AAB	0.436	0.976	0.998	1.000	
TLR4 rs4986790	G/A	DKD	European/Asian	3	427/1130	allelic model	1.14 (0.63 , 2.06)	0.669	29.06%	0.459	1.000	0.115	CBB	0.966	0.999	1.000	1.000	Not credible
				3	427/1130	dominant model	1.13 (0.6 , 2.15)	0.703	32.18%	0.438	1.000	0.110	CBB	0.966	0.999	1.000	1.000	
TNF-α rs1800629	A/G	DKD	European/Asian	10	1926/1859	allelic model	1.13 (0.83 , 1.53)	0.443	75.91%	0.114	0.636	0.989	ACB	0.964	0.999	1.000	1.000	Not credible
				9	1902/1836	dominant model	1.15 (0.79 , 1.67)	0.470	78.64%	0.174	0.641	0.981	ACB	0.964	0.999	1.000	1.000	
				8	1780/1711	recessive model	1.32 (0.8 , 2.19)	0.277	37.22%	0.476	0.771	0.560	BBB	0.959	0.999	1.000	1.000	
				9	1902/1836	heterozygous model	1.13 (0.78 , 1.65)	0.520	75.79%	0.253	0.840	0.939	ACB	0.965	0.999	1.000	1.000	
				8	1780/1711	homozygous model	1.43 (0.78 , 2.62)	0.243	51.74%	0.403	0.870	0.795	BCB	0.957	0.999	1.000	1.000	
			Asian	7	1465/1351	allelic model	1.15 (0.78 , 1.67)	0.482	77.50%	0.002	0.421	1.000	ACC	0.964	0.999	1.000	1.000	
				6	1441/1328	dominant model	1.15 (0.7 , 1.9)	0.587	83.15%	0.001	0.456	0.998	ACC	0.966	0.999	1.000	1.000	
				5	1319/1203	recessive model	1.69 (1.17 , 2.42)	0.005	0.00%	0.623	0.790	0.810	AAB	0.599	0.987	0.999	1.000	Credible

				6	1441/1328	heterozygous model	1.11 (0.66 , 1.87)	0.696	82.10%	0.000	0.759	0.983	ACC	0.966	0.999	1.000	1.000	
				5	1319/1203	homozygous model	1.99 (1.31 , 3.02)	0.001	8.15%	0.412	0.920	0.956	AAB	0.357	0.967	0.997	1.000	
MTHFR rs1801133	T/C	DPN	European/Asian	3	138/84	allelic model	1.23 (0.8 , 1.88)	0.354	7.39%	0.921	1.000	0.142	CAB	0.961	0.999	1.000	1.000	Not credible
				3	138/84	dominant model	1.4 (0.77 , 2.57)	0.269	3.23%	0.909	1.000	0.164	CAB	0.959	0.999	1.000	1.000	
				3	138/84	heterozygous model	1.39 (0.75 , 2.58)	0.294	1.13%	0.976	1.000	0.145	CAB	0.960	0.999	1.000	1.000	
TLR4 rs4986790	G/A	DPN	European	3	463/1099	allelic model	1.39 (0.81 , 2.36)	0.231	29.82%	0.668	0.548	0.209	CBB	0.955	0.999	1.000	1.000	Not credible
				3	463/1099	dominant model	1.4 (0.79 , 2.5)	0.252	33.40%	0.679	0.557	0.186	CBB	0.957	0.999	1.000	1.000	
eNOS rs1799983	T/G	DF	European/Asian	3	269/305	allelic model	0.83 (0.47 , 1.45)	0.510	72.21%	0.565	0.725	0.491	CCB	0.955	0.999	1.000	1.000	Not credible
				4	331/340	dominant model	0.81 (0.49 , 1.34)	0.420	55.31%	0.972	0.650	0.248	CCB	0.953	0.999	1.000	1.000	
				3	269/305	recessive model	0.64 (0.14 , 3)	0.573	57.42%	0.100	0.597	0.624	BCC	0.957	0.999	1.000	1.000	
MMP-9 rs3918242	T/C	DF	Asian	3	285/671	allelic model	1.3 (0.8 , 2.1)	0.292	72.33%	0.030	0.778	0.273	CCC	0.959	0.999	1.000	1.000	Not credible
				3	285/671	dominant model	1.36 (0.82 , 2.25)	0.231	63.96%	0.056	0.730	0.334	CCC	0.956	0.999	1.000	1.000	
				3	285/671	recessive model	1.15 (0.37 , 3.53)	0.809	37.42%	0.168	1.000	0.025	CBB	0.966	0.999	1.000	1.000	
CCR5 rs1799987	A/G	Microvascular complications	European/Asian	7	1192/1436	dominant model	2.62 (1.57 , 4.36)	2.20E-04	85.08%	0.854	0.905	1.000	ACB	0.112	0.869	0.985	1.000	Credible
			Asian	6	996/1163	dominant model	2.08 (1.68 , 2.57)	1.2E-11	0.00%	0.736	0.946	1.000	AAB	2.62E-08	1.38E-06	1.38E-05	0.001	Credible
eNOS rs869109213	4a/4b	Microvascular complications	Asian	13	1481/1225	allelic model	1.36 (0.91 , 2.05)	0.138	76.83%	0.270	0.159	0.924	ACB	0.944	0.999	1.000	1.000	Not credible
				13	1481/1225	dominant model	1.36 (0.9 , 2.06)	0.144	72.51%	0.304	0.096	0.841	ACB	0.945	0.999	1.000	1.000	
				10	1238/1014	recessive model	1.43 (0.66 , 3.12)	0.369	35.69%	0.815	0.773	0.507	BBB	0.962	0.999	1.000	1.000	
				10	1238/1014	homozygous model	1.45 (0.66 , 3.19)	0.351	35.89%	0.886	0.778	0.418	CBB	0.962	0.999	1.000	1.000	
ICAM1 rs5498	G/A	Microvascular complications	Asian	4	757/606	dominant model	0.57 (0.37 , 0.87)	0.009	58.15%	0.011	0.306	0.916	ACC	0.935	0.999	1.000	1.000	Credible
				4	757/606	recessive model	0.72 (0.45 , 1.15)	0.170	58.47%	0.135	0.800	0.300	CCB	0.948	0.999	1.000	1.000	
				4	757/606	additive model	1.28 (0.96 , 1.7)	0.092	29.12%	0.188	1.000	0.428	CBB	0.929	0.999	1.000	1.000	
MMP-9 rs3918242	T/C	Microvascular complications	European/Asian	4	447/700	dominant model	0.69 (0.39 , 1.22)	0.203	75.13%	0.002	0.889	0.364	CCC	0.949	0.999	1.000	1.000	Not credible
TLR4 rs4986790	G/A	Microvascular complications	European/Asian	11	1834/4069	allelic model	1.5 (1.11 , 2.03)	0.009	56.44%	0.876	0.470	0.984	ACB	0.723	0.993	0.999	1.000	Credible
				11	1834/4069	dominant model	1.52 (1.1 , 2.09)	0.012	57.94%	0.862	0.466	0.982	ACB	0.745	0.994	0.999	1.000	
				5	1492/3020	recessive model	1.82 (0.74 , 4.43)	0.190	0.00%	0.622	1.000	0.241	CAB	0.951	0.999	1.000	1.000	
				5	1492/3020	homozygous model	1.88 (0.77 , 4.58)	0.167	0.00%	0.640	1.000	0.267	CAB	0.948	0.999	1.000	1.000	
			European	9	1695/3730	allelic model	1.66 (1.22 , 2.26)	0.001	52.15%	0.473	0.464	0.997	ACB	0.366	0.968	0.997	1.000	Credible
				9	1695/3730	dominant model	1.69 (1.22 , 2.35)	0.002	53.75%	0.435	0.462	0.997	ACB	0.433	0.976	0.998	1.000	
AGER rs1800624	A/T	CVD	European/Asian/ African	7	1058/1298	allelic model	0.94 (0.75 , 1.17)	0.561	55.20%	0.634	0.424	0.105	CCB	0.957	0.999	1.000	1.000	Not credible
				6	881/1129	dominant model	0.91 (0.69 , 1.22)	0.534	46.18%	0.650	0.675	0.094	CBB	0.956	0.999	1.000	1.000	
AGER rs1800625	C/T	CVD	European/Asian/ African	6	1004/1244	allelic model	1.11 (0.91 , 1.36)	0.296	18.54%	0.735	1.000	0.213	CAB	0.960	0.999	1.000	1.000	Not credible
				5	827/1075	dominant model	1.18 (0.88 , 1.59)	0.278	40.44%	0.434	0.639	0.302	CBB	0.959	0.999	1.000	1.000	
AGER rs2070600	A/G	CVD	European/Asian	3	584/780	allelic model	1 (0.83 , 1.22)	0.980	3.82%	0.805	1.000	0.024	CAB	0.964	0.999	1.000	1.000	Not credible
				4	617/898	dominant model	0.94 (0.73 , 1.2)	0.606	11.26%	0.555	1.000	0.083	CAB	0.958	0.999	1.000	1.000	
eNOS rs1799983	T/G	CAD	European/Asian	6	637/1881	allelic model	1.24 (0.88 , 1.74)	0.222	73.60%	0.492	0.938	0.131	CCB	0.954	0.999	1.000	1.000	Not credible
				6	637/1881	dominant model	1.21 (0.84 , 1.75)	0.309	65.51%	0.535	0.876	0.120	CCB	0.960	0.999	1.000	1.000	
				5	609/1831	recessive model	1.38 (0.73 , 2.6)	0.326	55.26%	0.178	0.776	0.070	CCB	0.961	0.999	1.000	1.000	
				6	637/1881	codominant model	1.12 (0.84 , 1.49)	0.452	43.38%	0.444	0.669	0.068	CBB	0.964	0.999	1.000	1.000	
			Asian	3	242/454	allelic model	1.75 (0.87 , 3.52)	0.118	66.32%	0.558	0.841	0.804	ACB	0.939	0.999	1.000	1.000	Not credible
				3	242/454	dominant model	1.8 (0.91 , 3.55)	0.092	56.57%	0.538	0.806	0.775	BCB	0.929	0.999	1.000	1.000	
				3	242/454	codominant model	1.67 (0.94 , 2.95)	0.081	38.35%	0.586	0.716	0.632	BBB	0.923	0.998	1.000	1.000	
GSTM1	NA	CAD	European/Asian	6	789/1183	Gene (GSTM1), null vs present	1.07 (0.87 , 1.31)	0.534	7.41%	0.290	1.000	0.084	CAB	0.965	0.999	1.000	1.000	Not credible
GSTT1	NA	CAD	European/Asian	6	789/1183	Gene (GSTT1), null vs present	1.09 (0.73 , 1.61)	0.686	63.04%	0.503	0.815	0.205	CCB	0.966	0.999	1.000	1.000	Not credible
				7	864/1280	allelic model	1.16 (0.89 , 1.5)	0.275	72.32%	0.015	0.733	0.128	CCC	0.957	0.999	1.000	1.000	
				7	864/1280	dominant model	1.12 (0.81 , 1.55)	0.506	44.94%	0.157	0.717	0.092	CBB	0.965	0.999	1.000	1.000	

SERPINE1 rs1799889	4G/5G	CVD	European/Asian	7	864/1280	recessive model	1.2 (0.84 , 1.7)	0.322	66.41%	0.014	0.907	0.095	CCC	0.960	0.999	1.000	1.000	Not credible
				7	864/1280	heterozygous model	1.05 (0.83 , 1.33)	0.703	0.00%	0.528	1.000	0.053	CAB	0.966	0.999	1.000	1.000	
				7	864/1280	homozygous model	1.23 (0.77 , 1.96)	0.379	64.24%	0.037	0.887	0.102	CCC	0.963	0.999	1.000	1.000	
			European	4	610/1042	allelic model	1.09 (0.77 , 1.54)	0.638	80.75%	0.168	0.872	0.067	CCB	0.966	0.999	1.000	1.000	Not credible
				4	610/1042	dominant model	1.02 (0.68 , 1.53)	0.920	53.82%	0.421	1.000	0.030	CCB	0.965	0.999	1.000	1.000	
				4	610/1042	recessive model	1.15 (0.7 , 1.89)	0.569	78.62%	0.152	0.857	0.083	CCB	0.966	0.999	1.000	1.000	
				4	610/1042	heterozygous model	1 (0.73 , 1.36)	0.991	17.82%	0.821	1.000	0.022	CAB	0.964	0.999	1.000	1.000	
				4	610/1042	homozygous model	1.11 (0.59 , 2.09)	0.740	75.03%	0.218	1.000	0.045	CCB	0.966	0.999	1.000	1.000	
			Asian	3	254/238	allelic model	1.36 (0.74 , 2.48)	0.321	77.04%	0.110	0.824	0.121	CCB	0.960	0.999	1.000	1.000	Not credible
				3	254/238	dominant model	1.4 (0.66 , 2.96)	0.378	57.70%	0.603	0.609	0.171	CCB	0.963	0.999	1.000	1.000	
				3	254/238	recessive model	1.42 (0.62 , 3.23)	0.404	71.19%	0.003	1.000	0.041	CCC	0.963	0.999	1.000	1.000	
				3	254/238	heterozygous model	1.25 (0.63 , 2.46)	0.521	40.28%	0.985	1.000	0.099	CBB	0.965	0.999	1.000	1.000	
				3	254/238	homozygous model	1.62 (0.57 , 4.63)	0.368	71.02%	0.131	0.745	0.120	CCB	0.962	0.999	1.000	1.000	
GSTM1	NA	Cerebrovascular disease	European/Asian	5	846/519	Gene (GSTM1), null vs present	1.22 (0.88 , 1.69)	0.239	22.07%	0.114	0.610	0.660	BAB	0.956	0.999	1.000	1.000	Not credible
GSTT1	NA	Cerebrovascular disease	European/Asian	5	846/519	Gene (GSTT1), null vs present	1.52 (1.03 , 2.25)	0.035	19.88%	0.238	0.334	0.924	AAB	0.881	0.997	1.000	1.000	Credible
NADPH rs4673	T/C	Carotid atherosclerosis	European/Asian	3	486/273	allelic model	0.85 (0.38 , 1.92)	0.703	86.06%	0.057	0.864	0.158	CCC	0.959	0.999	1.000	1.000	Not credible
				3	486/273	dominant model	0.89 (0.28 , 2.83)	0.847	85.22%	0.001	0.431	0.061	CCC	0.962	0.999	1.000	1.000	
				3	486/273	recessive model	1.03 (0.4 , 2.64)	0.957	60.59%	0.756	0.586	0.188	CCB	0.965	0.999	1.000	1.000	
				3	486/273	homozygous model	1.12 (0.26 , 4.84)	0.877	76.19%	0.692	0.739	0.356	CCB	0.965	0.999	1.000	1.000	
eNOS rs1799983	T/G	Peripheral vascular disease	European	4	344/685	allelic model	0.74 (0.57 , 0.97)	0.026	32.09%	0.621	0.789	0.773	BBB	0.949	0.999	1.000	1.000	Credible
				4	344/685	dominant model	0.79 (0.56 , 1.12)	0.191	33.73%	0.468	0.575	0.318	CBB	0.948	0.999	1.000	1.000	
				4	344/685	recessive model	0.39 (0.21 , 0.7)	0.002	11.95%	0.932	0.875	0.947	AAB	0.852	0.997	1.000	1.000	
				4	344/685	codominant model	1.08 (0.8 , 1.44)	0.628	10.87%	0.577	1.000	0.067	CAB	0.966	0.999	1.000	1.000	
AGER rs1800624	A/T	All vsacular complications	European/Asian/ African	15	3756/4727	allelic model	0.93 (0.84 , 1.02)	0.101	26.08%	0.663	0.578	0.662	BBB	0.949	0.999	1.000	1.000	Credible
				11	2801/4093	dominant model	0.93 (0.8 , 1.08)	0.334	39.31%	0.576	0.495	0.401	CBB	0.951	0.999	1.000	1.000	
				10	2695/3984	recessive model	0.71 (0.58 , 0.88)	0.001	0.00%	0.412	0.866	0.897	AAB	0.860	0.997	1.000	1.000	
			European	9	2727/3846	allelic model	0.91 (0.84 , 0.99)	0.027	0.00%	0.240	0.674	0.601	BAB	0.949	0.999	1.000	1.000	Credible
				7	2086/3427	dominant model	0.92 (0.82 , 1.04)	0.179	5.27%	0.360	0.496	0.324	CAB	0.948	0.999	1.000	1.000	
				6	1980/3318	recessive model	0.67 (0.54 , 0.84)	0.001	0.00%	0.811	0.890	0.940	AAB	0.717	0.993	0.999	1.000	
GSTM1	NA	All vsacular complications	European/Asian	21	2639/2790	Gene (GSTM1), null vs present	0.95 (0.74 , 1.23)	0.714	73.80%	0.772	0.637	0.112	CCB	0.959	0.999	1.000	1.000	Not credible
			Asian	7	798/699	Gene (GSTM1), null vs present	1.39 (1.05 , 1.83)	0.022	37.12%	0.181	0.882	0.739	BBB	0.824	0.996	1.000	1.000	Credible
			European	14	1841/2091	Gene (GSTM1), null vs present	0.75 (0.54 , 1.05)	0.093	76.11%	0.691	0.721	0.747	BCB	0.949	0.999	1.000	1.000	Not credible
GSTT1	NA	All vsacular complications	European/Asian	19	2450/2598	Gene (GSTT1), null vs present	1.43 (1.13 , 1.81)	0.003	60.33%	0.384	0.495	1.000	ACB	0.530	0.983	0.998	1.000	Credible
			Asian	5	609/507	Gene (GSTT1), null vs present	1.74 (1.16 , 2.61)	0.008	58.54%	0.901	0.438	0.997	ACB	0.700	0.992	0.999	1.000	Credible
			European	14	1841/2091	Gene (GSTT1), null vs present	1.3 (0.98 , 1.73)	0.067	58.51%	0.340	0.706	0.964	ACB	0.920	0.998	1.000	1.000	Not credible
GSTM1/GSTT1	NA	All vsacular complications	European/Asian	4	259/281	Gene (GSTM1/GSTT1), dual null vs present	1.94 (1.03 , 3.64)	0.040	42.51%	0.786	0.410	0.843	ABB	0.886	0.998	1.000	1.000	Credible

A1/A2, alternative allele and reference allele for the genetic variants; allelic model, A1 vs. A2, dominant model, A1A1+A1A2 vs. A2A2, recessive model, A1A1 vs. A1A2+A2A2, heterozygous model, A1A2 vs A2A2, homozygous model, A1A1 vs A2A2, codominant model, A1A2 vs. A1A1+A2A2, additive model, A1A1+A2A2 vs. A1A2; P.effect, the P-value for meta-analyses effects; P.Egger, the P-value for small study effects; P.Sig, the P-value for excess significance; BFDP1, BFDP2, BFDP3, and BFDP4 were respectively calculated at the prior level of 0.05, 10-3, 10-4, and 10-6. DR, diabetic retinopathy; PDR, proliferative diabetic retinopathy; NPDR, non-proliferative diabetic retinopathy; DKD, diabetic kidney disease; ESRD, end-stage renal disease; DPN, diabetic peripheral neuropathy; DF, diabetic foot; CVD, cardiovascular disease; CAD, coronary artery disease.

Supplementary Table 5. Summary of "credible" and "not credible" associations in mixed types of diabetes.

Factor	A1/A2	Outcome	Population	No. study	Sample size (cases/controls)	Genetic model	OR (95% CIs)	P.effct	I2	P.Egger	P.Sig	Power	Venice criteria	BFDP1	BFDP2	BFDP3	BFDP4	Credibility
ACE Ins/Del	Del/Ins	DR	European/Asian	13	1665/1556	allelic model	1.04 (0.94 , 1.16)	0.426	0.00%	0.532	0.525	0.117	CAB	0.965	0.999	1.000	1.000	Not credible
			European	10	997/920	allelic model	1.05 (0.92 , 1.2)	0.468	4.90%	0.998	0.477	0.104	CAB	0.965	0.999	1.000	1.000	Not credible
			European/Asian	7	672/901	allelic model	1.15 (0.92 , 1.43)	0.215	40.20%	0.227	0.349	0.431	CBB	0.954	0.999	1.000	1.000	Not credible
			European	4	372/265	allelic model	1.06 (0.71 , 1.57)	0.789	62.80%	0.231	0.667	0.133	CCB	0.966	0.999	1.000	1.000	Not credible
ALR (AC)n	NA	DR	European/Asian/African	15	1236/1351	allele (Z), present vs null	0.93 (0.81 , 1.05)	0.235	0.00%	0.499	0.611	0.209	CAB	0.949	0.999	1.000	1.000	Credible
				16	1400/1509	allele (Z+2), present vs null	0.63 (0.47 , 0.85)	0.003	75.31%	0.061	0.861	0.985	ACC	0.884	0.998	1.000	1.000	
				16	1400/1509	allele (Z-2), present vs null	1.6 (1.27 , 2.01)	7.33E-05	55.90%	0.118	0.350	1.000	ACB	0.037	0.670	0.953	1.000	
				13	1132/1293	allele (Z+4), present vs null	0.75 (0.59 , 0.96)	0.020	0.00%	0.375	1.000	0.587	BAB	0.947	0.999	1.000	1.000	
				14	1189/1323	allele (Z-4), present vs null	1.3 (0.96 , 1.76)	0.087	27.56%	0.836	0.873	0.593	BBB	0.929	0.999	1.000	1.000	
				12	1043/1255	allele (Z+6), present vs null	0.81 (0.52 , 1.27)	0.366	16.77%	0.581	1.000	0.212	CAB	0.951	0.999	1.000	1.000	
				12	980/750	allele (Z-6), present vs null	0.77 (0.45 , 1.31)	0.333	0.00%	0.229	1.000	0.091	CAB	0.951	0.999	1.000	1.000	
			Asian	10	759/1064	allele (Z), present vs null	0.93 (0.79 , 1.08)	0.333	0.00%	0.980	1.000	0.150	CAB	0.951	0.999	1.000	1.000	Credible
				11	923/1222	allele (Z+2), present vs null	0.67 (0.47 , 0.96)	0.030	76.97%	0.016	0.704	0.643	BCC	0.949	0.999	1.000	1.000	
				11	923/1222	allele (Z-2), present vs null	1.6 (1.19 , 2.16)	0.002	58.67%	0.049	0.331	0.994	ACC	0.467	0.979	0.998	1.000	
				9	702/1034	allele (Z+4), present vs null	0.79 (0.61 , 1.03)	0.084	0.00%	0.277	1.000	0.353	CAB	0.949	0.999	1.000	1.000	
				10	759/1064	allele (Z-4), present vs null	1.23 (0.82 , 1.84)	0.311	45.39%	0.988	0.853	0.347	CBB	0.960	0.999	1.000	1.000	
				8	613/996	allele (Z+6), present vs null	0.9 (0.5 , 1.63)	0.727	39.96%	0.304	1.000	0.145	CBB	0.960	0.999	1.000	1.000	
				8	550/491	allele (Z-6), present vs null	0.58 (0.31 , 1.08)	0.083	0.00%	0.304	1.000	0.364	CAB	0.949	0.999	1.000	1.000	
			European	4	415/221	allele (Z), present vs null	0.89 (0.65 , 1.21)	0.463	38.52%	0.282	0.486	0.183	CBB	0.954	0.999	1.000	1.000	Credible
				4	415/221	allele (Z+2), present vs null	0.53 (0.33 , 0.85)	0.009	51.35%	0.185	0.934	0.990	ACB	0.933	0.999	1.000	1.000	
				4	415/221	allele (Z-2), present vs null	1.8 (1.18 , 2.74)	0.007	54.01%	0.576	0.711	0.997	ACB	0.668	0.991	0.999	1.000	
				3	368/193	allele (Z+4), present vs null	0.65 (0.28 , 1.53)	0.324	45.37%	0.239	1.000	0.314	CBB	0.951	0.999	1.000	1.000	
				3	368/193	allele (Z-4), present vs null	1.57 (0.87 , 2.81)	0.133	8.15%	0.765	1.000	0.317	CAB	0.942	0.999	1.000	1.000	
				3	368/193	allele (Z+6), present vs null	0.62 (0.15 , 2.52)	0.501	18.08%	0.748	1.000	0.078	CAB	0.955	0.999	1.000	1.000	
				3	368/193	allele (Z-6), present vs null	1.76 (0.56 , 5.56)	0.335	5.72%	0.867	1.000	0.150	CAB	0.961	0.999	1.000	1.000	
AKR1B1 rs759853	T/C	DR	European/Asian/Middle Eastern/African	22	4244/4900	allelic model	0.9 (0.76 , 1.05)	0.189	73.27%	0.513	0.185	0.732	BCB	0.948	0.999	1.000	1.000	Not credible
				22	4244/4900	dominant model	0.87 (0.71 , 1.06)	0.169	73.38%	0.260	0.010	0.830	ACB	0.948	0.999	1.000	1.000	
				22	4244/4900	heterozygous model	0.87 (0.7 , 1.07)	0.181	70.26%	0.343	0.050	0.819	ACB	0.948	0.999	1.000	1.000	
			East Asian	13	2923/3933	allelic model	1 (0.81 , 1.23)	0.975	74.94%	0.116	0.118	0.637	BCB	0.964	0.999	1.000	1.000	Not credible
				13	2923/3933	dominant model	0.96 (0.73 , 1.26)	0.785	77.94%	0.334	0.056	0.664	BCB	0.961	0.999	1.000	1.000	
				13	2923/3933	heterozygous model	0.94 (0.71 , 1.24)	0.655	76.39%	0.528	0.045	0.603	BCB	0.959	0.999	1.000	1.000	
			European	3	491/283	allelic model	0.86 (0.52 , 1.44)	0.571	79.59%	0.229	0.795	0.077	CCB	0.957	0.999	1.000	1.000	Not credible
				3	491/283	dominant model	0.92 (0.42 , 2.01)	0.831	81.20%	0.200	0.794	0.034	CCB	0.962	0.999	1.000	1.000	
				3	491/283	heterozygous model	1.03 (0.48 , 2.21)	0.934	76.94%	0.258	1.000	0.100	CCB	0.965	0.999	1.000	1.000	
			South Asian/African	3	616/527	allelic model	0.92 (0.55 , 1.54)	0.742	73.21%	0.200	1.000	0.136	CCB	0.960	0.999	1.000	1.000	Not credible
				3	616/527	dominant model	0.87 (0.49 , 1.55)	0.640	62.62%	0.359	1.000	0.045	CCB	0.958	0.999	1.000	1.000	
				3	616/527	heterozygous model	0.83 (0.52 , 1.33)	0.449	40.92%	0.700	1.000	0.249	CBB	0.953	0.999	1.000	1.000	
				4	346/377	allelic model	1.16 (0.69 , 1.93)	0.578	75.52%	0.110	0.828	0.548	BCB	0.966	0.999	1.000	1.000	

		NPDR	European/Asian/Middle Eastern/African	4	346/377	dominant model	1.33 (0.65 , 2.73)	0.435	77.98%	0.019	0.866	0.868	ACC	0.964	0.999	1.000	1.000	Not credible
				4	346/377	heterozygous model	1.47 (0.72 , 2.98)	0.290	74.42%	0.023	0.489	0.923	ACC	0.959	0.999	1.000	1.000	
eNOS rs2070744	C/T	DR	European/Asian	7	2045/1738	allelic model	1.06 (0.95 , 1.18)	0.275	5.18%	0.255	0.442	0.210	CAB	0.959	0.999	1.000	1.000	Not credible
				7	2045/1738	dominant model	1.09 (0.95 , 1.25)	0.201	0.00%	0.531	0.433	0.238	CAB	0.954	0.999	1.000	1.000	
				7	2045/1738	recessive model	1.05 (0.84 , 1.31)	0.687	6.41%	0.289	1.000	0.058	CAB	0.966	0.999	1.000	1.000	
				7	2045/1738	codominant model	1.07 (0.94 , 1.23)	0.309	0.00%	0.992	1.000	0.164	CAB	0.961	0.999	1.000	1.000	
			European	6	1928/1629	allelic model	1.07 (0.97 , 1.19)	0.168	0.00%	0.583	0.424	0.271	CAB	0.954	0.999	1.000	1.000	Not credible
				6	1928/1629	dominant model	1.11 (0.97 , 1.27)	0.141	0.00%	0.874	0.445	0.302	CAB	0.942	0.999	1.000	1.000	
				6	1928/1629	recessive model	1.06 (0.85 , 1.32)	0.605	5.12%	0.628	1.000	0.073	CAB	0.966	0.999	1.000	1.000	
				6	1928/1629	codominant model	1.08 (0.94 , 1.24)	0.259	0.00%	0.722	1.000	0.193	CAB	0.958	0.999	1.000	1.000	
EPO rs1617640	T/G	DR	European/Asian	5	1230/1170	allelic model	0.96 (0.77 , 1.2)	0.717	58.08%	0.185	0.703	0.031	CCB	0.960	0.999	1.000	1.000	Not credible
				5	1230/1170	dominant model	1.06 (0.7 , 1.59)	0.795	36.46%	0.102	0.565	0.027	CBB	0.966	0.999	1.000	1.000	
				5	1230/1170	recessive model	0.99 (0.79 , 1.23)	0.905	22.09%	0.755	0.367	0.028	CAB	0.963	0.999	1.000	1.000	
				5	1230/1170	heterozygous model	1.03 (0.68 , 1.55)	0.895	31.74%	0.132	0.519	0.032	CBB	0.965	0.999	1.000	1.000	
				5	1230/1170	homozygous model	1.11 (0.68 , 1.79)	0.679	36.14%	0.041	0.507	0.021	CBC	0.966	0.999	1.000	1.000	
EPO rs551238	A/C	DR	European/Asian	3	627/989	allelic model	0.77 (0.55 , 1.07)	0.119	62.51%	0.962	0.367	0.858	ACB	0.949	0.999	1.000	1.000	Not credible
				3	627/989	dominant model	0.51 (0.19 , 1.34)	0.173	74.26%	0.315	0.434	0.831	ACB	0.948	0.999	1.000	1.000	
				3	627/989	recessive model	0.79 (0.58 , 1.09)	0.154	38.20%	0.788	1.000	0.584	BBB	0.948	0.999	1.000	1.000	
				3	627/989	heterozygous model	0.56 (0.24 , 1.29)	0.175	62.82%	0.344	0.317	0.686	BCB	0.948	0.999	1.000	1.000	
				3	627/989	homozygous model	0.49 (0.17 , 1.39)	0.179	75.53%	0.389	0.449	0.871	ACB	0.948	0.999	1.000	1.000	
SOD2 rs4880	C/T	DR	European/Asian	5	696/644	allelic model	0.76 (0.5 , 1.15)	0.191	79.53%	0.953	0.391	0.899	ACB	0.948	0.999	1.000	1.000	Not credible
				5	696/644	dominant model	0.68 (0.44 , 1.06)	0.087	60.45%	0.577	0.220	0.790	BCB	0.949	0.999	1.000	1.000	
				5	696/644	recessive model	0.77 (0.41 , 1.46)	0.428	72.17%	0.647	0.177	0.588	BCB	0.953	0.999	1.000	1.000	
TNF-α rs361525	A/G	DR	European/Asian	3	659/576	allelic model	1.55 (0.89 , 2.71)	0.120	63.62%	0.855	0.844	0.994	ACB	0.941	0.999	1.000	1.000	Not credible
				3	659/576	dominant model	1.59 (0.93 , 2.71)	0.090	54.08%	0.873	0.814	0.950	ACB	0.929	0.999	1.000	1.000	
				3	659/576	heterozygous model	1.5 (0.99 , 2.27)	0.054	28.39%	0.883	0.722	0.777	BBB	0.907	0.998	1.000	1.000	
TNF-α rs1800629	A/G	DR	European/Asian	9	1698/2064	allelic model	1.09 (0.95 , 1.24)	0.211	0.00%	0.955	0.482	0.233	CAB	0.952	0.999	1.000	1.000	Not credible
				9	1698/2064	dominant model	1.13 (0.97 , 1.31)	0.126	0.00%	0.792	0.533	0.322	CAB	0.935	0.999	1.000	1.000	
				8	1623/1888	recessive model	0.95 (0.64 , 1.41)	0.790	0.00%	0.620	1.000	0.025	CAB	0.961	0.999	1.000	1.000	
				9	1698/2064	heterozygous model	1.14 (0.97 , 1.33)	0.113	0.00%	0.537	1.000	0.342	CAB	0.932	0.999	1.000	1.000	
				8	1623/1888	homozygous model	0.99 (0.66 , 1.49)	0.979	0.00%	0.670	1.000	0.023	CAB	0.964	0.999	1.000	1.000	
			European	5	1099/1276	allelic model	1.12 (0.9 , 1.4)	0.315	42.14%	0.686	0.657	0.225	CBB	0.961	0.999	1.000	1.000	Not credible
				5	1099/1276	dominant model	1.18 (0.93 , 1.5)	0.183	34.22%	0.369	0.659	0.336	CBB	0.950	0.999	1.000	1.000	
				5	1099/1276	recessive model	0.94 (0.52 , 1.7)	0.825	26.09%	0.565	1.000	0.031	CBB	0.962	0.999	1.000	1.000	
				5	1099/1276	heterozygous model	1.19 (0.94 , 1.51)	0.145	28.41%	0.155	1.000	0.366	CBB	0.946	0.999	1.000	1.000	
				5	1099/1276	homozygous model	1 (0.54 , 1.83)	0.991	26.99%	0.645	1.000	0.017	CBB	0.964	0.999	1.000	1.000	
			European/Asian	15	2701/2097	allelic model	1.09 (0.95 , 1.26)	0.229	63.14%	0.068	0.323	0.278	CCC	0.956	0.999	1.000	1.000	Not credible
				15	2701/2097	dominant model	1.12 (0.92 , 1.36)	0.268	56.59%	0.066	0.398	0.187	CCC	0.957	0.999	1.000	1.000	
				15	2701/2097	recessive model	1.19 (0.89 , 1.58)	0.238	61.87%	0.004	0.124	0.223	CCC	0.955	0.999	1.000	1.000	
				15	2701/2097	heterozygous model	1.07 (0.89 , 1.29)	0.457	46.02%	0.057	0.924	0.086	CBC	0.965	0.999	1.000	1.000	

VEGF rs2010963	C/G	DR		15	2701/2097	homozygous model	1.34 (0.97 , 1.85)	0.080	60.28%	0.010	0.326	0.481	CCC	0.922	0.998	1.000	1.000	
			European	5	1009/695	allelic model	0.93 (0.74 , 1.18)	0.543	58.97%	0.062	0.727	0.313	CCC	0.956	0.999	1.000	1.000	Not credible
				5	1009/695	dominant model	0.82 (0.64 , 1.06)	0.127	27.91%	0.445	0.629	0.493	CBB	0.949	0.999	1.000	1.000	
				5	1009/695	recessive model	1.14 (0.62 , 2.11)	0.674	71.74%	0.003	0.477	0.035	CCC	0.966	0.999	1.000	1.000	
				5	1009/695	heterozygous model	0.82 (0.63 , 1.07)	0.146	27.00%	0.789	0.604	0.422	CBB	0.948	0.999	1.000	1.000	
				5	1009/695	homozygous model	0.97 (0.55 , 1.74)	0.930	60.35%	0.005	0.717	0.190	CCC	0.963	0.999	1.000	1.000	
VEGF rs2146323	C/A	DR	European/Asian	4	512/571	allelic model	0.89 (0.62 , 1.28)	0.516	71.49%	0.205	0.795	0.439	CCB	0.956	0.999	1.000	1.000	Credible
				4	512/571	dominant model	1.31 (0.29 , 5.92)	0.724	90.60%	0.068	0.954	0.056	CCC	0.966	0.999	1.000	1.000	
				4	512/571	recessive model	0.71 (0.54 , 0.93)	0.015	15.76%	0.311	0.755	0.789	BAB	0.941	0.999	1.000	1.000	
				4	512/571	heterozygous model	1.59 (0.35 , 7.33)	0.551	89.83%	0.074	0.953	0.273	CCC	0.965	0.999	1.000	1.000	
				4	512/571	homozygous model	1.08 (0.25 , 4.63)	0.920	88.83%	0.067	0.937	0.064	CCC	0.965	0.999	1.000	1.000	
			European	3	383/433	allelic model	0.99 (0.67 , 1.46)	0.949	65.78%	0.086	1.000	0.130	CCC	0.964	0.999	1.000	1.000	Not credible
				3	383/433	dominant model	2 (0.31 , 12.69)	0.463	90.74%	0.050	0.924	0.248	CCC	0.964	0.999	1.000	1.000	
				3	383/433	recessive model	0.74 (0.54 , 1.02)	0.068	14.53%	0.281	0.567	0.554	BAB	0.950	0.999	1.000	1.000	
				3	383/433	heterozygous model	2.41 (0.36 , 16.21)	0.366	90.45%	0.058	0.930	0.539	BCC	0.962	0.999	1.000	1.000	
				3	383/433	homozygous model	1.61 (0.27 , 9.76)	0.603	88.85%	0.050	0.896	0.041	CCC	0.966	0.999	1.000	1.000	
VEGF rs3025039	T/C	DR	European/Asian	5	702/636	allelic model	1.48 (1.09 , 2.01)	0.012	54.04%	0.004	0.356	0.994	ACC	0.771	0.994	0.999	1.000	Credible
				5	702/636	dominant model	1.6 (1.04 , 2.48)	0.033	68.54%	0.001	0.758	0.996	ACC	0.879	0.997	1.000	1.000	
				4	572/554	recessive model	1.47 (0.63 , 3.41)	0.376	43.31%	0.831	1.000	0.313	CBB	0.962	0.999	1.000	1.000	
				5	702/636	heterozygous model	1.58 (0.99 , 2.51)	0.055	70.37%	0.001	0.733	0.991	ACC	0.904	0.998	1.000	1.000	
				4	572/554	homozygous model	1.93 (0.85 , 4.39)	0.117	38.68%	0.645	0.688	0.698	BBB	0.939	0.999	1.000	1.000	
VEGF rs699947	A/C	DR	European/Asian	9	1205/1277	allelic model	1.23 (1.02 , 1.49)	0.032	54.01%	0.595	0.503	0.856	ACB	0.877	0.997	1.000	1.000	Credible
				9	1205/1277	dominant model	1.42 (1.07 , 1.89)	0.016	58.66%	0.062	0.979	0.949	ACC	0.807	0.995	1.000	1.000	
				9	1205/1277	recessive model	1.11 (0.76 , 1.63)	0.582	44.17%	0.188	0.784	0.119	CBB	0.966	0.999	1.000	1.000	
				9	1205/1277	heterozygous model	1.41 (1.05 , 1.9)	0.022	57.51%	0.030	0.973	0.917	ACC	0.847	0.997	1.000	1.000	
				9	1205/1277	homozygous model	1.4 (0.92 , 2.14)	0.113	43.03%	0.924	0.563	0.562	BBB	0.939	0.999	1.000	1.000	
			European	5	471/510	allelic model	1.15 (0.96 , 1.38)	0.132	0.00%	0.481	1.000	0.309	CAB	0.943	0.999	1.000	1.000	Credible
				5	471/510	dominant model	1.44 (1.08 , 1.93)	0.013	0.00%	0.053	1.000	0.748	BAC	0.796	0.995	1.000	1.000	
				5	471/510	recessive model	0.97 (0.71 , 1.33)	0.857	0.00%	0.086	1.000	0.047	CAC	0.962	0.999	1.000	1.000	
				5	471/510	heterozygous model	1.56 (1.1 , 2.19)	0.011	12.72%	0.035	1.000	0.823	AAC	0.748	0.994	0.999	1.000	
				5	471/510	homozygous model	1.25 (0.84 , 1.84)	0.268	0.00%	0.872	1.000	0.144	CAB	0.957	0.999	1.000	1.000	
VEGF rs833061	T/C	DR	European/Asian	6	965/871	allelic model	0.73 (0.5 , 1.07)	0.107	82.82%	0.267	0.351	0.946	ACB	0.949	0.999	1.000	1.000	Not credible
				6	965/871	dominant model	0.69 (0.36 , 1.32)	0.260	68.13%	0.146	0.608	0.343	CCB	0.949	0.999	1.000	1.000	
				6	965/871	recessive model	0.59 (0.33 , 1.06)	0.078	84.38%	0.647	0.686	0.964	ACB	0.950	0.999	1.000	1.000	
				6	965/871	heterozygous model	0.78 (0.44 , 1.39)	0.401	56.90%	0.237	0.408	0.131	CCB	0.952	0.999	1.000	1.000	
				6	965/871	homozygous model	0.49 (0.21 , 1.17)	0.109	71.47%	0.235	0.742	0.800	ACB	0.949	0.999	1.000	1.000	
		PDR	European/Asian	4	512/643	allelic model	0.81 (0.61 , 1.08)	0.145	50.98%	0.423	0.734	0.652	BCB	0.948	0.999	1.000	1.000	Not credible
				4	512/643	dominant model	0.78 (0.42 , 1.46)	0.441	62.04%	0.395	0.706	0.205	CCB	0.953	0.999	1.000	1.000	
				4	512/643	recessive model	0.69 (0.42 , 1.14)	0.148	57.35%	0.534	0.382	0.656	BCB	0.948	0.999	1.000	1.000	
				4	512/643	heterozygous model	0.88 (0.49 , 1.6)	0.678	54.59%	0.343	0.589	0.056	CCB	0.959	0.999	1.000	1.000	

				4	512/643	homozygous model	1.56 (0.27 , 1.16)	0.118	49.20%	0.783	0.751	0.634	BBB	0.949	0.999	1.000	1.000	
ADIPOQ rs17300539	A/G	DKD	European	7	2749/7585	allelic model	1.24 (1.06 , 1.45)	0.008	30.72%	0.089	0.629	0.809	ABC	0.691	0.992	0.999	1.000	Credible
ADIPOQ rs266729	G/C	DKD	European/Asian	8	3127/3911	allelic model	0.97 (0.89 , 1.05)	0.421	7.25%	0.276	0.443	0.117	CAB	0.954	0.999	1.000	1.000	Not credible
			European	5	2605/3405	allelic model	0.98 (0.91 , 1.07)	0.698	0.00%	0.339	1.000	0.056	CAB	0.958	0.999	1.000	1.000	Not credible
ADIPOQ rs2241766	G/T	DKD	European/Asian	9	2654/7710	allelic model	1.1 (1 , 1.22)	0.048	0.00%	0.171	0.627	0.470	CAB	0.920	0.998	1.000	1.000	Credible
			European	7	2367/7222	allelic model	1.13 (1.01 , 1.26)	0.031	0.00%	0.339	0.638	0.538	BAB	0.860	0.997	1.000	1.000	Credible
ADIPOQ rs1501299	T/G	DKD	European/Asian	10	2812/7821	allelic model	1.05 (0.98 , 1.13)	0.188	0.89%	0.567	1.000	0.254	CAB	0.952	0.999	1.000	1.000	Not credible
			European	6	2339/7017	allelic model	1.08 (0.97 , 1.2)	0.170	31.49%	0.366	1.000	0.338	CBB	0.946	0.999	1.000	1.000	Not credible
ACE Ins/Del	Del/Ins	DKD	European	20	6061/3358	allelic model	1.07 (0.99 , 1.15)	0.091	11.76%	0.990	0.426	0.481	CAB	0.916	0.998	1.000	1.000	Credible
				20	6061/3358	dominant model	1.18 (1.01 , 1.37)	0.032	26.83%	0.559	0.198	0.777	BBB	0.866	0.997	1.000	1.000	
				20	6061/3358	recessive model	1.03 (0.93 , 1.13)	0.590	0.00%	0.653	0.513	0.077	CAB	0.965	0.999	1.000	1.000	
				20	6061/3358	codominant model	1.08 (0.98 , 1.18)	0.106	0.00%	0.182	0.881	0.363	CAB	0.929	0.999	1.000	1.000	
AGER rs1800624	A/T	DKD	European/Asian/African	7	1404/1540	allelic model	1.03 (0.85 , 1.25)	0.746	58.39%	0.649	0.809	0.057	CCB	0.966	0.999	1.000	1.000	Not credible
				6	1146/1345	dominant model	1.06 (0.79 , 1.43)	0.702	65.36%	0.896	0.830	0.066	CCB	0.966	0.999	1.000	1.000	
				7	1471/1666	recessive model	0.84 (0.65 , 1.09)	0.181	0.00%	0.714	1.000	0.263	CAB	0.948	0.999	1.000	1.000	
			European	5	1081/1348	allelic model	1.08 (0.86 , 1.35)	0.523	67.84%	0.053	0.815	0.100	CCC	0.965	0.999	1.000	1.000	Not credible
				4	823/1153	dominant model	1.14 (0.74 , 1.74)	0.550	79.66%	0.303	0.870	0.110	CCB	0.965	0.999	1.000	1.000	
				5	1148/1474	recessive model	0.86 (0.65 , 1.12)	0.261	2.22%	0.352	1.000	0.205	CAB	0.949	0.999	1.000	1.000	
AGT rs699 M235T	C/T	DKD	European/Asian	21	3722/3539	allelic model	1.15 (0.99 , 1.33)	0.059	73.39%	0.252	0.917	0.832	ACB	0.911	0.998	1.000	1.000	Credible
				21	3722/3539	dominant model	1.12 (0.94 , 1.34)	0.212	58.70%	0.257	0.600	0.198	CCB	0.954	0.999	1.000	1.000	
				21	3722/3539	recessive model	1.32 (1.04 , 1.68)	0.022	67.21%	0.129	0.872	0.975	ACB	0.847	0.997	1.000	1.000	
				21	3722/3539	heterozygous model	1.02 (0.88 , 1.19)	0.786	38.88%	0.352	0.661	0.038	CBB	0.966	0.999	1.000	1.000	
				21	3722/3539	homozygous model	1.41 (1.06 , 1.87)	0.020	65.18%	0.292	0.841	0.984	ACB	0.813	0.996	1.000	1.000	
			European	15	2421/2557	allelic model	1.07 (0.98 , 1.17)	0.154	16.31%	0.035	0.772	0.300	CAC	0.944	0.999	1.000	1.000	Not credible
				15	2421/2557	dominant model	1.05 (0.92 , 1.2)	0.452	10.38%	0.245	0.658	0.115	CAB	0.965	0.999	1.000	1.000	
				15	2421/2557	recessive model	1.18 (0.96 , 1.46)	0.112	39.23%	0.008	0.719	0.379	CBC	0.941	0.999	1.000	1.000	
				15	2421/2557	heterozygous model	1.01 (0.87 , 1.17)	0.892	20.26%	0.695	1.000	0.034	CAB	0.965	0.999	1.000	1.000	
				15	2421/2557	homozygous model	1.19 (0.96 , 1.46)	0.112	25.76%	0.013	0.557	0.411	CBC	0.932	0.999	1.000	1.000	
			Asian	6	1301/982	allelic model	1.31 (0.78 , 2.2)	0.316	91.00%	0.905	0.739	0.913	ACB	0.960	0.999	1.000	1.000	Not credible
				6	1301/982	dominant model	1.43 (0.68 , 3.02)	0.346	84.74%	0.967	0.387	0.128	CCB	0.962	0.999	1.000	1.000	
				6	1301/982	recessive model	1.53 (0.82 , 2.87)	0.180	83.02%	0.154	0.871	0.997	ACB	0.951	0.999	1.000	1.000	
				6	1301/982	heterozygous model	1.15 (0.66 , 2.01)	0.614	65.89%	0.761	0.462	0.092	CCB	0.966	0.999	1.000	1.000	
				6	1301/982	homozygous model	1.92 (0.78 , 4.74)	0.156	78.35%	0.366	0.789	1.000	ACB	0.947	0.999	1.000	1.000	
AGT rs699 T174M	T/C	DKD	European/Asian/African	5	915/821	allelic model	1.01 (0.82 , 1.24)	0.926	0.00%	0.262	1.000	0.030	CAB	0.965	0.999	1.000	1.000	Not credible
				4	855/721	dominant model	0.97 (0.73 , 1.28)	0.817	14.64%	0.341	1.000	0.049	CAB	0.962	0.999	1.000	1.000	
				4	855/721	recessive model	2.24 (0.67 , 7.43)	0.189	25.47%	0.955	1.000	0.431	CBB	0.951	0.999	1.000	1.000	
				4	855/721	heterozygous model	0.9 (0.7 , 1.15)	0.395	1.87%	0.634	1.000	0.127	CAB	0.952	0.999	1.000	1.000	
				4	855/721	homozygous model	2.19 (0.66 , 7.28)	0.200	25.23%	0.977	1.000	0.373	CBB	0.953	0.999	1.000	1.000	
			European	3	332/375	allelic model	1 (0.72 , 1.37)	0.980	1.23%	0.776	1.000	0.022	CAB	0.964	0.999	1.000	1.000	Not credible
				7	1556/1616	allelic model	1.12 (0.93 , 1.34)	0.243	39.63%	0.537	0.724	0.426	CBB	0.954	0.999	1.000	1.000	

AGTR1 rs5186	C/A	DKD	European/Asian	8	2301/2023	dominant model	1.08 (0.87 , 1.33)	0.494	47.82%	0.927	1.000	0.166	CBB	0.964	0.999	1.000	1.000	Not credible
				7	1556/1616	recessive model	1.19 (0.59 , 2.42)	0.631	61.22%	0.290	0.841	0.332	CCB	0.966	0.999	1.000	1.000	
				7	1556/1616	heterozygous model	1.1 (0.83 , 1.46)	0.521	58.26%	0.963	0.472	0.217	CCB	0.965	0.999	1.000	1.000	
				7	1556/1616	homozygous model	1.21 (0.59 , 2.48)	0.594	59.73%	0.313	0.831	0.339	CCB	0.966	0.999	1.000	1.000	
			European	4	380/581	allelic model	1.01 (0.76 , 1.36)	0.937	47.26%	0.068	1.000	0.026	CBC	0.965	0.999	1.000	1.000	Not credible
				4	380/581	dominant model	1.04 (0.64 , 1.68)	0.879	68.15%	0.311	1.000	0.022	CCB	0.965	0.999	1.000	1.000	
				4	380/581	recessive model	0.97 (0.51 , 1.83)	0.920	41.10%	0.415	1.000	0.029	CBB	0.963	0.999	1.000	1.000	
				4	380/581	heterozygous model	1.06 (0.59 , 1.91)	0.840	75.66%	0.387	0.411	0.023	CCB	0.965	0.999	1.000	1.000	
				4	380/581	homozygous model	0.96 (0.53 , 1.74)	0.901	26.47%	0.872	1.000	0.028	CBB	0.963	0.999	1.000	1.000	
AKR1B1 rs759853	T/C	DKD	European	7	1124/913	allelic model	1.54 (1.16 , 2.04)	0.003	75.14%	0.002	0.313	0.996	ACC	0.507	0.982	0.998	1.000	Credible
				7	1124/913	dominant model	1.84 (1.27 , 2.64)	0.001	69.74%	0.009	0.303	0.999	ACC	0.307	0.959	0.996	1.000	
				7	1124/913	recessive model	1.42 (0.97 , 2.08)	0.072	50.64%	0.004	0.891	0.535	BCC	0.920	0.998	1.000	1.000	
				7	1124/913	homozygous model	2.01 (1.21 , 3.31)	0.007	64.77%	0.003	0.376	0.965	ACC	0.667	0.991	0.999	1.000	
				7	1124/913	codominant model	1.42 (1.12 , 1.79)	0.004	34.02%	0.036	0.716	0.911	ABC	0.534	0.984	0.998	1.000	
CCL5 rs2107538	A/G	DKD	European/Asian	4	797/1064	dominant model	1.03 (0.79 , 1.34)	0.827	39.04%	0.548	1.000	0.038	CBB	0.966	0.999	1.000	1.000	Not credible
CCR5 rs1799987	A/G	DKD	European/Asian	9	2196/2094	dominant model	1.92 (1.29 , 2.84)	0.001	82.06%	0.080	0.899	1.000	ACC	0.335	0.964	0.996	1.000	Credible
CYP11B2 rs1799998	T/C	DKD	European/Asian	5	825/910	allelic model	1.28 (0.99 , 1.65)	0.056	56.28%	0.764	0.569	0.733	BCB	0.908	0.998	1.000	1.000	Not credible
				5	825/910	dominant model	1.38 (0.93 , 2.04)	0.113	26.00%	0.363	0.709	0.386	CBB	0.935	0.999	1.000	1.000	
				5	825/910	recessive model	1.31 (1 , 1.73)	0.052	36.01%	0.989	0.802	0.655	BBB	0.909	0.998	1.000	1.000	
				5	825/910	heterozygous model	1.16 (0.87 , 1.56)	0.301	0.00%	0.358	1.000	0.172	CAB	0.961	0.999	1.000	1.000	
				5	825/910	homozygous model	1.59 (0.96 , 2.64)	0.070	43.12%	0.604	0.426	0.572	BBB	0.921	0.998	1.000	1.000	
eNOS rs1799983	T/G	DKD	European/Asian	19	4586/4201	allelic model	1.2 (1.01 , 1.43)	0.042	81.65%	0.116	0.159	0.964	ACB	0.890	0.998	1.000	1.000	Credible
				18	4434/4006	dominant model	1.26 (1.01 , 1.57)	0.037	79.08%	0.077	0.559	0.831	ACC	0.887	0.998	1.000	1.000	
				18	4398/4110	recessive model	1.26 (0.96 , 1.64)	0.092	55.79%	0.402	0.058	0.928	ACB	0.927	0.999	1.000	1.000	
				18	4434/4006	codominant model	1.08 (0.92 , 1.28)	0.328	60.22%	0.138	0.786	0.090	CCB	0.962	0.999	1.000	1.000	
			European	13	3562/3090	allelic model	1.22 (1.02 , 1.46)	0.027	78.33%	0.552	0.223	0.991	ACB	0.866	0.997	1.000	1.000	Credible
				13	3562/3090	dominant model	1.24 (1 , 1.53)	0.046	72.47%	0.388	0.719	0.878	ACB	0.895	0.998	1.000	1.000	
				14	3609/3253	recessive model	1.31 (1 , 1.71)	0.046	56.97%	0.195	0.099	0.955	ACB	0.898	0.998	1.000	1.000	
				13	3562/3090	codominant model	1.04 (0.92 , 1.16)	0.534	16.44%	0.781	1.000	0.074	CAB	0.965	0.999	1.000	1.000	
eNOS rs2070744	C/T	DKD	European/Asian	9	2426/2519	allelic model	1.24 (1.09 , 1.41)	0.001	50.53%	0.910	0.916	0.995	ACB	0.325	0.962	0.996	1.000	Credible
				8	2274/2324	dominant model	1.27 (1.09 , 1.48)	0.002	33.50%	0.792	0.776	0.975	ABB	0.472	0.979	0.998	1.000	
				8	2274/2324	recessive model	1.54 (1 , 2.38)	0.050	69.91%	0.010	0.625	0.793	BCC	0.903	0.998	1.000	1.000	
				8	2274/2324	codominant model	1.16 (1.02 , 1.31)	0.019	0.00%	0.290	0.286	0.640	BAB	0.811	0.996	1.000	1.000	
			European	6	1688/1742	allelic model	1.27 (1.05 , 1.53)	0.013	65.45%	0.685	0.847	0.991	ACB	0.769	0.994	0.999	1.000	Credible
				6	1688/1742	dominant model	1.32 (1.09 , 1.6)	0.005	43.46%	0.595	0.736	0.978	ABB	0.620	0.988	0.999	1.000	
				6	1688/1742	recessive model	1.62 (0.95 , 2.77)	0.078	77.25%	0.011	0.538	0.765	BCC	0.924	0.998	1.000	1.000	
				6	1688/1742	codominant model	1.18 (1.01 , 1.36)	0.032	9.03%	0.188	0.258	0.629	BAB	0.840	0.996	1.000	1.000	
			European/Asian	21	4895/4976	allelic model	1.16 (1.01 , 1.32)	0.030	57.37%	0.851	0.844	0.911	ACB	0.849	0.997	1.000	1.000	Credible
				21	4895/4976	dominant model	1.09 (0.98 , 1.22)	0.116	23.75%	0.487	0.304	0.406	CAB	0.943	0.999	1.000	1.000	
				18	4672/4725	recessive model	1.61 (1.04 , 2.49)	0.034	62.97%	0.889	0.977	0.999	ACB	0.872	0.997	1.000	1.000	

eNOS rs869109213	4a/4b	DKD		21	4895/4976	codominant model	1.000 (0.88 , 1.12)	0.860	29.63%	0.377	0.536	0.054	CBB	0.962	0.999	1.000	1.000	
			European	10	3639/3289	allelic model	1.17 (1 , 1.37)	0.056	68.10%	1.000	0.986	0.905	ACB	0.903	0.998	1.000	1.000	Not credible
				10	3639/3289	dominant model	1.09 (0.98 , 1.21)	0.117	2.06%	0.795	0.576	0.341	CAB	0.935	0.999	1.000	1.000	
				10	3639/3289	recessive model	1.7 (0.98 , 2.94)	0.059	76.83%	0.918	0.961	0.999	ACB	0.909	0.998	1.000	1.000	
				10	3639/3289	codominant model	0.96 (0.84 , 1.1)	0.537	30.99%	0.492	0.694	0.106	CBB	0.956	0.999	1.000	1.000	
ENPP1 rs1044498	C/A	DKD	European	4	1102/1460	allelic model	1.27 (1.04 , 1.55)	0.017	40.03%	0.434	0.485	0.903	ABB	0.823	0.996	1.000	1.000	Credible
				4	1102/1460	dominant model	1.27 (1.01 , 1.61)	0.042	42.71%	0.652	0.802	0.797	BBB	0.899	0.998	1.000	1.000	
				4	1102/1460	recessive model	1.6 (0.89 , 2.87)	0.118	42.42%	0.070	0.694	0.659	BBC	0.938	0.999	1.000	1.000	
				4	1102/1460	homozygous model	1.68 (0.93 , 3.02)	0.083	41.78%	0.090	0.729	0.726	BBC	0.926	0.998	1.000	1.000	
GLUT1 Xbαl	1.1/0.9	DKD	European/Asian	6	710/750	allelic model	1.29 (0.94 , 1.78)	0.116	71.97%	0.140	0.406	0.567	BCB	0.940	0.999	1.000	1.000	Not credible
				6	710/750	dominant model	1.49 (0.89 , 2.48)	0.128	77.90%	0.077	0.484	0.470	CCC	0.941	0.999	1.000	1.000	
				6	710/750	recessive model	1.3 (0.77 , 2.18)	0.326	50.94%	0.905	0.360	0.324	CCB	0.961	0.999	1.000	1.000	
				6	710/750	homozygous model	1.54 (0.81 , 2.93)	0.185	61.46%	0.288	0.552	0.431	CCB	0.951	0.999	1.000	1.000	
			European	5	646/705	allelic model	1.21 (0.86 , 1.7)	0.267	73.27%	0.272	0.592	0.346	CCB	0.958	0.999	1.000	1.000	Not credible
				5	646/705	dominant model	1.23 (0.77 , 1.98)	0.389	72.25%	0.219	0.529	0.179	CCB	0.963	0.999	1.000	1.000	
				5	646/705	recessive model	1.34 (0.75 , 2.41)	0.318	60.10%	0.638	0.390	0.344	CCB	0.961	0.999	1.000	1.000	
				5	646/705	homozygous model	1.48 (0.73 , 3)	0.280	68.00%	0.353	0.508	0.355	CCB	0.959	0.999	1.000	1.000	
ICAM1 rs5498	G/A	DKD	European/Asian	6	1767/1709	allelic model	0.87 (0.71 , 1.06)	0.168	72.67%	0.718	0.125	0.781	BCB	0.948	0.999	1.000	1.000	Not credible
				6	1767/1709	dominant model	0.9 (0.65 , 1.24)	0.525	67.74%	0.077	0.518	0.636	BCC	0.955	0.999	1.000	1.000	
				6	1767/1709	recessive model	0.75 (0.46 , 1.21)	0.236	83.28%	0.890	0.571	0.654	BCB	0.949	0.999	1.000	1.000	
				6	1767/1709	homozygous model	0.81 (0.5 , 1.31)	0.393	72.27%	0.778	0.282	0.377	CCB	0.952	0.999	1.000	1.000	
SERPINE1 rs1799889	4G/5G	DKD	European/Asian/African	15	1730/1508	allelic model	1.48 (1.15 , 1.9)	0.002	82.96%	0.139	0.957	1.000	ACB	0.463	0.978	0.998	1.000	Credible
				15	1730/1508	dominant model	1.41 (1.01 , 1.97)	0.045	69.76%	0.705	0.914	0.957	ACB	0.894	0.998	1.000	1.000	
				15	1730/1508	recessive model	1.78 (1.27 , 2.51)	0.001	76.63%	0.001	0.611	1.000	ACC	0.321	0.961	0.996	1.000	
				15	1730/1508	heterozygous model	1.13 (0.83 , 1.53)	0.431	52.72%	0.857	0.622	0.292	CCB	0.964	0.999	1.000	1.000	
				15	1730/1508	homozygous model	1.92 (1.26 , 2.95)	0.003	74.19%	0.143	0.942	1.000	ACB	0.528	0.983	0.998	1.000	
SOD2 rs4880	C/T	DKD	European/Asian	11	2269/1932	allelic model	0.81 (0.66 , 0.99)	0.043	70.78%	0.108	0.549	0.770	BCB	0.950	0.999	1.000	1.000	Credible
				11	2269/1932	dominant model	0.73 (0.56 , 0.96)	0.026	60.20%	0.799	0.424	0.939	ACB	0.948	0.999	1.000	1.000	
				11	2269/1932	recessive model	0.92 (0.71 , 1.2)	0.548	40.24%	0.122	0.819	0.096	CBB	0.956	0.999	1.000	1.000	
TGF-β1 rs1800469	T/C	DKD	European/Asian	4	902/888	allelic model	0.98 (0.85 , 1.14)	0.839	2.46%	0.537	1.000	0.037	CAB	0.961	0.999	1.000	1.000	Not credible
				4	902/888	dominant model	1.04 (0.85 , 1.26)	0.718	0.20%	0.801	1.000	0.050	CAB	0.966	0.999	1.000	1.000	
				4	902/888	recessive model	0.84 (0.59 , 1.2)	0.349	15.87%	0.971	1.000	0.160	CAB	0.951	0.999	1.000	1.000	
TGF-β1 rs1800468	A/G	DKD	European/Asian	4	882/922	allelic model	1.04 (0.82 , 1.33)	0.737	6.25%	0.675	1.000	0.054	CAB	0.966	0.999	1.000	1.000	Not credible
				4	882/922	dominant model	1.08 (0.82 , 1.41)	0.593	6.50%	0.891	1.000	0.077	CAB	0.966	0.999	1.000	1.000	
				4	882/922	recessive model	0.79 (0.31 , 2.03)	0.620	7.48%	0.286	1.000	0.052	CAB	0.958	0.999	1.000	1.000	
TGF-β1 rs1800471	C/G	DKD	European/Asian	5	686/757	allelic model	1.18 (0.8 , 1.75)	0.397	35.18%	0.212	0.579	0.162	CBB	0.963	0.999	1.000	1.000	Not credible
				5	686/757	dominant model	1.21 (0.79 , 1.84)	0.378	29.68%	0.359	0.545	0.175	CBB	0.962	0.999	1.000	1.000	
				4	637/707	recessive model	1.04 (0.48 , 2.26)	0.912	1.98%	0.686	1.000	0.022	CAB	0.965	0.999	1.000	1.000	
				11	2039/2201	allelic model	1.13 (0.87 , 1.48)	0.357	73.83%	0.107	0.687	0.988	ACB	0.962	0.999	1.000	1.000	
				10	2015/2178	dominant model	1.16 (0.83 , 1.61)	0.390	76.35%	0.151	0.688	0.980	ACB	0.962	0.999	1.000	1.000	

TNF-α rs1800629	A/G	DKD	European/Asian	9	1893/2053	recessive model	1.32 (0.87 , 1.99)	0.188	28.67%	0.470	0.760	0.575	BBB	0.951	0.999	1.000	1.000	Not credible
				10	2015/2178	heterozygous model	1.14 (0.82 , 1.59)	0.441	73.19%	0.218	0.859	0.935	ACB	0.964	0.999	1.000	1.000	
				9	1893/2053	homozygous model	1.43 (0.86 , 2.37)	0.164	45.37%	0.398	0.876	0.807	ABB	0.948	0.999	1.000	1.000	
			European	4	574/850	allelic model	1.03 (0.82 , 1.3)	0.810	17.24%	0.585	1.000	0.031	CAB	0.966	0.999	1.000	1.000	Not credible
				4	574/850	dominant model	1.04 (0.82 , 1.33)	0.729	3.13%	0.496	1.000	0.046	CAB	0.966	0.999	1.000	1.000	
				4	574/850	recessive model	0.87 (0.2 , 3.86)	0.856	78.84%	0.837	1.000	0.042	CCB	0.962	0.999	1.000	1.000	
				4	574/850	heterozygous model	1.07 (0.82 , 1.39)	0.639	7.11%	0.539	1.000	0.061	CAB	0.966	0.999	1.000	1.000	
				4	574/850	homozygous model	0.91 (0.21 , 3.9)	0.894	77.11%	0.852	1.000	0.044	CCB	0.963	0.999	1.000	1.000	
UCP2 I/D	I/D	DKD	European/Asian	4	638/724	allelic model	0.94 (0.75 , 1.17)	0.589	30.97%	0.577	1.000	0.075	CBB	0.957	0.999	1.000	1.000	Not credible
				5	854/942	dominant model	0.88 (0.73 , 1.06)	0.188	0.00%	0.995	1.000	0.245	CAB	0.948	0.999	1.000	1.000	
				4	638/724	recessive model	1.03 (0.57 , 1.85)	0.921	37.63%	0.298	1.000	0.069	CBB	0.965	0.999	1.000	1.000	
				4	638/724	homozygous model	0.96 (0.53 , 1.73)	0.883	35.19%	0.365	1.000	0.027	CBB	0.963	0.999	1.000	1.000	
UCP2 rs659366	A/G	DKD	European/Asian	4	670/697	allelic model	0.94 (0.73 , 1.21)	0.615	57.28%	0.954	0.633	0.088	CCB	0.958	0.999	1.000	1.000	Not credible
				4	670/697	dominant model	0.9 (0.63 , 1.29)	0.570	59.39%	0.829	0.658	0.129	CCB	0.957	0.999	1.000	1.000	
				4	670/697	recessive model	0.99 (0.71 , 1.37)	0.942	11.66%	0.572	1.000	0.023	CAB	0.964	0.999	1.000	1.000	
				4	670/697	homozygous model	0.93 (0.6 , 1.42)	0.723	34.67%	0.668	1.000	0.043	CBB	0.960	0.999	1.000	1.000	
VDR rs7975232	A/C	DKD	European/Asian	6	1181/1212	allelic model	0.97 (0.81 , 1.17)	0.773	40.69%	0.852	0.633	0.069	CBB	0.960	0.999	1.000	1.000	Not credible
				6	1181/1212	dominant model	1.01 (0.81 , 1.25)	0.963	20.09%	0.811	0.416	0.024	CAB	0.965	0.999	1.000	1.000	
				6	1181/1212	recessive model	0.91 (0.62 , 1.32)	0.608	27.51%	0.403	0.618	0.138	CBB	0.958	0.999	1.000	1.000	
				6	1181/1212	heterozygous model	1.04 (0.86 , 1.25)	0.715	0.00%	0.751	1.000	0.051	CAB	0.966	0.999	1.000	1.000	
				6	1181/1212	homozygous model	0.9 (0.58 , 1.42)	0.660	35.99%	0.421	0.647	0.096	CBB	0.958	0.999	1.000	1.000	
VDR rs1544410	T/C	DKD	European/Asian	5	1126/1079	allelic model	1.41 (1.01 , 1.98)	0.047	66.60%	0.013	0.293	0.518	BCC	0.898	0.998	1.000	1.000	Credible
				5	1126/1079	dominant model	1.38 (0.98 , 1.95)	0.064	51.51%	0.012	0.515	0.445	CCC	0.917	0.998	1.000	1.000	
				4	1078/1034	recessive model	1.33 (0.66 , 2.68)	0.427	68.48%	0.762	0.825	0.231	CCB	0.964	0.999	1.000	1.000	
				5	1126/1079	heterozygous model	1.25 (0.69 , 2.28)	0.464	74.14%	0.958	0.176	0.180	CCB	0.964	0.999	1.000	1.000	
				4	1078/1034	homozygous model	1.3 (0.64 , 2.61)	0.469	66.66%	0.678	0.798	0.208	CCB	0.964	0.999	1.000	1.000	
VDR rs2228570	C/T	DKD	European/Asian	5	1068/1072	allelic model	0.87 (0.6 , 1.27)	0.476	83.17%	0.103	0.714	0.025	CCB	0.954	0.999	1.000	1.000	Not credible
				5	1068/1072	dominant model	0.87 (0.49 , 1.55)	0.636	74.90%	0.133	0.536	0.049	CCB	0.958	0.999	1.000	1.000	
				5	1068/1072	recessive model	0.87 (0.56 , 1.37)	0.551	73.49%	0.158	0.523	0.046	CCB	0.956	0.999	1.000	1.000	
				5	1068/1072	heterozygous model	0.96 (0.61 , 1.51)	0.852	55.80%	0.130	0.702	0.080	CCB	0.962	0.999	1.000	1.000	
				5	1068/1072	homozygous model	0.79 (0.37 , 1.67)	0.534	80.95%	0.129	0.662	0.030	CCB	0.956	0.999	1.000	1.000	
VDR rs731236	T/C	DKD	European/Asian	5	1000/1090	allelic model	0.93 (0.65 , 1.33)	0.684	57.73%	0.716	0.324	0.150	CCB	0.959	0.999	1.000	1.000	Not credible
				4	952/1045	dominant model	0.92 (0.41 , 2.04)	0.832	71.67%	0.584	1.000	0.088	CCB	0.962	0.999	1.000	1.000	
				5	1000/1090	recessive model	0.96 (0.55 , 1.68)	0.886	60.80%	0.873	0.818	0.130	CCB	0.963	0.999	1.000	1.000	
				4	952/1045	heterozygous model	0.9 (0.5 , 1.6)	0.714	48.01%	0.734	1.000	0.074	CBB	0.960	0.999	1.000	1.000	
				4	952/1045	homozygous model	1.14 (0.44 , 2.98)	0.789	55.60%	0.482	1.000	0.059	CCB	0.966	0.999	1.000	1.000	
ACE Ins/Del	Del/Ins	DPN	European/Asian	7	824/1602	dominant model	1.2 (0.96 , 1.5)	0.110	0.00%	0.044	1.000	1.000	AAC	0.936	0.999	1.000	1.000	Not credible
				4	535/1036	heterozygous model	1.44 (0.64 , 3.26)	0.379	83.99%	0.650	0.617	0.950	ACB	0.963	0.999	1.000	1.000	
				4	535/1036	homozygous model	1.35 (0.48 , 3.77)	0.570	85.35%	0.276	0.916	0.638	BCB	0.966	0.999	1.000	1.000	
			European	6	803/1539	dominant model	1.18 (0.95 , 1.48)	0.139	0.00%	0.087	1.000	0.998	AAC	0.946	0.999	1.000	1.000	Not credible

CCR5 rs1799987	A/G	Microvascular complications	European/Asian	10	2119/2251	dominant model	1.77 (1.06 , 2.97)	0.029	90.96%	0.845	0.842	1.000	ACB	0.868	0.997	1.000	1.000	Credible
			European	4	1123/1088	dominant model	1.09 (0.47 , 2.53)	0.842	92.56%	0.388	0.796	0.368	CCB	0.965	0.999	1.000	1.000	Not credible
EPO rs1617640	T/G	Microvascular complications	European/Asian	8	2848/2124	allelic model	1.14 (0.94 , 1.39)	0.180	76.44%	0.006	0.339	0.999	ACC	0.952	0.999	1.000	1.000	Credible
				8	2848/2124	dominant model	1.29 (0.99 , 1.69)	0.056	49.66%	0.046	0.663	0.879	ABC	0.915	0.998	1.000	1.000	
				8	2848/2124	recessive model	1.26 (0.97 , 1.63)	0.087	72.25%	0.168	0.331	0.998	ACB	0.924	0.998	1.000	1.000	
				8	2848/2124	heterozygous model	1.17 (0.94 , 1.45)	0.152	22.81%	0.101	0.292	0.372	CAB	0.946	0.999	1.000	1.000	
				8	2848/2124	homozygous model	1.55 (1.1 , 2.19)	0.013	56.11%	0.000	0.264	0.999	ACC	0.780	0.995	0.999	1.000	
ICAM1 rs5498	G/A	Microvascular complications	European/Asian	7	1810/1601	dominant model	0.73 (0.56 , 0.96)	0.024	61.52%	0.056	0.502	0.886	ACC	0.948	0.999	1.000	1.000	Credible
				7	1810/1601	recessive model	0.98 (0.73 , 1.31)	0.888	56.10%	0.148	0.408	0.054	CCB	0.963	0.999	1.000	1.000	
				7	1810/1601	additive model	1.25 (1.09 , 1.43)	0.001	0.00%	0.318	0.853	0.890	AAB	0.345	0.965	0.996	1.000	
			European	3	1053/995	dominant model	0.91 (0.62 , 1.34)	0.650	66.82%	0.581	1.000	0.267	CCB	0.958	0.999	1.000	1.000	Credible
				3	1053/995	recessive model	1.29 (0.88 , 1.91)	0.196	54.53%	0.580	0.711	0.404	CCB	0.953	0.999	1.000	1.000	
				3	1053/995	additive model	1.27 (1.06 , 1.51)	0.008	0.55%	0.967	0.723	0.742	BAB	0.686	0.991	0.999	1.000	
SOD2 rs4880	C/T	Microvascular complications	European/Asian	21	3426/3100	allelic model	0.73 (0.62 , 0.86)	1.42E-04	72.17%	0.039	0.149	1.000	ACC	0.501	0.981	0.998	1.000	Credible
				21	3426/3100	dominant model	0.65 (0.52 , 0.81)	1.24E-04	61.06%	0.006	0.040	1.000	ACC	0.438	0.976	0.998	1.000	
				21	3426/3100	recessive model	0.72 (0.56 , 0.92)	0.008	61.13%	0.131	0.126	0.938	ACB	0.933	0.999	1.000	1.000	
HIF1A rs11549465	C/T	All vsacular complications	European/Asian	5	1494/1075	allelic model	1.41 (1.13 , 1.75)	0.002	19.14%	0.954	0.214	0.945	AAB	0.435	0.976	0.998	1.000	Credible
				3	1205/821	dominant model	2.46 (1.48 , 4.1)	0.001	7.97%	0.404	0.918	0.948	AAB	0.222	0.938	0.993	1.000	
				5	1494/1075	recessive model	1.28 (0.97 , 1.7)	0.080	28.89%	0.790	0.289	0.705	BBB	0.929	0.999	1.000	1.000	
				3	1205/821	heterozygous model	2.43 (1.13 , 5.2)	0.023	45.85%	0.146	0.847	0.944	ABB	0.840	0.996	1.000	1.000	
				3	1205/821	homozygous model	1.97 (1.16 , 3.36)	0.012	0.35%	0.893	1.000	0.646	BAB	0.779	0.995	0.999	1.000	

A1/A2, alternative allele and reference allele for the genetic variants; allelic model, A1 vs. A2, dominant model, A1A1+A1A2 vs. A2A2, recessive model, A1A1 vs. A1A2+A2A2, heterozygous model, A1A2 vs A2A2, homozygous model, A1A1 vs A2A2, codominant model, A1A2 vs. A1A1+A2A2, additive model, A1A1+A2A2 vs. A1A2; P.effect, the P-value for meta-analyses effects; P.Egger, the P-value for small study effects; P.Sig, the P-value for excess significance; BFDP1, BFDP2, BFDP3, and BFDP4 were respectively calculated at the prior level of 0.05, 10-3, 10-4, and 10-6. DR, diabetic retinopathy; PDR, proliferative diabetic retinopathy; NPDR, non-proliferative diabetic retinopathy; DKD, diabetic kidney disease; DPN, diabetic peripheral neuropathy.

Supplementary Table 6. Characteristics of the latest GWASs for DR and DKD.

Author-year	Title	Type of DM	Population	Outcome	N (cases)	N (controls)	N (total)	Summary of findings
Pollack-2019	Multiethnic Genome-Wide Association Study of Diabetic Retinopathy Using Liability Threshold Modeling of Duration of Diabetes and Glycemic Control	2	European	Any DR	1079	1970	3049	Null for genome-wide significance (P<5E-8) and multiple test corrections of different traits (P<6.25E-9)
				NPDR	644	1970	2614	Null for genome-wide significance (P<5E-8) and multiple test corrections of different traits (P<6.25E-9)
				PDR	398	1970	2368	1 loci for genome-wide significance (P<5E-8), null for multiple test corrections of different traits (P<6.25E-9)
Sandholm-2022	Genome-wide meta-analysis and omics integration identifies novel genes associated with diabetic kidney disease	Mixed	European	Any DKD	11380	15405	26785	1 loci for genome-wide significance (P<5E-8), null for multiple test corrections of different traits (P<9.3E-9)
				Microalbuminuria	4779	15331	20110	3 loci for genome-wide significance (P<5E-8), 1 loci for multiple test corrections of different traits (P<9.3E-9)
				Macroalbuminuria	3528	14448	17976	Null for genome-wide significance (P<5E-8) and multiple test corrections of different traits (P<9.3E-9)
				ESRD	2717	14962	17679	1 loci for genome-wide significance (P<5E-8), null for multiple test corrections of different traits (P<9.3E-9)
				SevereDKD	6705	15430	22134	2 loci for genome-wide significance (P<5E-8), 1 loci for multiple test corrections of different traits (P<9.3E-9)
				CKD	7636	18576	26212	2 loci for genome-wide significance (P<5E-8), null for multiple test corrections of different traits (P<9.3E-9)
				CKD extremes	2660	18163	20823	Null for genome-wide significance (P<5E-8) and multiple test corrections of different traits (P<9.3E-9)
				CKD-DKD	4122	13972	18094	2 loci for genome-wide significance (P<5E-8), null for multiple test corrections of different traits (P<9.3E-9)

The diagnosis of diabetic retinopathy (DR) were based on the Early Treatment Diabetic Retinopathy Study (ETDRS) score. Any DR was defined as ETDRS ≥ 14 vs. ETDRS < 14, non-proliferative diabetic retinopathy (NPDR) was defined as ETDRS ≥ 30 vs. ETDRS < 14, proliferative diabetic retinopathy (PDR) was defined as ETDRS ≥ 60 vs. ETDRS < 14.

The diagnosis of diabetic kidney disease (DKD) was based on albumin excretion rate (AER) and estimated glomerular filtration rate (eGFR). Any DKD was defined as microalbuminuria or macroalbuminuria or end-stage renal disease (ESRD) vs. normal AER; Microalbuminuria was defined as microalbuminuria vs. normal AER; Macroalbuminuria was defined as macroalbuminuria vs. normal AER; ESRD was defined as ESRD vs. normal AER; Severe DKD was defined as macroalbuminuria or ESRD vs. normal AER; CKD was defined as eGFR < 60 ml/in/1.73m2 vs. eGFR ≥ 60 ml/in/1.73m2; CKD extremes was defined as eGFR < 15 ml/in/1.73m2 vs. eGFR ≥ 60 ml/in/1.73m2; CKD-DKD was defined as ESRD, or eGFR < 60 ml/in/1.73m2 AND microalbuminuria or macroalbuminuria vs. normal AER and eGFR ≥ 60 ml/in/1.73m2.

Supplementary Table 7. Summary of GWAS estimates for "highly credible" and "credible" associations in DR.

Chr:Pos	Variant	Credibility in umbrella review	Ref_Allele	Alt_Allele	Any DR			NPDR			PDR		
					Effect	StdErr	P-value	Effect	StdErr	P-value	Effect	StdErr	P-value
1:11854476	MTHFR rs1801131	Credible	T	G	0.099	0.063	0.119	0.077	0.103	0.454	0.068	0.407	0.868
1:11856378	MTHFR rs1801133	Credible	G	A	-0.130	0.113	0.249	-0.160	0.098	0.103	-0.093	0.089	0.295
6:32152387	AGER rs1800624	Credible	A	T	-0.068	0.031	0.031	-0.106	0.067	0.114	-0.005	0.003	0.171
6:43736389	VEGF rs699947	Credible	A	C	0.022	0.018	0.231	-0.030	0.050	0.555	-0.014	0.019	0.452
6:43737794	VEGF rs13207351	Credible	A	G	0.020	0.016	0.222	-0.036	0.071	0.613	-0.031	0.052	0.543
6:43738350	VEGF rs2010963	Credible	C	G	-0.030	0.035	0.399	0.006	0.009	0.453	0.001	0.002	0.718
6:43745095	VEGF rs2146323	Credible	C	A	-0.011	0.018	0.533	0.016	0.202	0.937	-0.019	0.036	0.593
6:43752536	VEGF rs3025039	Highly credible	C	T	-0.040	0.472	0.932	-0.085	0.264	0.747	-0.094	0.673	0.889
7:134143958	AKR1B1 rs759853	Credible	G	A	-0.008	0.010	0.429	-0.044	0.026	0.098	-0.042	0.047	0.379
7:22766645	IL-6 rs1800795	Credible	C	G	-0.043	0.086	0.615	-0.076	0.067	0.254	-0.101	0.073	0.166
9:120475302	TLR4 rs4986790	Credible	A	G	-0.027	0.053	0.613	-0.041	0.200	0.836	0.313	0.222	0.158
10:114758349	TCF7L2 rs7903146	Credible	C	T	-0.093	0.285	0.745	-0.093	0.092	0.311	-0.025	NA	1.000
17:32579788	MCP-1 rs1024611	Highly credible	A	G	0.092	0.148	0.535	0.122	0.326	0.708	0.149	0.427	0.728
19:10395683	ICAM1 rs5498	Credible	A	G	-0.042	0.025	0.101	-0.033	0.021	0.117	-0.129	0.082	0.115
19:41860296	TGF-β1 rs1800469	Credible	A	G	-0.039	0.200	0.846	0.036	0.045	0.429	0.036	0.072	0.620

SERPINE1 rs1799889 was not available in the GWAS summary statistics. StdErr, standard error.

Supplementary Table 8. Summary of GWAS estimates for "highly credible" and "credible" associations in DKD.

Chr:Pos	Variant	Credibility in umbrella review	Ref_Allele	Alt_Allele	Any DKD			Microalbuminuria			Macroalbuminuria			ESRD			Severe DKD			CKD			CKD extremes			CKD-DKD		
					Effect	StdErr	P-value	Effect	StdErr	P-value	Effect	StdErr	P-value	Effect	StdErr	P-value	Effect	StdErr	P-value	Effect	StdErr	P-value	Effect	StdErr	P-value	Effect	StdErr	P-value
1:11856378	MTHFR rs1801133	Highly credible	T	A	0.006	0.021	0.777	-0.014	0.029	0.617	0.008	0.033	0.805	0.055	0.038	0.147	0.020	0.026	0.448	0.045	0.025	0.072	0.044	0.037	0.232	0.041	0.033	0.211
1:230845794	AGT rs699 M235T/T174M	Credible	G	A	0.005	0.020	0.800	-0.034	0.027	0.214	0.044	0.031	0.154	0.002	0.035	0.959	0.026	0.024	0.290	0.033	0.023	0.165	0.011	0.034	0.745	0.001	0.031	0.974
3:12393125	PPARγ rs1801282	Credible	G	C	-0.026	0.030	0.388	0.028	0.041	0.489	-0.068	0.046	0.139	-0.091	0.052	0.078	-0.072	0.036	0.043	-0.048	0.034	0.163	-0.077	0.051	0.128	-0.060	0.045	0.182
3:148459988	AGTR1 rs5186	Credible	C	A	-0.048	0.024	0.046	-0.036	0.032	0.266	0.003	0.037	0.933	-0.062	0.043	0.146	-0.056	0.029	0.053	-0.038	0.028	0.178	-0.036	0.042	0.387	-0.057	0.037	0.121
3:186559460	ADIPOQ rs17300539	Credible	G	A	0.083	0.042	0.045	0.047	0.057	0.403	0.111	0.065	0.089	-0.041	0.090	0.652	0.090	0.051	0.076	0.012	0.049	0.807	0.030	0.079	0.700	0.026	0.066	0.690
3:186570892	ADIPOQ rs2241766	Credible	G	T	0.010	0.033	0.768	-0.023	0.045	0.602	0.020	0.051	0.699	0.036	0.062	0.566	0.033	0.040	0.418	0.030	0.039	0.446	0.043	0.060	0.481	0.051	0.052	0.325
3:46411935	CCR5 rs1799987	Credible	G	A	0.010	0.020	0.609	0.018	0.028	0.522	-0.016	0.031	0.614	-0.017	0.035	0.621	0.004	0.025	0.867	0.001	0.024	0.974	0.004	0.035	0.900	-0.002	0.031	0.948
6:132172368	ENPP1 rs1044498	Highly credible	C	A	-0.026	0.028	0.366	-0.047	0.038	0.213	0.002	0.044	0.968	-0.027	0.050	0.596	-0.008	0.034	0.825	0.027	0.033	0.415	-0.023	0.049	0.637	0.013	0.044	0.773
6:160113872	SOD2 rs4880	Credible	G	A	0.010	0.020	0.614	0.010	0.027	0.696	0.038	0.030	0.207	-0.028	0.034	0.421	0.009	0.024	0.714	0.009	0.023	0.705	-0.034	0.034	0.311	-0.005	0.030	0.879
6:31543031	TNF-α rs1800629	Credible	G	A	0.042	0.033	0.207	0.034	0.045	0.456	0.078	0.048	0.105	0.086	0.060	0.151	0.047	0.041	0.251	0.101	0.039	0.010	0.086	0.057	0.129	0.096	0.053	0.070
7:134143958	AKR1B1 rs759853	Highly credible	G	A	0.003	0.020	0.870	0.001	0.028	0.982	0.039	0.031	0.211	0.011	0.036	0.757	0.013	0.025	0.612	-0.004	0.024	0.856	-0.013	0.035	0.719	-0.001	0.031	0.988
7:150690079	eNOS rs2070744	Credible	C	T	0.021	0.021	0.332	0.031	0.029	0.287	0.019	0.033	0.560	0.030	0.037	0.410	0.016	0.026	0.546	0.057	0.025	0.022	0.045	0.036	0.212	0.030	0.033	0.360
7:150696111	eNOS rs1799983	Credible	G	T	-0.022	0.023	0.329	-0.011	0.031	0.719	-0.002	0.035	0.945	-0.075	0.040	0.057	-0.034	0.027	0.212	-0.042	0.027	0.114	-0.091	0.039	0.019	-0.036	0.035	0.296
7:22766246	IL-6 rs1800796	Credible	G	C	0.001	0.049	0.982	0.047	0.068	0.491	-0.019	0.076	0.809	-0.005	0.107	0.965	-0.022	0.061	0.716	-0.009	0.058	0.880	0.013	0.097	0.898	0.039	0.079	0.626
7:22766645	IL-6 rs1800795	Credible	G	C	-0.022	0.020	0.288	-0.038	0.027	0.166	0.022	0.031	0.483	-0.037	0.035	0.293	-0.001	0.025	0.957	0.010	0.024	0.674	-0.022	0.035	0.518	-0.013	0.031	0.668
8:143999600	CYP11B2 rs1799998	Credible	G	A	0.005	0.020	0.824	-0.002	0.027	0.930	0.057	0.030	0.063	-0.019	0.035	0.601	0.011	0.025	0.646	0.000	0.023	0.986	-0.048	0.034	0.158	-0.002	0.031	0.949
10:114758349	TCF7L2 rs7903146	Highly credible	C	T	-0.008	0.022	0.721	0.007	0.030	0.819	-0.011	0.034	0.743	0.003	0.040	0.940	-0.006	0.027	0.839	-0.002	0.026	0.955	0.022	0.039	0.568	0.007	0.035	0.846
12:109643645	ACACB rs2268388	Highly credible	G	A	-0.002	0.030	0.957	-0.065	0.041	0.115	0.071	0.046	0.125	0.004	0.053	0.947	0.034	0.036	0.347	0.052	0.035	0.138	0.037	0.052	0.475	0.015	0.046	0.752
12:48239835	VDR rs1544410	Credible	C	T	0.030	0.022	0.180	-0.012	0.030	0.684	0.060	0.035	0.083	0.037	0.040	0.347	0.049	0.027	0.075	-0.010	0.026	0.704	0.036	0.039	0.353	0.017	0.035	0.620
16:88713236	NADPH rs4673	Credible	G	A	0.018	0.022	0.401	-0.018	0.030	0.553	0.049	0.033	0.140	0.045	0.039	0.248	0.032	0.026	0.219	0.043	0.026	0.094	0.075	0.038	0.047	0.082	0.034	0.014
17:32579788	MCP-1 rs1024611	Credible	G	A	-0.021	0.022	0.333	-0.041	0.030	0.168	-0.001	0.034	0.986	-0.046	0.038	0.224	-0.011	0.027	0.675	-0.035	0.026	0.171	-0.042	0.037	0.262	-0.046	0.034	0.173
17:61565990	ACE rs4341	Highly credible	G	C	-0.025	0.020	0.202	-0.014	0.027	0.617	-0.005	0.031	0.858	-0.044	0.035	0.198	-0.033	0.024	0.176	0.001	0.023	0.954	-0.009	0.034	0.783	0.004	0.030	0.887
17:61566031	ACE rs4343	Highly credible	G	A	-0.025	0.020	0.211	-0.017	0.027	0.539	-0.004	0.031	0.898	-0.042	0.034	0.225	-0.031	0.024	0.203	0.004	0.023	0.861	-0.005	0.034	0.884	0.007	0.030	0.826
19:41858921	TGF-β1 rs1800470	Credible	G	A	0.031	0.021	0.135	0.021	0.028	0.456	0.026	0.032	0.407	0.050	0.037	0.170	0.049	0.025	0.050	0.011	0.024	0.660	0.069	0.036	0.054	0.026	0.032	0.425
19:45411941	APOE rs429358	Credible	C	T	-0.001	0.028	0.978	0.006	0.039	0.876	-0.040	0.043	0.351	0.014	0.049	0.780	0.011	0.034	0.743	-0.046	0.033	0.167	-0.014	0.048	0.764	-0.052	0.043	0.225
19:45412079	APOE rs7412	Credible	C	T	0.031	0.038	0.412	-0.023	0.053	0.672	-0.019	0.060	0.757	0.165	0.075	0.028	0.069	0.047	0.142	0.113	0.046	0.014	0.230	0.069	0.001	0.105	0.060	0.080

SERPINE1 rs1799889 and eNOS rs869109213 were not available in the GWAS summary statistics. Rs4341 and rs4343 are two tag SNPs for ACE Ins/Del, the C allele of rs4341 and A allele of rs4341 define Ins of ACE; rs429358 defines for ε4 of APOE, rs7412 defines ε2 of APOE. StdErr, standard error.
